# Monocular visual SLAM, visual odometry, and structure from motion methods applied to 3D reconstruction: A comprehensive survey

**DOI:** 10.1016/j.heliyon.2024.e37356

**Published:** 2024-09-06

**Authors:** Erick P. Herrera-Granda, Juan C. Torres-Cantero, Diego H. Peluffo-Ordóñez

**Affiliations:** a Department of Mathematics, Escuela Politécnica Nacional, Ladrón de Guevara E11-235, Quito, 170525, Ecuador; b Virtual Reality Laboratory, ETSIIT, Department of Computer Languages and Systems, University of Granada, c/Periodista Manuel Saucedo Aranda, s/n, 18071, Granada, Spain; c SDAS Research Group, Ben Guerir, 43150, Morocco; d College of Computing, Mohammed VI Polytechnic University, Lot 660, Hay Moulay Rachid Ben Guerir, 43150, Morocco

**Keywords:** Monocular SLAM, Monocular visual odometry, Monocular structure from motion, Pure visual 3D reconstruction, Monocular RGB three-dimensional reconstruction

## Abstract

Monocular Simultaneous Localization and Mapping (SLAM), Visual Odometry (VO), and Structure from Motion (SFM) are techniques that have emerged recently to address the problem of reconstructing objects or environments using monocular cameras. Monocular pure visual techniques have become attractive solutions for 3D reconstruction tasks due to their affordability, lightweight, easy deployment, good outdoor performance, and availability in most handheld devices without requiring additional input devices. In this work, we comprehensively overview the SLAM, VO, and SFM solutions for the 3D reconstruction problem that uses a monocular RGB camera as the only source of information to gather basic knowledge of this ill-posed problem and classify the existing techniques following a taxonomy. To achieve this goal, we extended the existing taxonomy to cover all the current classifications in the literature, comprising classic, machine learning, direct, indirect, dense, and sparse methods. We performed a detailed overview of 42 methods, considering 18 classic and 24 machine learning methods according to the ten categories defined in our extended taxonomy, comprehensively systematizing their algorithms and providing their basic formulations. Relevant information about each algorithm was summarized in nine criteria for classic methods and eleven criteria for machine learning methods to provide the reader with decision components to implement, select or design a 3D reconstruction system. Finally, an analysis of the temporal evolution of each category was performed, which determined that the classical-sparse-indirect and classical-dense-indirect categories have been the most accepted solutions to the monocular 3D reconstruction problem over the last 18 years.

## 1 Introduction

In recent years, computer vision has witnessed a significant surge in the development of technologies focused on 3D reconstruction using monocular cameras. This growing interest is primarily driven by the widespread availability of affordable and lightweight monocular cameras commonly found in handheld devices. Despite their simplicity, these devices have shown remarkable capability in outdoor performance for 3D reconstruction tasks, attracting considerable attention from the research community [1].

The application of monocular 3D reconstruction spans a diverse range of fields. In robotics, it has contributed to scene understanding, allowing the advancement of autonomous driving and aiding in unmanned aerial vehicle navigation [[2], [3], [4], [5], [6], [7], [8], [9]]. The augmented reality sector has also benefited, particularly in scene reconstruction and body mapping [2,[7], [8], [9], [10], [11], [12], [13]]. The film industry has utilized these techniques for facial puppetry and reenactment [1,14,15], and the medical field has seen advancements in the 3D reconstruction of surgical cavities and virtual endoscopy [[16], [17], [18], [19]]. Thus, several solutions have been proposed to address the 3D reconstruction problem, notably monocular Simultaneous Localization and Mapping (SLAM), Visual Odometry (VO), and Structure from Motion (SFM). Each technique brings a unique perspective and methodology to the challenge of reconstructing environments and objects using monocular cameras.

VO is a discipline focused on ego-motion estimation, developed over the need to determine the robot's position and orientation using camera images, where 3D reconstruction is commonly used as a map-generating step from where vehicle location is estimated. There exists a wide range of solutions that can be used to perform odometry tasks, like wheel odometry, global position systems (GPS), global navigation satellite systems (GNSS), inertial navigation systems (INS), laser sensors, ultrasonic sensors, and VO [20]. Wheel odometry typically uses encoders, which are low-cost solutions; however, it produces position drift due to wheel slippage. INS are used to calculate position and orientation over three axes through accelerometers and gyroscopes, as the position is obtained by solving second-order integrals causing position drift due to minor errors in acceleration that can produce significant errors in position. GPS/GNSS obtain position information by trilateration of information obtained from three or more satellites, avoiding error accumulation over time; however, position measures obtained by GPS/GNSS can present meter-range errors and cannot be used for indoor or underwater applications. Similarly, laser and ultrasonic sensors provide scalar distance measurements from the sensor to the target using time-of-flight or phase shift principles, but they also present reflection problems depending on the material or orientation of the target surface. In contrast, VO is a low-cost solution more accurate than GPS, INS, ultrasonic sensors, and wheel odometry due to its low position error range from 0.1 to 2 % [21]. As an odometry solution, VO is characterized by a satisfactory balance between cost, reliability, and implementation complexity [22]. Furthermore, studies like [23,24] established that VO obtains better results by generating geometric maps of the environment from which the robot could be localized.

SLAM is the ability developed for a mobile robot to start in an unknown environment and incrementally build a map using the information obtained from camera observations and simultaneously compute the estimated trajectory using single or multiple cameras [25,26]. Moreover, SFM algorithms have been referred to as Monocular SLAM [27] due to their similarity in obtaining a geometric representation by generating and tracking sparse or dense world models using direct or indirect techniques.

The primary distinction between Visual Odometry (VO) and Simultaneous Localization and Mapping (SLAM) methods lies in the characteristic that in VO, the points are typically not reused once they exit the field of view. In contrast, SLAM typically enables the recycling of previously triangulated points as they reenter the field of view in loop-closure steps [20]. Another major difference between these disciplines is that SLAM performs both tasks simultaneously, generating a map of the environment and using this map to estimate the camera pose. SFM focuses more on generating a scene map, while VO focuses more on estimating the pose, also called ego-motion (translation and orientation of an agent) [21]. It must be mentioned that SLAM methods consist of larger systems developed to obtain a geometric representation and provide a sense of orientation (typically as a camera pose), trajectory, and loop closure, among others. In SLAM systems, this information is used for navigation tasks and scene comprehension, being complex systems, and sometimes, this information is employed for geometric map optimization tasks. In addition, the development of multiple SLAM systems was inspired by VO and SFM formulations [3,[28], [29], [30]] extending their pipelines to estimate ego-motion and geometry simultaneously. Thus, irrespectively of the denomination that may be given to every SLAM, VO, and SFM approach, all of them hold the common goal of both 3D scene reconstruction and camera pose estimation using imaging sensors, but with further specialization in the main task for which each was designed [2,20,24].

Despite their strengths, these methodologies face several limitations. Monocular pure visual 3D reconstruction involves some critical and well-known problems related to the lack of scale information and the computational complexity of recovering 3D models from sequences of monocular images without geometric priors. Nowadays, available monocular methods still lag behind controlled multi-view setups in quality, accuracy, and completeness [1]. Simplifications in real-world image formation processes, such as assuming low-frequency, distant illumination and neglecting shadows and global illumination effects, limit the accuracy of reconstructions. Additionally, a significant challenge arises in the presence of purely rotational movements in the absence of a stereoscopic baseline, which complicates the accurate capture and modelling of three-dimensional structures, as the lack of a baseline hinders the effective differentiation of depth and spatial relationships.

Another well-identified challenge across SLAM, VO, and SFM is the absence of a comprehensive taxonomy that categorizes the vast array of existing solutions. This lack of classification and joint analysis poses a significant barrier to advancing the field. Moreover, each discipline has its own set of limitations. For instance, while producing superior 3D reconstruction results, SLAM systems often struggle with long-term operation and large loop closures, especially in dynamic environments [31]. VO methods, on the other hand, are sensitive to varying operating conditions, such as lighting and textures, affecting their robustness [20]. SFM techniques, while excelling in structure recovery, do not fully leverage the advantages of simultaneous pose and geometry recovery [32].

To address these challenges, our research conducted a comprehensive overview of the SLAM, VO, and SFM solutions for monocular 3D reconstruction, with a particular focus on those based on a monocular RGB camera as the sole source of information. In contrast to previous surveys like [20,31,[33], [34], [35], [36]], our work extends the existing taxonomy, including a broader range of classifications, such as classic, machine learning, direct, indirect, dense, and sparse methods, which allows to cover multiple new proposals like hybrid, classic, and machine-learning based systems and their variants. This new taxonomy represents a significant advancement in organizing and understanding the field, considering techniques' rapid evolution and diversity. Our detailed review of 42 methods is more extensive than most existing surveys, covering classic and machine-learning approaches. We provide systematic categorization and essential formulations for implementing, selecting, or designing a 3D reconstruction system. Furthermore, our work includes a time citation evolution analysis, a novel aspect not commonly found in other reviews, offering insights into the acceptance and evolution of these methods over the past 18 years. The here-presented analysis and comparative study provide insights into algorithm selection, which makes this work a valuable resource for researchers and practitioners.

As a notable contribution, our review work presents a more exhaustive effort in categorising these methods compared to previous works because it comprehensively covers the main 42 monocular methods, allowing to gather their foundations, formulations, algorithm design, and extract categorical selection criteria. For instance, we not only focus on SLAM, VO, and SFM individually but also explore their interrelations and implications for monocular 3D reconstruction. This holistic view is absent in many existing surveys, which often concentrate on isolated aspects of the problem. This way, our research extends beyond existing literature by providing an updated, detailed, and comprehensive overview that integrates various aspects of monocular 3D reconstruction. Our novel taxonomy, extensive methodological review, and time citation analysis constitute the main contributions, setting our work apart from existing surveys and reviews in the field.

This work was specifically undertaken to delve into the methodologies for creating 3D geometric representations (reconstructions) of an environment using a single moving monocular RGB camera. As such, the investigation excluded techniques beyond the scope of this study, such as Visual-Inertial (utilizing Inertial Navigation Systems for enhanced tracking), RGB-D (employing additional sensors to capture depth information from the surroundings), omnidirectional, and stereo techniques.

## 2 Related works

In the literature, since monocular 3D reconstruction is an ill-posed problem that can be solved by combining different techniques and algorithms from disciplines like computer vision, robotics, and machine learning, only a few works can currently be related to this overview. In 2016, Aqel et al. [20] developed a comprehensive review of visual odometry types, approaches, challenges, and applications. In this work, authors described the different input modalities and motivations for studying the VO problem, so a first taxonomy was established to define the difference between feature- and appearance-based approaches. The differentiating factor with our approach is that we covered SLAM, VO, and SFM problems. In contrast, only the VO problem was explored in the work mentioned above, not focusing on the monocular input mode. In 2021, Servières et al. [31] proposed a detailed review of the state-of-the-art classification and experimental benchmarking of visual and visual-inertial SLAM techniques, detailing the basic structure of SLAM system, SLAM evolution problem beginning from its appearance, then introducing the visual SLAM and visual-inertial SLAM, addressed using direct and indirect classification. In addition, an experimental benchmark was carried out using DSO [24], LSD-SLAM [37], ORB-SLAM2 [38], ROVIO [39], and Vins-Mono [40] systems. In contrast to Ref. [31], we are not only focused on the SLAM approach but are especially interested in 3D reconstruction, so SLAM, VO, and SFM are suitable for our work. In addition, we are not only interested in the description and historical appearance of each method; instead, we are primarily focused on providing an appropriate taxonomy and describing each of the most important algorithms to give the reader an appropriate overview that can help them to correctly select the most appropriate method for projects and research.

In the survey articles category, a few works address the pure visual monocular approach. In the Taketomi et al. study [33], a survey focused on the 2010 to 2016 period, developed specifically for the visual SLAM (vSLAM) approach. This study described the main elements of the vSLAM problem and adopted the feature-based and direct classification to briefly overview the main SLAM systems from 2010 to 2016. In this way, MonoSLAM [28], PTAM [41], and ORB-SLAM [11] were addressed as feature-based techniques, whereas DTAM [27], LSD-SLAM [3], SVO [23], and DSO [24] were described as direct formulations. In addition, the Taketomi et al. survey detailed the KinectFusion [42], Dense visual SLAM [43,44], Elastic Fusion [44], and SLAM++ [45] as RGB-D techniques. Another important survey that might be considered is the work of [36], which presents a survey in monocular SLAM algorithms for outdoor applications, mainly focused on natural environments requiring long-range capable techniques that are not affected by sunlight and can manage textured images with plenty of vegetation. The authors selected the most prominent algorithms available until 2018, so DSO [24], ORB-SLAM [11], and LSD-SLAM [3] were selected. These algorithms were reviewed and compared using the RMSE of the translational error over eight sequences recorded by the authors. Results evidenced that DSO was the method that performed better for outdoor applications. However, there was a lot of work that could be done to enhance reconstruction and tracking quality. In addition, the work [34] presents a survey of the main visual SLAM systems, focused on the methods that made considerable contributions before 2018. The survey summarizes the SLAM history, its relationship with the SFM and VO problems, the SLAM classification, the key issues, and improvement trends. The study adopted feature-based and direct classification, where many existing SLAM systems were mentioned. However, the authors reviewed the PTAM [41] and ORB-SLAM [11] as feature-based approaches and the DTAM [27], SVO [23], LSD-SLAM [3], and DSO [24] as direct approaches. One of the most recent surveys performed for monocular pure visual systems is the study of [46], which mainly focused on the monocular visual odometry problem, where authors described the VO problem, its formulation, and its similarities with the most recent SLAM proposals. In addition, the authors overviewed the MonoSLAM, PTAM, DTAM, KinectFusion, DVO, SVO, LSD-SLAM, DSO, and eight additional RGB-D methods. In contrast with the mentioned surveys, we performed a comprehensive and consistent survey of 42 algorithms, not only focused on a type of problem (SLAM, SFM, or VO) or a specific category of the classification. Instead, we worked with a completely extended classification considering all the monocular pure visual systems contributing to the monocular 3D reconstruction problem.

The work that we can refer to as closest to our approach is the article developed by Macario et al. [35], which is a comprehensive survey developed for visual SLAM algorithms where authors provide the reader with a set of initial concepts, a taxonomy based on direct and indirect classification, a review of eight visual-only SLAM methods, six visual-inertial SLAM methods, five RGB-D SLAM methods, and finally the discussion of open problems and open directions. The work was comprehensively explained, and each algorithm addressed was adequately described and systematized. We followed a similar direction, focusing our investigation on the monocular input mode, so we further analyzed this modality, properly defining and applying a taxonomy that better characterizes each algorithm. In addition, 42 of the most representative algorithms available are analyzed in depth, including an exhaustive overview of the newest Machine Learning-based (ML) approaches that had not been discussed in any of the cited related works.

## 3 Contributions and outline

To the best of our knowledge, this is the first overview specialized in the monocular 3D reconstruction problem that considers SLAM, VO, and SFM solutions, integrating them into a complete taxonomy. To summarize, the key contributions of our work are:

1) A taxonomy designed to contain all possible current existing literature approaches built considering three classifications and all possible combinations defining ten categories overviewed in detail.
2) A comprehensive overview of the most representative 42 monocular SLAM, VO, and SFM algorithms comprised by 18 classic monocular methods and 24 methods that integrate machine learning techniques. Each examined method includes its algorithm systematization, including mathematic principles for those algorithms that made innovations in their formulations, especially regarding depth map estimation or optimization, since this work is focused on 3D reconstruction.
3) We defined 11 criteria to provide the reader with the components to implement, select or design a 3D reconstruction system, nine applicable to classic systems and two additional criteria only applicable to machine learning approaches. Information was gathered for each algorithm and is presented in Table 2, Table 3, where the criteria are: type of algorithm (SLAM, VO, or SFM), tracking method (direct vs. indirect), map density (dense vs. sparse), pixels used (the technique to extract pixel information), estimation method (depth map estimation technique), global optimization, relocalization, loop closure (whether or not the algorithm includes optimization, relocalization or loop closure steps), availability (open source repository where the algorithm is available), CNN architecture (commonly known employed CNN architecture) and the main tasks for which a CNN was employed.

**Table 1:** Input modalities used in 3D reconstruction.

Type of camera	Pros	Cons
Stereo	Depth information and image scale are computed instantaneously. Depth information is easily obtained Provide 3D information	Require more calibration effort and are more expensive than monocular ones. Difficult shutter synchronization It gets degraded to a monocular device when the stereo baseline is smaller than the camera-to-object distances.
Omni-directional	Present a larger FOV close to 360°, and images provide more information. Features remain longer in the image, helping to obtain well-refined models.	More expensive than the rest Not compatible with mobile devices Some may not work in dynamic environments. Present distortions that come from the equirectangular representation
Monocular RGB-D	Provide depth measurements with each image in real-time rates Easy deployment Compatible with mobile devices Suitable for small robotics and indoor environments	Produce erroneous measurements under sunlight. Limited by the range of the active sensor and the size of the projected pattern More expensive than monocular RGB sensors
Monocular RGB	Lowest cost Ubiquitous Easy deployment Available on most mobile devices Simple calibration Not limited by sensor range Suitable for small robotics in indoor and outdoor environments Can work under sunlight	Presents image scale uncertainty. Does not provide depth measurements. Reconstruction tasks may consume more computational resources.

**Table 2:** Input modalities used in 3D reconstruction.

Method	SLAM, VO or SFM	Tracking method	Map density	Pixels used	Estimation	Global optimi-zation	Relocal-ization	Loop closure	Availa-bility
Jin et al. (2000) [58]	SFM	Feature-based	Sparse	Hi.grad.	EKF	–	–	–	–
MonoSLAM (2007) [28]	SLAM	Feature-based	Sparse	Shi Tomasi	EKF	–	–	–	[113]
PTAM (2007) [71]	SLAM	Feature-based	Sparse	Hi.grad.	BA			–	[114]
OpenMVG (2013) [73]	SFM	Feature-based	Sparse	Hi.grad.	BA		–		[115]
ORB-SLAM (2015) [11]	SLAM	Feature-based	Sparse	Hi.grad.	Local BA				[116]
COLMAP (2016) [77]	SFM	Feature-based	Sparse	PnP matches	Local and Global BA		–	–	[117]
ORB-SLAM2 (2017) [38]	SLAM	Feature-based	Sparse	Hi.grad.	Local BA				[118]
ORB-SLAM3 (2021) [81]	SLAM	Feature-based	Sparse	Hi.grad.	Local BA				[119]
Valgaerts et al. (2011) [85]	SFM	Optical flow	Dense	8-point matches	Robust 8-point algorithm	–	–	–	–
Ranftl et al. (2016) [88]	SFM	Optical flow	Dense	FlowFields	Superpixel graph minimization	–	–	–	–
Stühmer et al. (2010) [91]	SLAM	Direct	Dense	Hi.grad.	Cost volume refinement			–	–
DTAM (2011) [27]	SLAM	Direct	Dense	Hi.grad.	Cost volume refinement			–	[120]
REMODE (2014) [4]	SLAM	Direct	Dense	Hi.grad.	Bayesian estimation	–	–	–	[121]
LSD-SLAM (2014) [37]	SLAM	Direct	Semi-Dense	Edgelets	Pose graph optimization		–		[122]
DSO (2017) [24]	VO	Direct	Sparse	Hi.grad.	Local BA	–	–	–	[123]
LDSO (2018) [64]	VO	Direct	Sparse	Hi.grad.	Local BA GPGO		–		[124]
DSM (2020) [30]	SLAM	Direct	Sparse	Hi. grad.	Photometric BA		–	–	[125]
SVO (2014) [23]	VO	Hybrid	Sparse	FAST + Hi. grad.	Local BA	–	–	–	[126]

Hi.grad. is used to abbreviate a set of pixels with high-intensity gradient.

EKF is used to abbreviate the Extended Kalman Filter technique.

BA is used to abbreviate the Bundle Adjustment technique.

GPGO is used to abbreviate the Global Pose Graph Optimization technique.

**Table 3:** Summary of the most representative ml monocular SLAM, VO, and SFM systems.

Method	SLAM, VO or SFM	Tracking method	Map density	Pixels used	Estimation	CNN architecture	CNN's main estimation tasks	Global optimi-zation	Reloca-lization	Loop closure	Avai-lability
DynaSLAM (2018) [102]	SLAM	Feature-based	Sparse	Hi.grad.	Local BA	Mask R-CNN	Instance segmentation				[192]
BA-Net (2019) [59]	SFM	Feature-based	Sparse	Hi.grad.	BA	DRN-54	Depth Damping factor	–	–	–	[138]
Steenbeek et al. (2022) [6]	SLAM	Feature-based	Sparse	Hi.grad.	BA	ResNet-50 Enc.dec.	Scale Depth map densify				[193]
Sun et al. (2022) [60]	SLAM	Feature-based	Sparse	Hi.grad.	BA	ResNetXt-50 Enc.dec.	Scale Relative depth Depth				–
Lee et al. (2022) [159]	SLAM	Feature-based	Sparse	Hi.grad.	BA	Enc. dec.	Scale Semantic segmentation Feature refinement				–
SVR-Net (2023) [161]	SLAM	Feature-based	Sparse	Learned features	Optimal match recurrent network	ScanNet	Local map Relative pose TSDF values		–	–	–
DeMoN (2017) [141]	SFM	Optical flow	Dense	SIFT keypoints matching FlowFields	8-point algorithm RANSAC	Chain Enc.dec.	Optical flow Depth Pose Surface normals	–	–	–	[108]
DeepV2D (2020) [144]	SLAM	Optical flow Feature-based	Dense	Learned features	3D Stereo matching over cost volumes	Residual Flow Hourglass Enc.dec.	Depth Pose 3D stereo matching		–	–	[106]
VOLDOR (2020) [163]	VO	Optical flow residuals	Dense	Learned features	Generalized Expectation-Maximization	PWC-Net	Optical flow	–	–	–	[104]
DROID-SLAM (2021) [167]	SLAM	Optical flow	Dense	Learned features Between keyframes edges	BA	Residual blocks	Feature extraction Optical flow Estate estimation		–		[194]
SDF-SLAM [146]	SLAM	Feature-based	Dense	Learned features and descriptors	BA	Enc.dec.	Feature and descriptor extraction Semantic segmentation				–
NeRF-SLAM (2022) [169]	SLAM	Optical-flow	Dense	Learned features Between keyframes edges	BA Radiance field optimization	Residual blocks Neural Radiance Fields	Feature extraction Optical flow Estate estimation		–		[195]
Rosinol et al. (2023) [170]	SLAM	Optical-flow	Dense	Learned features Between keyframes edges	BA Probabilistic volumetric fusion	Residual blocks	Feature extraction Optical flow Estate estimation		–		–
CNN-SLAM (2017) [2]	SLAM	Direct	Semi-dense	Hi.grad.	Pose Graph optimization	ResNet-50 FCN	Depth Semantic segmentation		–		[105], a
DeepTAM (2018) [171]	SLAM	Direct Optical flow	Dense	Hi. grad.	Cost volume refinement	Enc.dec.	Pose hypotheses Optical flow Depth Depth refinement			–	[111]
DeepFusion [174]	SLAM	Direct	Semi-dense	Hi. grad.	Opt framework	U-Net	Log-depth gradients and uncertainties Scale		–	–	–
CodeSLAM (2018) [134]	SLAM	Direct	Dense	Hi. grad.	BA	U-Net Variational Enc.dec.	Code Compact depth		–	–	[107], b
DeepFactors (2020) [10]	SLAM	Direct	Dense	Hi. grad.	Multiview BA	U-Net Variational Enc.dec.	Code Compact depth Uncertainty				[112]
DVSO (2018) [140]	VO	Direct	Sparse	Hi. grad.	BA	ResNet-50 Encoder-decoder	Disparity maps	–	–	–	[110], c
CNN-DVO (2020) [103]	SLAM	Direct	Sparse	Hi. grad. Dynamic upsampling and downsampling	BA	U-Net Encoder-decoder	Depth		–		[191]
D3VO (2020) [139]	VO	Direct	Sparse	Hi.grad.	BA	U-Net Encoder-decoder	Depth, Pose, Uncertainty	–	–	–	–
MonoRec (2021) [182]	SFM	Direct	Sparse	Hi. grad. Mask filter	BA	U-Net ResNet-18 features Encoder-decoder	Depth Mask Moving objects	–	–	–	[109]
DDSO (2022) [145]	VO	Direct	Sparse	Hi. grad.	BA	ResNet-50 Encoder-decoder	Depth Pose Transformations	–	–	–	–
CNN-SVO (2019) [66]	VO	Hybrid	Sparse	FAST + Hi.grad.	Local BA	ResNet-50 Encoder-Decoder	Depth	–	–	–	[196]

Hi.grad. is used to abbreviate a set of pixels with a high-intensity gradient.

Enc.dec. is used to abbreviate the Encoder-decoder CNN architecture.

EKF is used to abbreviate the Extended Kalman Filter technique.

BA is used to abbreviate the Bundle Adjustment technique.

a Unofficial implementation of the CNN-SLAM method. There is no official implementation of this method yet.

b Unofficial implementation of the Code-SLAM method. There is no official implementation of this method yet.

c Unofficial implementation of the DVSO method. There is no official implementation of this method yet.
4) A discussion of open questions, available solutions, future directions for each classification and temporal analysis of the citation scores achieved by each taxonomy category give the reader an intuition of the impact and acceptance that each classification, method and category has generated in this research field.

This paper is organized as follows: Section 4 discusses input modalities, section 5 introduces the basics, notation, and taxonomy, section 6 presents an overview of the most representative classic methods, section 7 introduces an overview of the most representative methods that integrate machine learning, section 8 provides a discussion made over the entire taxonomy. Finally, conclusions are presented in section 9.

## 4 Input modalities

The goal of obtaining a 3D geometric representation of the scene is a complex task that can be achieved using camera sensors. In the past, some state-of-the-art systems were built using complex camera arrays and lightning setups, primarily for indoor applications. Nowadays, different capture devices range from costly multi-view and stereo setups to cheap monocular sensors. Next, a brief overview of several input modalities used for 3D reconstruction is introduced.

### 4.1 Stereo setups

Multi-view setups consist of an array or a set of pairwise stereo cameras distributed in a configuration that allows the capture of simultaneous views of the same object at the same time [1]. Remarkably, stereo systems that use a pair of cameras are called stereo systems [20]. Binocular cameras have two separate camera sensors, so in-depth information and image scale can be computed instantaneously by triangulation due to their fixed and known stereo baseline size. However, these cameras are generally more expensive than monocular sensors and require more calibration effort. In addition, these sensors must acquire images at the same time interval, which can be achieved by synchronizing shutter speed using an external trigger signal [47]. Generally, maintaining a calibrated constant baseline between two cameras requires much more effort than in the monocular case stereo as setups are degraded to monocular when the baseline is much smaller than the distance from the scene to the camera [48]. Hence, they are limited to working in small and indoor environments.

### 4.2 Omni-directional cameras

Many authors decided to utilize omnidirectional cameras, like [[49], [50], [51], [52]], due to their wide field of vision (FOV). According to Ref. [51], Omnidirectional cameras provide more information than regular cameras, and the features found in the images stay longer, helping to obtain well-refined 3D scenery models. However, Omnidirectional cameras are expensive, require a lot of setup effort, and are incompatible with mobile devices. Furthermore, some of these devices incrementally scan the scene using mechanical rotation, so these devices are intended to work in static conditions and may not work in dynamic environments [53]. One of the main problems these devices have for the 3D reconstruction task is the generation of distortions in the image that comes from the equirectangular representation, which happens when the obtained spherical pixels projected to a plane get significantly distorted, which can cause depth prediction errors [53].

### 4.3 Monocular RGB-D

RGB-D sensors have an additional active or passive sensor that allows the system to get depth measurements of the environment associated with each image pixel in real-time. Such depth measurements help solve the monocular reconstruction's depth and scale ambiguity because they can be used as an estimate of the environment geometry. These devices can be classified as passive or active. In contrast to stereo sensors, passive RGB-D cameras typically present a projector instead of a second camera, projecting a pattern in the image to find coincident points. To prevent this pattern from being distinguished by the human eye, these sensors commonly work in the infrared domain (IR). RGB-D cameras using infrared projectors known as active. It must be mentioned that active RGB-D cameras can produce erroneous measurements under sunlight [2] due to sunlight IR radiation that overpowers the projector. For this reason, some RGB-D cameras present a combination of active and passive sensors that are activated whether under sunlight or not, which can significantly increase the cost of this kind of device. Another common limitation of these light devices is that they cannot reconstruct objects smaller than the projected pattern [1].

Time of Flight (ToF) is another active RGB-D camera that gets the depth measurements by emitting a light pulse and computing the time it takes to reach the object. This time measurement is challenging because this event occurs at the speed of light. That is why ToF RGB-D cameras typically perform worse than RGB-D light ones [1].

### 4.4 Monocular RGB

RGB cameras capture the intensity of the received light in three channels: red, green, and blue. RGB devices are designed to work in different configurations, ranging from CCD sensors acquiring each signal in a separate sensor to Bayer pattern-based sensors where colour filters are interleaved in front of a single sensor [1]. Monocular cameras are known for reducing the effect of calibration errors. They are ubiquitous, low cost, easy to deploy, and available in most portable devices, which is considered a significant incentive that has kept the attention of researchers, who usually prefer this type of input to perform reconstruction tasks involved in SLAM, SFM, and VO. Nevertheless, this input involves an ill-posed problem because monocular vision suffers from scale uncertainty [54,55], and the techniques used to achieve the goal of 3D reconstruction typically require a lot of computational resources. Table 1 summarizes the features and drawbacks of using the different input modalities for 3D reconstruction.

As noticed in Table 1, although monocular RGB cameras present some important challenges due to their sensor less nature, not allowing them to provide direct depth measurements, they present the most attractive set of pros, especially for small robotics applications. Monocular RGB sensors present the lowest price among all the available camera types, are easy to deploy, and are compatible with almost all the existing portable devices and processors, like Single Board Computers (SBC) and Field Programmable Gate Arrays (FPGA), which makes this input modality especially attractive for researchers. This is why the research was developed, focusing on the monocular RGB input modality.

## 5 Basics and notation

### 5.1 Literature review process

**Fig. 1:**
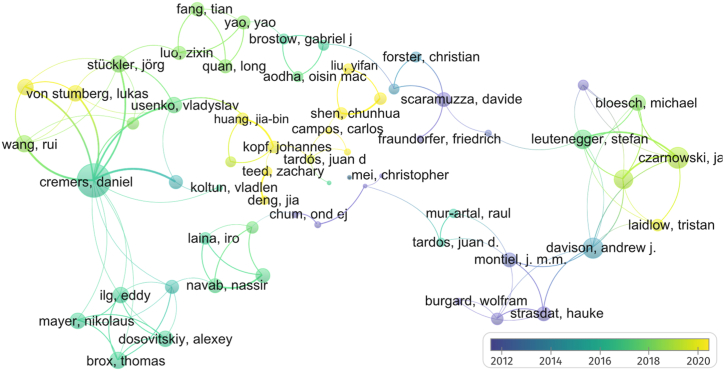
Bibliometric co-authorship analysis results were executed in VOSviewer using the association strength method, full-count strategy, and at least two documents per author.

Scene- 3D reconstruction using monocular cameras is a complex challenge that has kept the interest of many researchers. However, there are few studies in this discipline, and as an ill-posed problem, each approach is considerably different from the rest, so the terminology could be used in varied ways. For that reason, the main objective of this paper was to establish a taxonomy and provide the reader with some basics about the discipline. In this way, we used the following search criteria in Scopus and Scholar Google, applied to the time range from 2000 to April of 2023, performing the search only for the English language: TITLE-ABS-KEY (("SLAM" OR "VO" OR "SFM" OR "Simultaneous Localization and Mapping" OR "Visual Odometry" OR "Structure from Motion")) AND ("Monocular" OR "Visual" OR "RGB") AND NOT ("RGB-D" OR "Stereo" OR "omnidirectional" OR "Visual Inertial" OR "VI")). After complete and independent reading, we set apart works that applied RGB-D, stereo, or omnidirectional cameras, obtaining a total of 137 studies focused on monocular RGB 3D reconstruction. The bibliometric information of each article was extracted using Scopus and Mendeley, so a .ris file was extracted, and its information was processed using VOSviewer software [56]. Bibliometric co-authorship analysis was set under selected bibliometric data, association strength method, full-count strategy, and a minimum of two documents to consider an author, so 64 authors met the threshold. Results obtained on bibliometric analysis are illustrated in Fig. 1.

As can be seen, the bibliometric analysis helped us to find the authors with the greatest link strength based on their contribution to the state of the art, so the authors with the highest overall link strength were Cremers, D., Czarnowski, J., Davison, A., Clark, R. and Leuteneger, S., with link strengths of 28, 13, 12, 11 and 11 respectively. In this way, our literature review strategy was guided by an exhaustive reading, starting with the most representative authors, in order to gather basic knowledge. Then, we kept extending the bibliographic database according to the studies cited in each study as previous contributions and studies that were used for comparisons.

### 5.2 Notation

Throughout the paper, bold lowercase letters (x) represent vectors for statements and formulations. Bold uppercase letters (R) represent matrixes. Scalars are represented by light lowercase letters (c). Functions and images are represented by upper-case light letters (I). Let us define image I containing a set of pixels. For each q pixel in the image, let us assume that there is a d depth value allowing for the projection of its corresponding 3D coordinates $x={\left(x,y,z\right)}^{T}$. In this way, camera poses are represented as transformation matrixes $T_{i}\in SE\left(3\right)$, transforming a point from the real-world frame to the camera frame. R represents rotation matrixes, while Π and $\Pi^{-1}$ are projection and back projection functions. Additionally, $d^{*}$ represents inverse depth values, so D and $D^{*}$ correspond to depth and inverse depth maps.

### 5.3 Initial words and approaches

One of the main motivations of visual SLAM, SFM, and VO is Camera pose estimation, which initially used to be approached in three ways: using a feature-based approach, an appearance-based approach, or by a hybrid feature- and appearance-based approach [20,21,51].

**Fig. 2:**
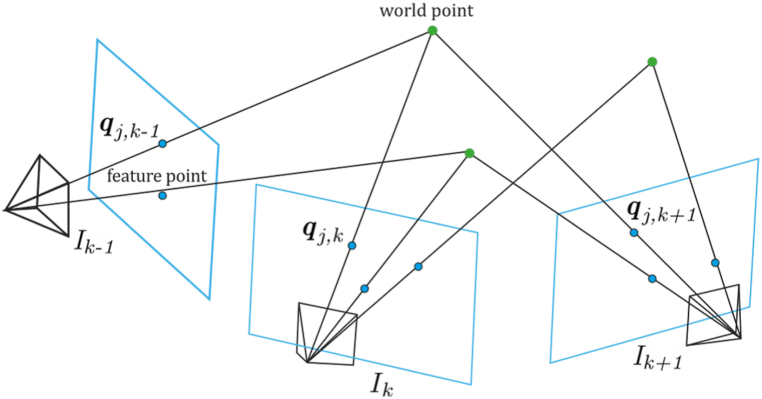
Feature matching and 3D position triangulation from multiple views.

Feature-based approach, as implemented in Refs. [10,11,38,[57], [58], [59]], typically performs image feature extraction (corner, lines, curves, among others), representative features matching, and motion estimation tasks. Matching is performed using the Euclidean distance of the feature vectors of both images to find matching candidates. In this way, given two images of the same scene at different poses, the first image features are matched with their corresponding features of the second image, thus providing a 3D position of the points related to those features, as depicted in Fig. 2. Motion is typically estimated by observing the feature displacement, where the camera pose is determined by finding a geometric transformation between each pair of images using a set of correspondent features.

**Fig. 3:**
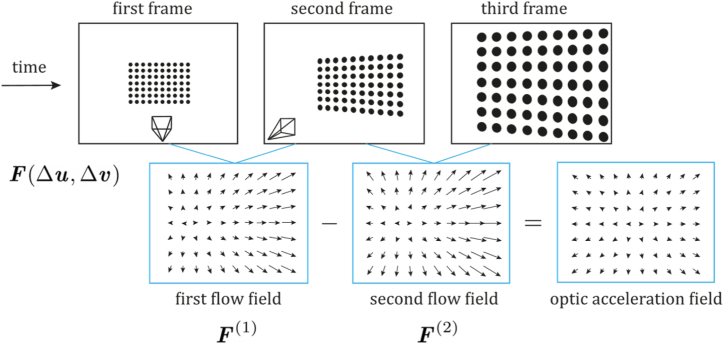
Optical flow field and optical flow acceleration field generation from consecutive frames. Where F is the optical flow field, and Δu and Δv are the pixel displacements of the template.

While the feature-based approach tracks a set of features, appearance-based methods monitor the appearance changes of each acquired image using pixel intensity information. Therefore, an alternative to working with pixel intensity is computing the optical flow, which can be used to estimate camera motion and speed. Optical flow (OF) computes the displacement of brightness patterns from one image to the next, using neighbouring pixels' intensity values. Depending on the number of pixels used to estimate camera motion, OF algorithms can be classified as dense or sparse. Dense methods do not require a feature extraction stage since they use the full image information, making them less robust to noise than sparse ones [60]. A commonly used method for appearance-based ego-motion estimation is template matching, consisting of selecting a template from the first image and trying to match it to the next frame. Template matching is based on determining whether a sub-image, named template, exists or not in a larger image called search area by calculating similarity measures like normalized cross-correlation (NCC), the sum of square or absolute differences (SSD/SAD). The algorithm shifts the template over the search area, and the position that presents the higher similarity measure is the position of the template found in the new image [20]. Next, the pixel displacements of the template Δu, Δv are declared as vectors, thus defining the velocity and subsequent acceleration in the flow field as depicted in Fig. 3. These displacements can be converted to horizontal and vertical physical displacements using camera calibration parameters for different applications.

According to Refs. [61,62], feature-based approaches tend to fail in low-textured environments (e.g., walls, roads) due to the small number of salient features that can be recognized and tracked. On the other hand, appearance-based approaches are more robust and allow better tracking in low-textured environments [61]. However, they are sensitive to photometric changes and require excellent initialization for good results. For that reason, systems like the Scaramuzza & Siegwart proposal [50] were built using a hybrid of feature- and appearance-based approaches.

Considering that 3D reconstruction is a constitutive challenge for monocular SLAM, SFM, and VO techniques, the previous classification of feature, appearance, or hybrid approaches is unsuitable to contain this wide variety of solutions for this monocular ill-posed problem. Thus, a better categorization could be found in the works of [24,25,27,37,[63], [64], [65]], where two main classifications emerge: direct and indirect, dense, and sparse.

**Fig. 4:**
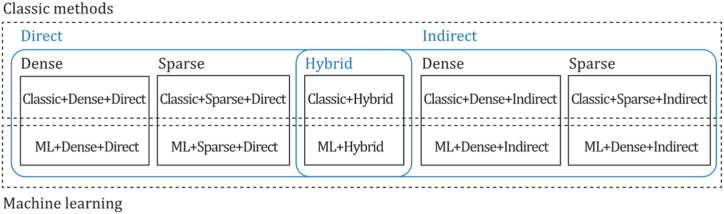
Extended taxonomy for monocular 3D reconstruction methods.

Consequently, monocular SLAM, VO, and SFM are divided into two main categories, depending on the number of features used for tasks required in 3D reconstruction. Hence, sparse and dense classification is a way to categorize them. In addition, another classification depends on the need to perform preprocessing before obtaining the actual parameters and measurements. Indirect methods use this preprocessing step, which generates an intermediate representation of noisy measurements to be optimized before estimating geometry and camera motion. In contrast, direct formulations use pixel information directly. In addition, due to the major improvements in machine learning and the impressive results this new category has achieved, a new component for the taxonomy was introduced, as depicted in Fig. 4. In this way, we established three classifications to determine an appropriate taxonomy for the monocular 3D reconstruction problem: direct vs. indirect, dense vs. sparse, and classic vs. machine learning.

• **Direct vs. Indirect.** Direct techniques do not use preprocessing steps like the feature or optical flow extraction, whereas indirect techniques use these preprocessing steps.
• **Dense vs. Sparse.** Dense refers to techniques using the entire or most of the image pixel information, whereas sparse refers to techniques using only a subset of selected pixel information.
• **Classic vs. Machine Learning.** Classic methods, also known as geometric-based methods, rely on geometry, odometry or probabilistic suggestions to run their entire pipeline. They do not require any learning steps, so they must be appropriately tuned and calibrated for functioning, compared to learning-based methods, which have demonstrated their power in performing low-level tasks (feature extraction, depth estimation, pose estimation) and high-level tasks (classification and semantic segmentation). For that reason, many researchers have focused on developing neural networks for pose prediction, depth prediction, feature extraction, and semantic segmentation (among others) combining them with classic frameworks to enhance their accuracy, generalization capabilities, robustness, scene comprehension capabilities, and the like.

**Fig. 5:**
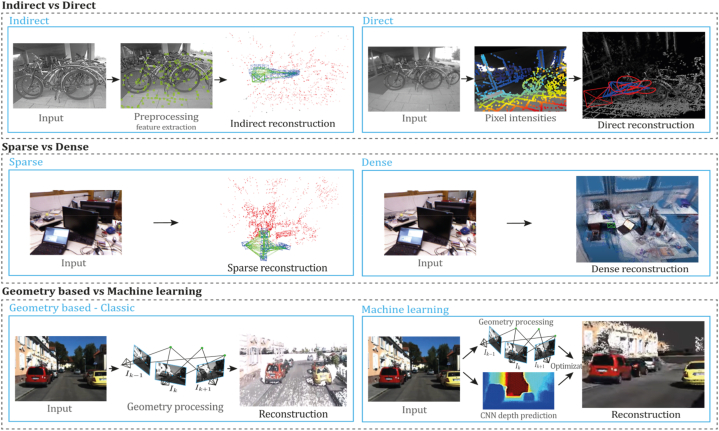
Classifications for monocular 3D reconstruction systems. The top examples represent direct and indirect classification, while the middle examples belong to sparse and dense classification. The examples below correspond to classic and machine learning classification. Examples were obtained by implementations of ORB-SLAM2 [21], DSO [2], LDSO [10], and MonoRec [43] on the datasets TUM-Mono [44] sequence 42, TUM-RGB-D [45] sequence Freiburg-1-room and KITTI [46] sequence 7, respectively.

Fig. 4 depicts the three classifications considered in this study. Combining these three forms of classification and considering that there are hybrid approaches [23,66] combining direct and indirect techniques to perform the reconstruction task, we propose the following taxonomy: Classic + Dense + Direct, Classic + Sparse + Direct, Classic + Dense + Indirect, Classic + Sparse + Indirect, Classic + Hybrid, ML + Dense + Direct, ML + Sparse + Direct, ML + Dense + Indirect, ML + Classic + Sparse + Indirect and ML + Hybrid. The classification of the currently existing methods is depicted in Fig. 5.

## 6 Classic methods

Classic monocular SLAM, SFM, and VO techniques, also known as the geometric-based approach, are the first categories addressed in this study. These methods are called geometric-based approaches, usually relying on geometric information to perform 3D reconstruction running their entire SLAM, VO, or SFM pipelines. However, since recovering the scene geometry as a reconstruction is an ill-posed problem, many proposals may combine traditional geometric techniques with others, including probabilistics, optimization, computer vision, heuristic techniques, and so on. Nevertheless, in this classification, we do not include formulations that use machine learning, which will be discussed in Section 7.

As mentioned by Engel et al., the scene geometry can be estimated using a probabilistic model that uses noisy measurements Y from the images, generating an X estimator for the 3D model and the ego-motion [24]. This way, the 3D reconstruction problem can be solved using classic approaches from indirect and direct paradigms. In the indirect approach, camera measurements are preprocessed, giving an intermediate representation that solves part of the problem. These intermediate obtained values are then used as noisy inputs for the X estimator. In the second group, called direct, the systems skip the preprocessing step and directly use the measurements obtained from the observations as noisy inputs for the X estimator in a probabilistic model. This classification also determines how optimization is carried out, so direct methods optimize photometric errors (pixel intensity difference) since direct methods obtain photometric measurements. In contrast, indirect methods optimize geometric errors since preprocessing computes geometric values.

### 6.1 Classic + indirect methods

**Fig. 6:**
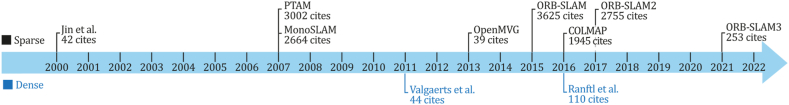
Most representative monocular SLAM, VO, or SFM classic indirect systems--A Timeline.

As previously mentioned, indirect methods use preprocessing steps to extract information about the input image sequence through visual features, descriptors, or optical flow. Although, at the same time, classic indirect systems can be designed to recover a sparse or dense 3D reconstruction environment, both subcategories will be discussed in sections 6.1, 6.1.1.2. Fig. 6 presents a timeline for the appearance of the most important methods of this category selected based on their number of citations and contributions for new implementations or comparisons. Fig. 6 shows that one of the most important SLAM developments belongs to this category, the ORB-SLAM system and its successors, which has one of this study's most impressive citation scores.

#### 6.1.1 Classic + sparse + indirect methods

This category includes some of the most popular approaches, typically based on 3D geometry estimation from keypoint matches using geometry error without prior geometry. This category of SLAM methods uses a subset of the pixel information to perform its constitutive processes, so the output is also a subset of the expected scene reconstruction, which in some applications, such as robotics, is sufficient to perform tasks such as robot navigation or even place recognition. Therefore, these methods are indirect due to their pre-processing, where new variables, such as features and their positions, are computed by replacing pixel information so that the rest of the processes are performed using these new values.

**Fig. 7:**
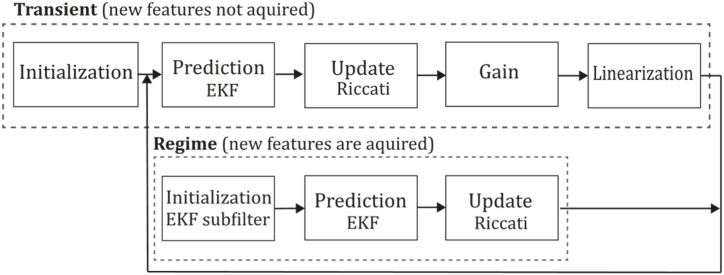
Diagram of Jin et al., algorithm. Adapted from Ref. [58].

**Jin et al. (2000).** One of the first works found in this category was developed in 2000 by Jin et al. [58], called "Real-Time 3-D Motion and Structure of Point Features" consisting of a system able to select and track a certain number of high-contrast point features in an image sequence estimating a three-dimensional relative position and motion from an inertial reference. The goal was achieved by selecting an N-tuple of points, a reference plane $Y_{0}$ and their depth using the projection ρrays, obtaining a discrete-time non-linear dynamic system through a translation vector function T a rotation matrix R and its linear and rotational velocities V, $\hat{\omega }$ using the hat notation, α represents corresponding accelerations. Authors assume that the noisy projection can be measured by $Y^{i}\left(t\right)=\pi \left(R\left(t\right)Y_{0}^{i}\rho^{i}+T\left(t\right)\right)+n^{i}\left(t\right)\in R^{2}$ corresponding to the projection model. Also, it was proven that the model was minimal, and the Extended Kalman Filter based on it was stable. Its conventional state space representation is as follows:

(1) $$\left\{ \begin{array}{c} Y_{0}^{i}\left(t+1\right)=Y_{0}^{i}\left(t\right),\text{with}\;i\in \left\{4,\ldots ,N\right\},Y_{0}^{i}\left(0\right)=Y_{0}^{i}\text{,}\\ \rho^{i}\left(t+1\right)=\rho^{i}\left(t\right)\;i\in \left\{2,\ldots ,N\right\},\rho^{i}\left(0\right)=\rho_{0}^{i}\\ T\left(t+1\right)=\exp\left(\hat{\omega }\left(t\right)\right)T\left(t\right)+V\left(t\right),T\left(0\right)=T_{0}\\ \Omega \left(t+1\right)=Log_{SO\left(3\right)}\left(\exp\left(\hat{\omega }\left(t\right)\right)\exp\left(\hat{\Omega }\left(t\right)\right)\right),\Omega \left(0\right)=\Omega_{0}\\ V\left(t+1\right)=V\left(t\right)+\alpha_{v}\left(t\right),V\left(0\right)=V_{0}\\ \omega \left(t+1\right)=\omega \left(t\right)+\alpha_{\omega }\left(t\right),\omega \left(0\right)=\omega_{0}\\ Y^{i}\left(t\right)=\pi \left(\exp\left(\hat{\Omega }\left(t\right)\right)Y_{0}^{i}\left(t\right)p^{i}\left(t\right)+T\left(t\right)\right)+n^{i}\left(t\right), \end{array}\right.$$

where $n^{i}∼N\left(0,\Sigma_{n}\right)$, $Log_{SO\left(3\right)}\left(R\right)$ stands for Ω and i={1...N}. The implemented algorithm uses the above model and performs initialization (initial feature selection), transient (prediction, update, gain, and linearization), and regime tasks (initialization, prediction, and update), where features are tracked, removed, and updated following a Riccati equation and new features are inserted to the state after a probation time. Fig. 7 presents the Jin et al. algorithm inspired by the article [58].

**Fig. 8:**
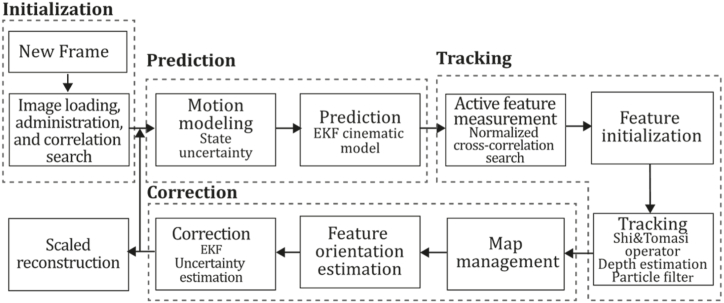
Diagram of MonoSLAM algorithm diagram. Adapted from Ref. [28].

**MonoSLAM (2007).** The first monocular visual SLAM system was introduced in 2007 by Davison et al. [28], built based on [[67], [68], [69]] to create a "pure vision" system able to construct persistent 3D maps of the environment on the fly while estimating camera ego-motion and closing loops to correct drift, in real-time by using a monocular camera as the only information source. This persistent sparse map is estimated within a probabilistic framework employing a set of landmarks. Key contributions in this work are the active guided measurement and high-quality mapping features, a motion model integration for smooth camera motion estimation to allow the system to capture prior information in an image sequence, and the implementation of feature initialization and feature orientation estimation techniques. Authors established that using SLAM, where probabilistic camera state estimation is performed simultaneously with its map, instead of SFM, is used to benefit in running estimates for efficient processing. The basis of the MonoSLAM approach is the estimation of a probabilistic feature map that represents the state of the camera at any time and all the features of interest, as the Extended Kalman Filter constantly updates this map. MonoSLAM uses large image patches (11×11 pixels) as long-term landmarks where detection is performed by Shi and Tomasi operators [70]. Due to the scale ambiguity of monocular SLAM, the system initialization was carried out by giving the system a certain amount of scene-prior information by placing a known rectangular target in front of the camera. Next, depth estimation was done by placing a semi-infinite line with its direction at each 2D position. Then, a set of discrete depth hypotheses were distributed along this line in the form of probability density functions so they are incrementally estimated during camera movement until distribution collapses to a peak; so when standard deviation drops below a threshold, distribution can be approximated as Gaussian. A drawback is that the algorithm complexity increases proportionally to the scene size, returning a sparse map of landmarks, which is far from an accurate reconstruction. Fig. 8 presents the MonoSLAM algorithm inspired by the article [28].

**Fig. 9:**
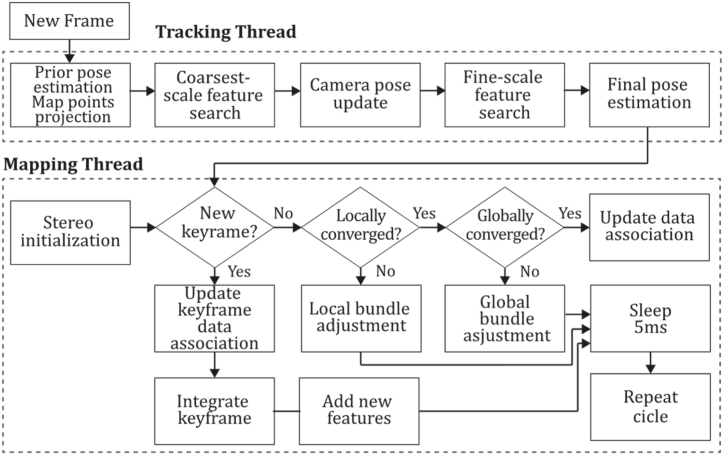
Diagram of PTAM algorithm. Adapted from Ref. [71].

**PTAM (2009).** In 2009, Klein & Murray [71] developed a tracking and mapping system called "Parallel Tracking and Mapping" (PTAM) applied to the augmented reality context, intended to transform a flat surface into a playing field that can be used for VR simulation, however, limited to small AR applications. PTAM was the first system performing tracking and mapping tasks in two parallel threads since modern computers have more than one processing core, where the tracking thread oversaw estimating a prior pose, projecting map points into an image, searching coarsest-scale features in the image, updating camera pose from these matches, patch searching of reprojected points, pose estimation of the current frame from the matches found. In contrast, the mapping thread was designed to perform map initialization, refinement, and expansion as keyframes are picked from the tracking thread, Levenberg-Marquardt bundle adjustment to adjust the pose for every keyframe and data association refinement using an outlier management technique based on a Tukey estimator. In this system, tracking and mapping are not linked, so many redundant frames could be skipped, allowing the processors to concentrate on a small number of keyframes, allowing operation with larger map size, and replacing incremental mapping with an accurate batch method, in this case, bundle adjustment. This application was developed to perform video game AR integrations and was compared to EKF-SLAM [29], reducing the trajectory error significantly. Fig. 9 illustrates the PTAM algorithm inspired by the article [71].

**OpenMVG (2013).** OpenMVG (Open Multiple View Geometry) Structure from Motion (SfM) system is an open-source project used for reconstructing 3D shapes of objects, scenes, or structures from a series of 2-dimensional images that can be acquired at the same time (using stereo setups), or from series of monocular images (incremental). It was proposed by Moulon et al. in 2013 [72] and improved upon prior methods from additional contributors by utilizing a hash-map algorithm, incorporating a graph-based geometry verification process, and scaling to handle large datasets [73]. The system uses a pipeline that involves camera calibration, feature extraction and matching, geometric filtering, 3D reconstruction, global optimization, and colourization. One of the main improvements of OpenMVG over previous SfM systems is the use of a hash-map algorithm, which optimizes the feature extraction and matching process by storing the features' positions and descriptors in memory for easy access and comparison, which allows an efficient management of large datasets and faster computation times. Also, OpenMVG employs a graph-based geometry verification process, which compares the estimated camera poses and 3D reconstructions with a similarity graph. The graph represents recurring geometric relationships and helps to eliminate false matches and outliers, improving the overall accuracy of the reconstruction. Another improvement of OpenMVG is the ability to handle large datasets and scalability. The system has been shown to handle up to 100,000 images in a single run and to reconstruct complex scenes and structures. This scalability is due to efficient memory management and data structure optimisations, such as the use of locally homogeneous patches and sparsity-based representation of feature matches. Several factors influence the quality of the reconstruction, including the camera calibration, the quality and number of images, the scalability of the system, and the accuracy of the feature extraction and matching. OpenMVG addresses these factors with features such as automatic camera calibration, parallelization of the feature extraction and matching, and robust estimation of camera poses and 3D points. The system also allows the user to fine-tune the reconstruction process and provides various visualization tools for examining and manipulating the 3D models.

**Fig. 10:**
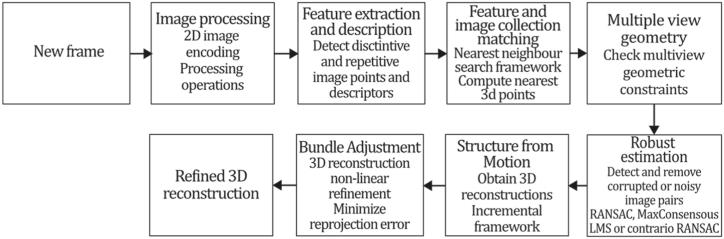
Diagram of OpenMVG algorithm. Adapted from the article [73].

The OpenMVG library was built upon a collection of modules displayed on a user interface that allows easy configuration. The modules are image processing, feature extraction and description, feature and image collection matching, multiple view geometry, robust estimation, structure from motion, and localization. The image processing module encodes each image in a 2D-pixel container based on the Eigen matrix structure, and it allows the image processing operations to be performed: image sampling, primitive drawing, colour space conversion, and image filtering. Feature extraction and description module oversees detecting distinctive and repetitive image points and descriptors, which allows OpenMVG to describe the image as a collection of regions which are abstract chosen attributes embedded in each point descriptor, which can be blob regions, corner regions or affine invariant regions. Feature and image collection matching are carried out in OpenMVG by providing a search framework based on the nearest-neighbours techniques for any vector dimension, which can be configured as BruteForce, ANN-kD trees, and Cascade hashing, which can be used to compute the nearest 3D points by matching features over the image sequence. The Multi-View Geometry module allows the checking of multi-view geometric constraints on matched pairs, so OpenMVG provides: multiple models and solvers like the 4-point algorithm for homography; the 7/8-point algorithm for fundamental matrix; the 5-point algorithm for essential matrix estimation; P3P, DLT, and ePnP for the absolute pose; similarity transformations; and triangulation tools. The robust estimation module detects and removes corrupted or noisy image pairs, including RANSAC and MaxConsensus for prior thresholding and Least Median of Squares and contrario-RANSAC as threshold-free techniques. Finally, the SFM module allows OpenMVG to obtain 3D reconstructions applying the global or incremental pipelines using images with low cross-coverage, where it is well identified that the incremental approach tends to suffer from drift due to its sequential nature. Finally, Bundle Adjustment is executed as part of the SFM module to perform a non-linear refinement over the SFM scene by minimizing the reprojection error. Fig. 10 illustrates the OpenMVG algorithm inspired by the article [73].

**ORB-SLAM (2015).** A couple of years later, an impressive new monocular system called ORB-SLAM by Mur-Artal et al. [11] was launched based on the use of ORB features, which are multiscale FAST corners with a 256-bit descriptor, the same used for tracking, mapping, relocalization, and loop closing, due to its fast computing and matching properties. The system starts with feature extraction, a preprocessing step that is why ORB-SLAM could be classified as an indirect technique. Here, the input is preprocessed to extract ORB features at salient key-point locations, and then the rest of the process is performed over these features, so the rest of the input image information is discarded. Compared to PTAM, ORB-SLAM performs bundle adjustment, which is considered the gold standard for SFM due to its excellent performance. Bundle Adjustment used to be considered unaffordable for real-time applications due to its high computational cost. However, authors thought it could be minimized by working on corresponding scene feature observations using a subset of keyframes, avoiding redundancy on keyframe selection, and using sets of keyframes with significant parallax and plenty of matches in loop closure. Thus, ORB-SLAM uses an initial estimation for keyframe poses and point location in the optimization stage, implementing optimization focused on local map exploration, providing the ability to perform global optimizations to close loops.

In this method, Bundle Adjustment optimization is performed for the 3-D locations $x_{\omega ,j}={\left(x_{\omega ,j},y_{\omega ,j},z_{\omega ,j}\right)}^{T}$, and the keyframe poses $T_{i\omega }$, are optimized by minimizing the reprojection error for the $x_{i,j}$ points, so the error for a point j in an i keyframe is:

(2) $$e_{i,j}=x_{i,j}-\pi_{i}\left(T_{i\omega },x_{\omega j}\right),\pi_{i}\left(T_{i\omega },X_{\omega ,j}\right)=\left[\begin{array}{c} f_{i,u}\frac{x_{i,j}\;}{z_{i,j}}+c_{i,u}\;\\ f_{i,v}\frac{y_{i,j}\;}{z_{i,j}}+c_{i,u} \end{array}\right],\text{and}\;{\left[\begin{array}{ccc} x_{i,j} & y_{i,j} & z_{i,j} \end{array}\right]}^{T}=R_{i\omega }x_{\omega ,j}+t_{i\omega }\text{,}$$

where $\pi_{i}$ is the projection function, $R_{i\omega }$ and $t_{i\omega }$ are rotation and translation components of $T_{i\omega }$. $\left(f_{i,u},f_{i,v}\right)$ and $\left(c_{i,u},c_{i,v}\right)$ are the focal length and main point associated with the i camera. This way, the cost function to be minimized is: $C=\sum_{i,j}H_{h}\left(e_{i,j}^{T},\Omega_{i,j}^{-1}e_{i,j}\right)$, where $H_{h}$ is the Huber robust cost function, $\Omega_{i,j}=\sigma_{i,j}^{2}{Ι}_{2\times 2}$ is the covariance matrix associated with the scale for each detected keypoint.

**Fig. 11:**
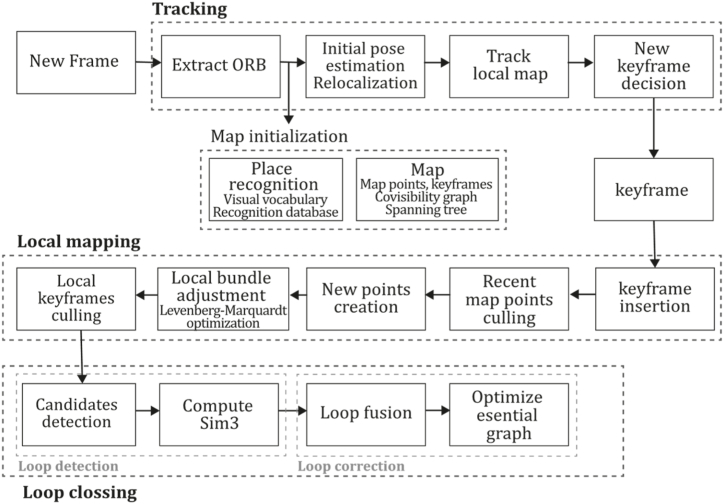
Diagram of ORB-SLAM algorithm. Adapted from Ref. [11].

ORB-SLAM is a system built over the systems: PTAM [71], a place recognition technique called Bags of Words of [74], the scale aware loop closing technique of [13], and the incorporation of co-visibility information studied in Refs. [75,76]. All these techniques combined and improved allowed the creation of a new system whose contributions include the use of the same tracking, mapping, relocalization, and loop closing features in real-time operation and satisfactory invariance to changes in viewpoint and illumination; use of a local co-visibility graph for tracking and mapping providing map size independence allowing real-time operation in large sceneries; use of a loop closing technique based on pose graph optimization; the ability to recover from tracking failure; a model selection based automatic initialization; and the implementation of map point and keyframe selection by the survival of the fittest technique. Similarly, PTAM ORB-SLAM uses three parallel threads for tracking, local mapping, and loop closing where the tracking thread performs the task of localizing the camera with every frame and decides when a keyframe should be inserted; the local mapping thread processes each new keyframe and executes bundle adjustment for 3D reconstruction of elements near to camera pose; and loop closing thread in charge of searching loops with each new keyframe and computing a similarity transformation measuring accumulated drift when a loop is detected. ORB-SLAM also implemented a non-prohibitive policy for spawning and culling keyframes for flexible map expansion, which can detect and delete redundant keyframes. Results showed that the main benefit of indirect techniques is that they can match features even with a wide baseline. According to Ref. [11], accuracy can still be improved by including points at infinity in the tracking containing primary rotation information. Also, it can be upgraded by using a denser map, or the system could be used as a backbone from which an accurate dense map may be built. Fig. 11 presents the ORB-SLAM algorithm inspired by the article [11].

**COLMAP (2016).** COLMAP is a method proposed for photogrammetry and computer vision, which aims to reconstruct a 3D scene from a set of 2D images. The main strategy of the method is to estimate camera poses and 3D points in the scene by optimizing a bundle adjustment problem. COLMAP employs feature-based matching to establish correspondences among images and then refines the matches based on geometric consistency to reject outliers. The method also introduces several improvements in the optimization process, such as a novel parameterization for rotation and a more robust optimization technique based on the Levenberg-Marquardt algorithm. SFM techniques are typically built over two stages: correspondence search and incremental reconstruction. The correspondence search is responsible for identifying overlaps in the input images and projections of the same points in such overlapping images to build a graph of image projections for each point. For this purpose, COLMAP performs feature extraction to select sets of local features invariant to radiometric and geometric changes to allow SFM to recognize them across multiple images; then, matching is executed to discover images viewing the same scene part by searching feature correspondences that are the most similar features in each image; finally, a geometric verification step must be addressed because matching is only based in appearance, and it is not guaranteed that feature correspondences are mapping the same scene point, so SFM verifies this matches by estimating transformations and if a transformation maps a sufficient number of features it is considered as verified. The second stage in COLMAP is incremental reconstruction, which takes the scene graph from the correspondence search to recover pose estimates and the scene structure as a point cloud. The incremental reconstruction starts with the initialization process that carefully selects an initial two-view reconstruction, which is crucial to prevent trajectory loss issues. Then, the image registration allows the system to register new images by solving the Perspective-n-Point (PnP) problem using the previously identified feature correspondences of triangulated points; then, the triangulation allows the extension of the scene point cloud representation by adding a set of points coming from each new image. Finally, since SFM tends to drift quickly to non-recoverable states, BA is applied to refine the camera and point parameters by minimizing the reprojection error. The main contributions of COLMAP to the SFM pipeline are incorporating improvements in image registration, triangulation, and BA procedures. COLMAP introduces a robust next-best image selection method to improve pose estimation and recover a reliable triangulation, which is an uncertainty-driven approach that uses a multi-resolution analysis by using a score S, which is higher if more points are visible and if the distribution of those points is uniform. In addition, COLMAP introduces a novel, robust, efficient triangulation procedure that aims to be more robust to outliers and recover independent points merged into one track by using the RANSAC approach to handle different levels of outlier contamination in the multi-view triangulation process. Finally, in COLMAP, BA was implemented for image registration and triangulation, allowing a local BA to be performed on the set of connected images for each image registration, and it performs a global BA each time the model grows a certain percentage.

**Fig. 12:**
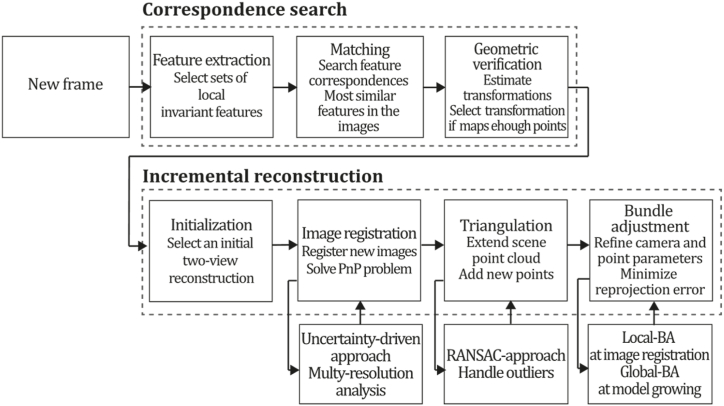
Diagram of COLMAP algorithm. Adapted from Ref. [77].

Compared to similar methods, COLMAP shows better performance in terms of accuracy, efficiency and scalability. For example, COLMAP can handle large image collections with hundreds of thousands of images while maintaining reconstruction quality without the limitations of handling large datasets. Moreover, COLMAP shows superior accuracy in reconstructing the scene's geometry, which is important for virtual and augmented reality applications. Another advantage of COLMAP is its flexibility in handling different types of input data, such as unordered image sets, image sequences, and video frames. These features make COLMAP suitable for various applications, from 3D modelling of cultural heritage to robotic vision and autonomous driving. Additionally, COLMAP provides a comprehensive set of tools for visualizing and analyzing the reconstruction results, including point cloud rendering, texture mapping, and error analysis. Fig. 12 presents the COLMAP algorithm inspired by the article [77].

**ORB-SLAM2 (2017).** Next, Mur. Artal and Tardós continued the work of ORB-SLAM, developing ORB-SLAM2 [38], a system that extended the capabilities of its predecessor, allowing the system to operate with monocular, stereo, and RGB-D sensors. It was mainly developed based on the previous system but included stereo matching procedures for stereo cameras and stereo coordinate generation for RGB-D sensors. The main innovation in the monocular scenario was implementing a fourth thread responsible for performing a full Bundle Adjustment after loop closure pose graph optimization to compute the optimal structure and motion solution. This optimization procedure implies high computational cost because it is performed over all the points and features, so it is executed in a separate thread, allowing the system to continue expanding the map and detecting loops simultaneously. Like ORB-SLAM, this system incorporated an embedded DBoW2 place recognition module for relocalization and a co-visibility graph for large environments. For stereo and RGB-D implementation, monocular key points are also used to contribute rotation and translation estimation but do not provide scale information because this prior information can be measured or triangulated by the sensor. This method uses Levenberg-Marquard optimization by the implemented module g2o, optimizing the camera pose in the tracking thread, the local window of keyframes, points in the mapping thread, and all keyframes and points after loop closure. Therefore, full Bundle Adjustment is the case of local Bundle Adjustment, where all map points and keyframes are optimized except the origin keyframe.

**Fig. 13:**
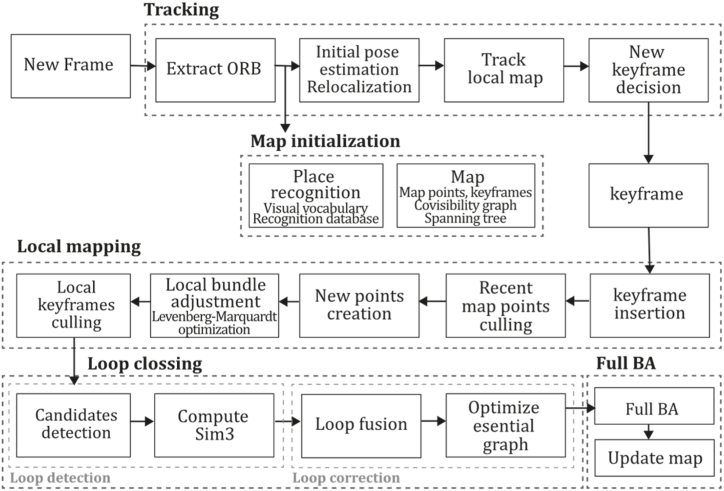
Diagram of ORB-SLAM2 algorithm. Adapted from Ref. [38].

ORB-SLAM2 also includes a localization mode that disables local mapping and loop closing threads for known areas as long as there are no significant changes in the landscape, allowing for long-term and lightweight localization and functionality. This method was compared with many of the existent Stereo, RGB-D, and monocular systems of the time, outperforming most of them in the EuRoC [78], TUM [79], and KITTI [7] datasets, proving its functionality in a large variety of environments. In addition, this study proposes that its application could be extended to a wide variety of new sensors like omnidirectional, fisheye cameras, and large-scale dense fusion. Fig. 13 illustrates the ORB-SLAM2 algorithm inspired by the article [38].

**Fig. 14:**
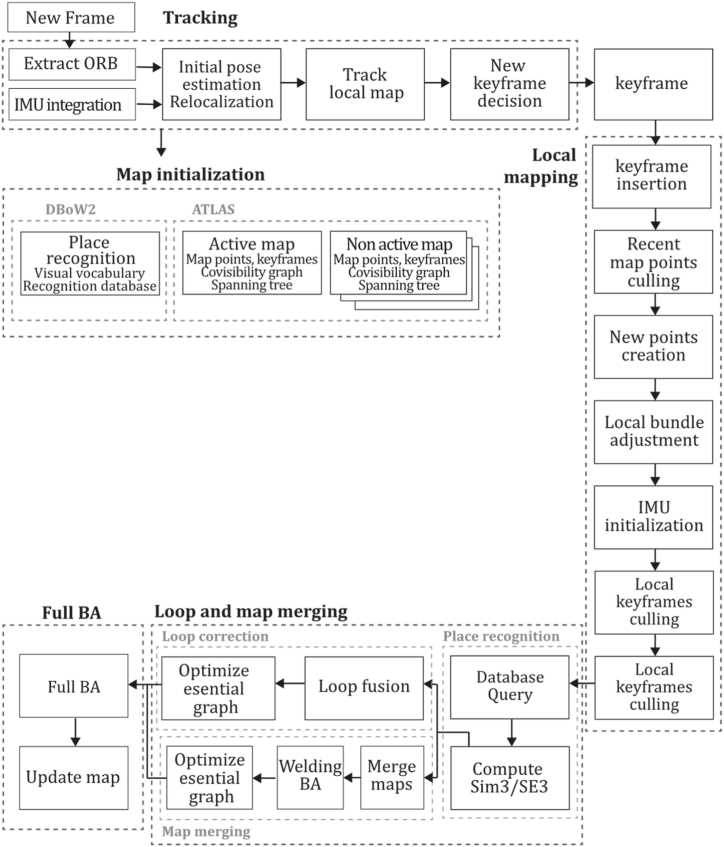
Diagram of ORB-SLAM3 algorithm. Adapted from Ref. [81].

**ORB-SLAM3 (2021).** It is well known that ORB-SLAM has been the gold standard of feature-based monocular SLAM systems for many years and has inspired the development of many other systems like DF-ORB-SLAM, ORB-SLAM-VI and ORB-SLAM3 [[80], [81], [82]]. ORB-SLAM3, proposed by Campos et al. [81], is the latest proposal to integrate and improve the developments of its predecessors. This system can perform visual, visual-inertial, and multimap SLAM with monocular, stereo, and RGB-D cameras for pinhole and fisheye devices. One of the most considerable contributions made in this work is its capability of performing four types of data association: short-term, enabling the system to match map elements of the last few seconds; mid-term data association, to match map elements close to the camera that present slight accumulated drift; long-term data association, to match map elements of previously visited areas using a place recognition technique regardless of accumulated drift; finally, multimap data association, to match and use BA map elements from previous mapping sessions creating a map from where the system can perform an accurate localization. The main contributions of this method compared to previous versions are: a monocular and stereo visual-inertial system based on Maximum-a-Posteriori estimation (MAP), improving ORB-VI using the initialization technique of [83]; a new place recognition technique to improve its recall by checking geometrical and local keyframes consistency with three co-visible map keyframes, slightly incrementing computational cost; ORB-SLAM atlas, which is a multimap SLAM system, inspired in Ref. [84], to represent a set of disconnected maps that can be used in every map operation of place recognition, camera localization, loop closure, and map merging, enabling the combination of maps built at different times performing an incremental multi-session SLAM or even creating new maps when tracking is lost; an abstract camera representation, bringing the capability of use to any camera model by providing its projection and un-projection Jacobian functions. Moreover, a significant improvement is the ATLAS system, providing an active map where the tracking thread localizes incoming frames, continuously optimizing and increasing the Atlas with new keyframes, where even non-active maps await to be connected. The tracking thread computes the pose of a current frame concerning the active map, minimizes reprojection error, and selects which frames become keyframes. The local mapping thread adds keyframes and points to the active map, removes redundant points, and refines the map by bundle adjustment using a window of neighbouring keyframes. Finally, the loop and map merging thread detects common regions in the Atlas, so if common areas are found, loop correction is performed, and then an independent thread performs full BA to refine the whole map. ORB-SLAM3 was tested against a large set of monocular, stereo, monocular inertial, and stereo inertial methods in the EuRoC and TUM-VI datasets using RMSE (Root Mean Square Error) and ATE (Absolute Trajectory Error) metrics where the system proved to outperform all the methods in most sequences. Still, it was reported that ORB-SLAM3 fails in texture-less environments, slow motion, or pure rotational applications. Fig. 14 illustrates the ORB-SLAM3 algorithm inspired by the article [81].

#### 6.1.2 Classic + dense + indirect methods

This formulation mainly estimates the 3D geometry from or in conjunction with the regularized optical flow field; combining the geometric error (deviation from the flow field) with the obtained geometric prior (explained by the smoothness of the flow field) [24] is common. As mentioned above, this category of monocular methods requires a large amount of input information using most pixel values to perform its constituting processes. For instance, dense monocular methods do not require extracting a subset of features because they work directly over the entire input. So, these methods do not require discrete features but involve major computational costs due to the larger amount of data that will be processed. This category of methods is considered indirect because most depend on optical flow information obtained in a preprocessing step.

**Valgaerts et al. (2011).** In 2012, Valgaerts et al. [85] performed a comparative study of dense and sparse methods, where the authors proposed a variational dense 3D reconstruction model to recover the fundamental matrix and optical flow by minimizing a single energy function. The authors also explored the difference between sparse feature-based methods for estimating epipolar geometry and dense energy-based methods commonly used for estimating correspondences within the image sequence. The investigation aimed to demonstrate that dense optical flow methods can also be used to estimate epipolar geometry, suggesting exploring a joint variational approach to jointly estimating epipolar geometry and optical flow. They compared feature-based techniques systems using Scale Invariant Feature Transform (SIFT) and Kanade-Lucas-Tomasi tracker (KLT) feature matching algorithms. Moreover, the use of Random Sampling Consensus (RANSAC) and Least Median of Squares (LMedS) techniques for estimating the fundamental matrix were evaluated. RANSAC extensions LORANSAC (local optimization RANSAC) of [86] and DEGENSAC (degenerate configurations RANSAC) [87] were analyzed as well. Several tests determined that dense estimation applied to epipolar geometry presents advantages over sparse methods when features are not well localized or when a small number of out-of-plane correspondences must be included to overcome degeneracy problems.

**Fig. 15:**
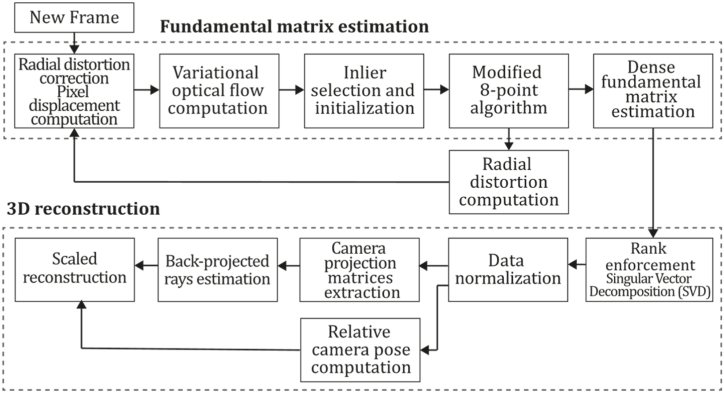
Diagram of Valgaerts et al., algorithm. Adapted from Ref. [85].

They also tested their variational model on many applications. One was automatic 3D reconstruction by extracting the camera projection matrices for the estimated basic matrix, triangulating the back-projected ray for each pixel, or applying a projective transformation when no additional camera or scene information is available. This method achieved 3D reconstruction simultaneously, solving dense epipolar geometry and two-image optical flow by associating a 3D point in space with each image pixel. Therefore, authors achieved higher accuracy and stability than separate epipolar and optical flow estimations. However, as this method relies on image sequence enabling stable estimation, the system was limited to rigid applications without moving objects. Fig. 15 illustrates Valgaerts et al. algorithm inspired by the article [85].

**Ranftl et al. (2016).** One of the most successful methods in this category is the work of Ranftl et al. [88], called "Dense Monocular Depth Estimation in Complex Dynamic Scenes", which is a system able to obtain a dense depth map for both static and dynamic objects using only two consecutive frames. It uses a segmentation algorithm that segments optical flow, obtaining a set of motion models, each with its epipolar geometry, and then the scene can be reconstructed, optimizing a convex problem. In this method, depth estimation comprises two stages. First is motion segmentation, where a dynamic scene is divided into a set of moving models with their epipolar geometry performed in an optical flow field formulated as a variational labelling problem. Second is reconstruction, where the scene is assembled by jointly reasoning the scale and location of different components relative to the camera by object triangulation and reconstruction of all its constitutive objects.

In the Motion segmentation stage, the dynamic scene is decomposed into an independent rigid motion set described by their fundamental matrix and a per-pixel assignment. This process, formulated as a joint estimation labelling problem, requires a dense optical flow field $F=\left(f_{x},f_{y}\right)$ between the $I_{1}$ and $I_{2}$ images generating a soft $u_{l}$ assignment of each pixel to either one l label for distinct $M_{l}$ motion models or an additional l+1 outlier label, so the formulation is:

(3) $$\left(u_{l}^{*},M_{l}^{*}\right)=\arg\;\min_{u_{l},F_{l}}\sum_{l=1}^{L+1}u_{l}\cdot g\left(F_{l}\right)+{\left\|W_{l}\nabla u_{l}\right\|}_{2,1},subject\;to\;\sum_{l=1}^{L+1}u_{l}^{i}=1,u_{l}^{i}\geq 0,\forall l.\;rank\left(F_{l}\right)=2\text{,}$$

(4) $$g^{i}\left(M_{l}\right)=d{\left(x_{1}^{i},M_{l}x_{2}^{i}\right)}^{2}+d{\left(x_{2}^{i},M_{l}^{T}x_{1}^{i}\right)}^{2}\text{,}$$

where $g^{i}\left(M_{l}\right)$ corresponds to the symmetric distance to the epipolar lines for every l∈{1...L} model, $x_{1}^{i}={\left[x^{i}y^{i},1\right]}^{T}$ and $x_{2}^{i}=\left[x^{i}-f_{x}^{i},y^{i}-f_{y}^{i},1\right]$ are the homogeneous coordinates in the first image and their corresponding homogeneous coordinates of the second image, respectively. ${\left\|W_{l}\nabla u_{l}\right\|}_{2,1}$ is the smoothness term, ∇ the linear operator represents the discrete difference between x and y, and $W_{l}$ is the diagonal weighting matrix applied to enable edge-preserving regularization. Then, the energy described in the equation is optimized using a variant of the primal-dual algorithm employing entropy proximal terms for implicitly representing simplex constraints to solve for labelling efficiently. So, fundamental matrices $M_{l}$ are decomposed in parallel for all the L models, and soft assignments $u^{i}$ are used to reweight the individual correspondence:

(5) $$M_{l}^{*}=\arg\;\min_{F_{l}}\sum_{i=1}^{M}u_{l}^{i}{\left({\left(x_{1}^{i}\right)}^{T}M_{l}\left(x_{2}^{i}\right)\right)}^{2},subject\;to\;rank\left(M_{l}\right)=2=2\text{,}$$

Then the subproblems are approximately solved using a reweighted version of an 8-point algorithm proposed in Ref. [89]. To obtain the number of dynamic models, the process performs the following steps: Apply the 8-point algorithm to mine a small set of candidates; Solve the energy equation by expanding the pool of motion candidates; add new models by robustly estimating motion from pixels with outlier label; expand the pool by splitting labels with disconnected regions; perform alternating minimization again and repeat the process until no further energy minimization can be made. Following this procedure, a set of epipolar geometries $F_{l}^{*}$ and membership probabilities $u_{l}^{*}$ is obtained for each pixel. Finally, a robust reconstruction is performed using these epipolar models and optical flow information using a super-pixel-based formulation.

**Fig. 16:**
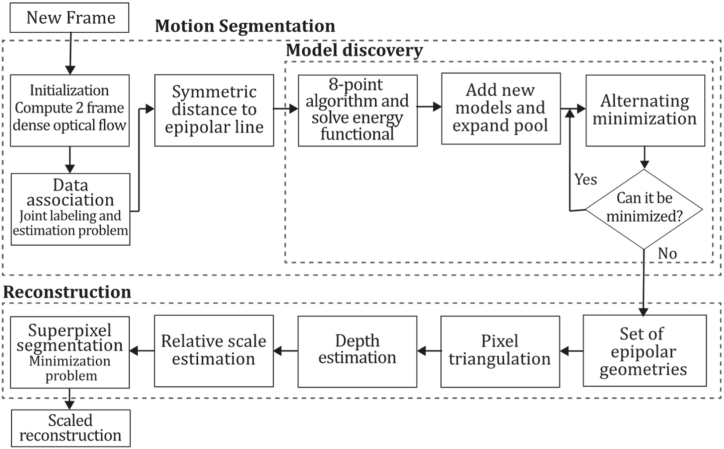
Diagram of Ranftl et al., algorithm. Adapted from Ref. [88].

This system was tested over KITTI [7] and MPI Sintel [90] datasets, outperforming most of the dynamic scene geometry from monocular video techniques of the time, using different techniques for optical flow computation like Large Displacement Optical Flow (LDOF), EpicFlow, and FlowFields. Thus, it was found that since the proposed method relies on optic flow information, the whole system will fail if the optic flow estimation fails. In addition, this process has the limitations of a purely geometric approach that does not use prior information about shape and scene size. Hence, the authors suggest that the proposed method can be complemented by ML techniques to estimate the absolute scale. Fig. 16 illustrates the Ranftl et al. algorithm, inspired by the article [88].

### 6.2 Classic + direct methods

Direct methods are developed to recover scene geometry and agent ego-motion using direct pixel intensity information, so unlike indirect methods, these formulations do not require pre-processing steps such as feature extraction (which drastically reduces the amount of information available to the SLAM system, saving computational resources and time, but limiting the final 3D reconstruction density). In contrast, a direct approach works with each pixel intensity value on the image or at least most of them, so the final 3D reconstruction quality is commonly higher than indirect approaches. However, direct formulations rely on the brightness constancy assumption, which establishes that brightness over an object's position must be the same over different angles. Unfortunately, this is not always true, so these formulations are known to fail in scenes that display motion blur, moving objects, or non-Lambertian surfaces.

**Fig. 17:**
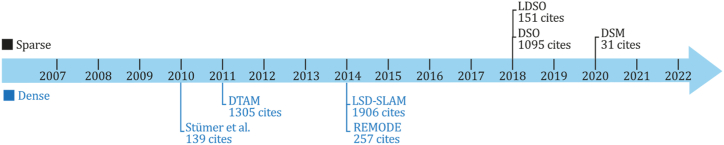
Timeline for the most representative monocular SLAM, VO, or SFM classic direct systems.

This category of methods can also be divided according to the final density of the 3D map so that there are dense and sparse formulations, which will be discussed in sections 6.2, 6.2.1.2. Fig. 17 shows a timeline of the most representative contributions in this area. As shown in Fig. 17, three of the most representative monocular systems of all time belong to the category DTAM [27], LSD-SLAM [37], and DSO [24]. It must be noted that although DSO has recently been introduced, it has attracted the interest of many researchers, achieving impressive citation scores.

#### 6.2.1 Classic + dense + direct methods

This category of slam approaches is dense because most input information is used to perform the reconstruction. In addition, they are classified as direct techniques because of the lack of preprocessing stages, so the input image is directly employed to obtain environment geometry. This formulation commonly applies photometric error and geometric priors to estimate dense or semi-dense geometry because they work directly on pixel intensity information.

**Stühmer et al. (2010).** Stümer, Gumhold, and Cremers [68] suggested one of the first approaches to these techniques [91], called "Real-Time Dense Geometry from a Handheld Camera," a real-time variational system to estimate dense depth maps using multiple images directly. Given a set of images, this method uses data terms relative to the coordinate system of a specific view and uses its perspective projection to map such coordinates in a second camera frame; thus [91], the following energy function was proposed to estimate depth maps using multiple images:

(6) $$E\left(h\right)=\lambda \int_{Y}\sum_{i\in T\left(x\right)}\left|\rho_{i}\left(x,D\right)\right|d^{2}x+\int_{Y}\left|\nabla D\right|d^{2}x\text{,}$$

where D is the depth map, $x={\left(x_{1},x_{2},1\right)}^{T}$ homogeneous 2D coordinates, d(x,D) is the depth value for each pixel, $Y_{i}$ is the image plane, and $T_{i}$ is the camera pose. In this case, T(x) contains all image indexes where perspective projection $\pi \left(\exp\left(\hat{T_{i}}\right)\cdot d\left(x,D\right)\right)$ is contained in the image boundaries, and $\rho_{i}\left(x,D\right)$ is the linearized residual data term for an $I_{i}$ image:

(7) $$\rho_{i}\left(x,D\right)=I_{i}\left(x,D_{0}\right)+\left(D-D_{0}\right)I_{i}^{D}\left(x\right)-I_{0}\left(x\right)\text{,}$$

where $I_{i}^{D}\left(x\right)$ represents the derivative $\frac{d}{dD}I_{i}{\left(x,D\right)|}_{D_{0}}$. As can be seen, the formulation takes direct pixel information into the energy function, and the D depth map is obtained after a minimization procedure, so this is a direct method. In addition, it is noticeable that this energy function is quite complex because the data term consists of the sum of absolute values for linear functions, and simple thresholding techniques cannot minimize it. Hence, the authors provide a proposal for generalized thresholding.

**Fig. 18:**
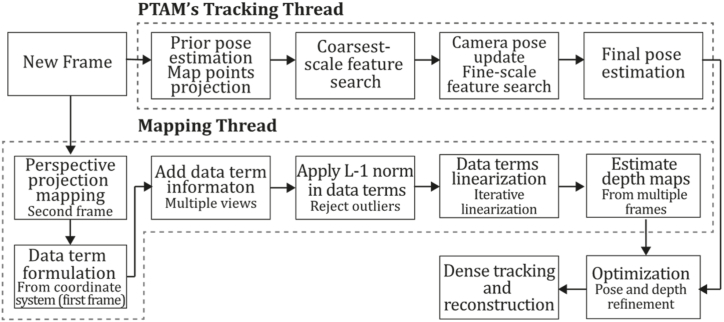
Diagram Stühmer et al. algorithm. Adapted from Ref. [91].

The advantage of this dense multi-view proposal over two-image techniques is that different views should help estimate disparity information in areas occluded by other views because this system can add information from images where the object is not occluded. Another benefit is an increase in the signal-to-noise ratio, which improves results when the input images are affected by noise, which is common in video captured by most webcams or handheld devices. To summarize, in this method, instead of using real-time pose estimation measurements, the depth map is estimated using the current input image and the N closest keyframes to the current pose, so by using keyframe camera pose estimates, the amount of noise is minimized. Finally, this proposal was embedded with the camera tracking module of a PTAM implementation [41], which can store keyframes, and each camera pose associated with each keyframe is refined iteratively. Similarly, the depth map related to its corresponding keyframe is refined using the N closest keyframes. Fig. 18 illustrates the Stühmer et al. algorithm inspired by the article [91].

**Fig. 19:**
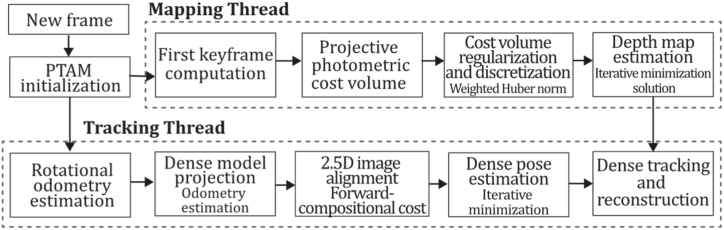
Diagram of DTAM algorithm. Adapted from Ref. [27].

**DTAM (2011).** Another direct dense approach was proposed by Newcombe et al. [27], a dense per-pixel method that aims to create a dense 3D model by performing a dense and sub-pixel reconstruction for accurate camera tracking. This dense model was used to perform a full image alignment with respect to the current model so that camera motion could be estimated. Immediately, the model is extended and updated using the tracked images by creating and refining dense depth maps. In this method, the dense model corresponds to overlapping keyframes, where depth values d are back projected from every pixel, resulting in a direct method. Here an r keyframe comprises an $I_{r}$ image, a camera pose $T_{r\omega }$, and a $C_{r}$ cost. Every $q_{r}$ pixel has an associated cost error $C_{r}\left(q,d\right)$ for each d depth value. Then a large amount of m∈T(r) video frames are used to compute the cost volume, being T(r) a set of nearby frames. Then, the photometric error is computed by projecting each point of the volume into all the overlapping images summing $L_{1}$ norms of each photometric error:

(8) $$C_{r}\left(q,d\right)=\frac{1}{T\left(r\right)}\sum_{m\in T\left(r\right)}{\left\|\rho_{r}\left(I_{m},q,d\right)\right\|}_{1}\text{,}$$

(9) $$\rho_{r}\left(I_{m},q,d\right)=I_{r}\left(q\right)-I_{m}(\Lambda \left(\left(\zeta T_{mr}\Lambda^{-1}\left(q,d\right)\right)\right)\text{,}$$

where $\rho_{r}$ is the photometric error for every overlapped image, ζ is the intrinsic matrix, $\Lambda \left(x_{c}\right)={\left(x/z,y/z\right)}^{T}$ is de-homogenization for a 3D point $x_{c}={\left(x,y,z\right)}^{T}$. The inverse depth map is obtained by minimizing the energy functional $E_{d^{*}}$, which comprises non-convex photometric error cost as a data term and a convex regularizer term:

(10) $$E_{D^{*}}=\int_{\Omega }\left\{g\left(q\right){\left\|\nabla d^{*}\left(q\right)\right\|}_{\epsilon }+\lambda C\left(q,d^{*}\left(q\right)\right)\right\}dq\text{,}$$

where $g\left(q\right)=e^{-\alpha {\left\|\nabla I_{r}\left(q\right)\right\|}_{2}^{\beta }}$ is per pixel weight, $D^{*}$ is the inverse depth map, $\nabla d^{*}\left(q\right)$ is the inverse depth map gradient, and Ω is the image domain, ϵ is set to a small value to reduce the stair-casing effect, λ=1/(1+0.5d) reflects data term quality and α and β are auxiliary variables. Thus, DTAM runs over an energy minimization framework using a photometric error data term and a robust spatial regularization term, so it starts defining a projective photometric cost volume (disparity space image in stereo matching) regularized using a weighted Huber norm on the inverse depth map gradient. Then, it is discretized and solved using duality principles to get the primal-dual form, where the weighted Huber regulator is replaced by its conjugate by applying the Legendre-Frenchel transform, so the inverse depth map can be extracted by iteratively minimizing the cost volume for every pixel from a reference frame. DTAM uses the PTAM point-feature-based method for initialization until the first keyframe is captured. It then switches to its own fully dense tracking and mapping pipeline. Next, a new keyframe is added when the number of pixels without visible surface information from the previous predicted image is below a threshold. In brief, this method estimates camera pose in real-time by finding motion parameters generating a synthetic view that best matches a live video frame. Fig. 19 illustrates the DTAM algorithm inspired by the article [27].

**REMODE (2014).** In 2014 Pizzoli, Förster, and Scaramuzza [4] proposed another dense direct method called REMODE, an abbreviation of "Regularized Monocular Depth Estimation", which is able to perform a dense depth map computation using Bayesian estimation and a novel complex optimization, developing a platform that acts like a depth sensor able to handle a large depth range. In REMODE, each pixel depth was estimated independently using a probabilistic approach and a new smoothing proposal. This per-pixel Bayesian depth estimation was built over the method proposed in Ref. [92], extending it by adding an optimization step consisting of regularization using a weighted Huber norm. However, unlike DTAM, depth uncertainty was used to exploit a convex formulation, avoiding the effect of noisy camera localizations. Thus, in REMODE, depth is computed as a Bayesian estimation problem by triangulating from a reference view and the last view acquired. Then, the pixel's depth is handled as a parametric model updated for every observation, and smoothness is achieved by minimizing a regularized energy function. Furthermore, REMODE includes a probabilistic approach where a depth hypothesis $d_{k}$ is generated using the $\left\{I_{k},T_{k,\omega }\right\}$ observation by triangulating r and k views, where $T_{k,\omega }$ is a rigid body transformation that describes the camera pose for every image. The depth sensor was modelled as a distribution that combines a good measurement, normally distributed around the real depth $\hat{d}$, and an outlier measure containing the depth of the desired structure.

(11) $$p\left(d_{k}|\hat{d},\rho \right)=\rho N\left(d_{k}|\hat{d},\sigma_{k}^{2}\right)+\left(1-\rho \right)U\left(d_{k}|d_{\min},d_{\max}\right)\text{,}$$

where ρ and $\sigma_{k}^{2}$ are the probability and variance of good measurement, $p\;\left(\hat{d},p\right)$ is the prior (knowledge of uncertainty) on true depth and the ratio of measurements supporting it. Then, the corresponding posterior is approximated as a product of Gaussian distribution for depth and Beta distribution for the inlier ratio.

(12) $$q\left(\hat{d},\rho |a_{k},b_{k},\mu_{k},\tau_{k}^{2}\right)=Beta\left(\rho |a_{k},b_{k}\right)N\left(\hat{d}|\mu_{k},\tau_{k}^{2}\right)\text{,}$$

$a_{k},b_{k}$ are parameters controlling Beta distribution. Then, for every q pixel $\mu_{k}$ and $\tau_{k}^{2}$ are the mean depth estimation, its confidence for each observation and the denoised depth map F(q) is obtained by the following energy minimization:

(13) $$\min_{F}\int_{\Omega }\left\{G\left(q\right){\left\|\nabla F\left(q\right)\right\|}_{\epsilon }+\lambda {\left\|F\left(q\right)-D\left(q\right)\right\|}_{1}\right\}dq\text{,}$$

**Fig. 20:**
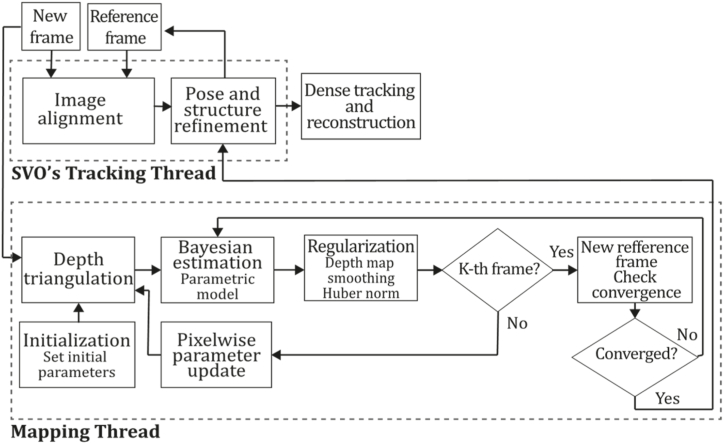
Diagram of REMODE algorithm. Adapted from Ref. [4].

D(q) is the depth map, and G(q) is the “G-Weighted Total Variation” weighting function [93]. These equations are basic methods and examples of how a probabilistic approach can be incorporated to obtain a denoised depth map directly from pixel information. REMODE uses a tracking thread inspired by the odometry system of SVO [23], using an image alignment formulation to estimate the pose but working only with pixel intensity information. After that, the mapping thread triangulates the depth using each frame and the reference view, so the depth of each pixel is formulated as a parametric model computed as a Bayesian estimation problem that includes a regularizer based on the gradient Huber norm of the gradient, so the solution is obtained iteratively by minimization, exploiting a primal-dual formulation and a gradient descent-ascent technique. Fig. 20 illustrates the REMODE algorithm inspired by the article [4].

**LSD-SLAM (2014).** In the study by Engel et al. [37], a real-time monocular SLAM and 3D reconstruction system was developed that not only tracks the camera motion locally but also constructs consistent, large-scale, environment-dense maps by using a semi-dense representation that tracks depth values only on gradient surfaces. The method uses direct image alignment and filter-based estimation of semi-dense depth maps based on the proposal [65]. The global depth map is represented as a pose graph consisting of keyframes as vertices and 3D similarity transforms as edges, with the ability to detect environmental scale changes and correct for accumulated drift. LSD-SLAM uses an appearance-only loop detection algorithm, FAB-MAP [94], to propose candidates for large loop closures and extract their features without reusing any information acquired in the visual odometry front end. Contributions of the LSD-SLAM method consist of a direct method to perform alignment of two keyframes on ξϵsim(3), and a probabilistically consistent incorporation of noisy uncertainty of the estimated depth into tracking. The novel image alignment is performed by Gauss-Newton minimization of the photometric error:

(14) $$E\left(T\right)=\sum_{i}{\left(I_{ref}\left(q_{i}\right)-I\left(\omega \left(q_{i},{D^{*}}_{ref}\left(q_{i}\right),T\right)\right)\right)}^{2}\text{,}$$

where I are images, $D^{*}$ is the per-pixel inverse depth map, $q_{i}$ is the image point, ω is a 3D projective warped function, and T is the camera pose. The complete method requires tracking, depth map estimation, and map initialization. The tracking module continuously tracks new images estimating the rigid body pose on the current keyframe, so the relative pose is calculated by minimizing the variance-normalized photometric error:

(15) $$\min_{T\in SE\left(3\right)}\sum_{p\in \Omega D_{i}}{\left\|\frac{r_{q}^{2}\left(q,T_{ij}\right)}{\sigma_{r_{q}}^{2}\left(q,T_{ij}\right)}\right\|}_{\delta }\text{,}$$

where $r_{q}^{2}$ and $\sigma_{r_{q}}^{2}$ are the photometric residual and variance, respectively. Also, for adding a keyframe to the map, the closest keyframes are found, and the edges are estimated by SE(3), so minimization is performed by the equation:

(16) $$\min_{T\in SE\left(3\right)}\sum_{q\in \Omega D_{i}^{*}}{\left\|\frac{r_{q}^{2}\left(q,T_{ij}\right)}{\sigma_{r_{q}}^{2}\left(q,T_{ij}\right)}+\frac{r_{d}^{2}\left(q,T_{ij}\right)}{\sigma_{r_{d}}^{2}\left(q,T_{ij}\right)}\right\|}_{\delta }\text{.}$$

**Fig. 21:**
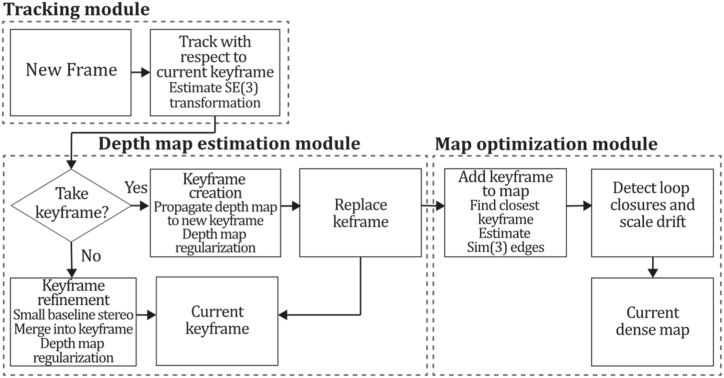
Diagram of LSD-SLAM algorithm. Adapted from Ref. [37].

Summarizing LSD-SLAM can obtain dense depth maps by estimating the rigid body pose from camera frames with respect to the current keyframe, using the previous frame for initialization (tracking). Next, the tracked frames are used to refine or replace the current keyframe so that depth is refined by multiple per-pixel small baseline comparisons (depth map estimation). Finally, when a new keyframe is replaced as a tracking reference and no further refinement is executed, it is added to the global map (map optimization). Fig. 21 shows the LSD-SLAM algorithm inspired by the article [37].

#### 6.2.2 Classic + sparse + direct methods

This category of formulations typically uses photometric error optimized directly from the input frames without requiring the introduction of geometric priors and preprocessing steps. A crucial benefit from the direct formulation is that it commonly uses pixel-wise inverse depth, which does not require a point to be recognized by itself, allowing a more grained and detailed geometry representation. Furthermore, this process allows for the sampling from every pixel, including edges and weak intensity variations, adding robustness to textured environments, whereas sparse methods do not require geometric priors, avoiding their limitations. Such priors involve the introduction of correlations between geometry parameters in which real-time statistically consistent joint optimization is typically impossible. These priors can also introduce bias, resulting in the tendency to reduce large-scale accuracy. Direct methods do not rely on a set of points' repeatability, so they can operate on low-texture surfaces with contours. Many methods from this category apply photometric bundle adjustment to minimize the photometric error of mapped point observations on a local sliding window of active keyframes, where points are sampled across pixels with high gradients, such as edges and intensity variations. In this context, VO systems typically use sliding windows that select close-in-time active keyframes, marginalizing map points far from the field of view. This situation could represent a disadvantage since VO systems cannot benefit from map point reobservations. On the other hand, VSLAM sparse direct systems typically build persistent maps of the scene representing a network of keyframes connected through observing the same region at different times.

**DSO (2017).** The work of [24] introduced a sparse formulation of direct visual odometry called Direct Sparse Odometry (DSO). This approach was designed to combine the advantages of direct methods (such as the ability to reconstruct most points, not just corners) with those of the sparse approach (such as the flexibility and efficiency of joint optimization). This monocular visual odometry algorithm can track even scenes with little texture, where indirect approaches often fail. DSO performs a continuous optimization of the photometric error applied to a set of recent frames, taking into account a photometrically calibrated model in the image formation process inspired by the proposal of [95]. At the same time, it optimizes the full likelihood for all the parameters involved in the model, such as camera poses, intrinsics, extrinsics, and inverse depth values in a photometric process equivalent to a windowed sparse bundle adjustment. Such processes require enough accurate initializations in the front end to carry out a non-convex optimization in the back end.

The minimization process has to be carried out for the photometric error of a point p in a reference frame $I_{i}$ over a small pixel neighbourhood, where experimentally, it was revealed that 8 pixels arranged in a residual spread pattern provide sufficient information for computation. The formulation for photometric error to be minimized is as follows:

(17) $$E_{qj}:=\sum_{i\in N_{q}}\omega_{q}{\left\|\left(I_{j}\left[q^{\prime }\right]-b_{j}\right)-\frac{t_{j}e^{aj}}{t_{i}e^{ai}}\left(I_{i}\left[q\right]-b_{i}\right)\right\|}_{\gamma }\text{,}$$

where $N_{q}$ is the set of pixels, $t_{i}$, and $t_{j}$ are exposure times for the $I_{i}$ and $I_{j}$ images, $a_{i}$, $b_{i}$, $a_{j}$, and $b_{j}$ are the brightness transfer function parameters for $T_{i}$, $T_{j}$ poses of the involved frames. The gradient-dependent weighting $\omega_{q}$ and the projected point position $q^{\prime }$, are given by:

(18) $$q^{\prime }=\Pi_{c}\left(R\Pi_{c}^{-1}\left(q,d_{q}\right)+t\right)\;\text{with}\;\left[\begin{array}{cc} R & t\\ 0 & 1 \end{array}\right]≔T_{j}T_{i}^{-1},\omega_{q}:=\frac{\zeta^{2}}{\zeta^{2}+{\left\|\nabla I_{i}\left(q\right)\right\|}_{2}^{2}}\text{,}$$

where ζ represents the camera intrinsics matrix, $\Pi_{\zeta }$, $\Pi_{\zeta }^{-1}$ are projection and the back-projection functions, and $d_{q}$ is the inverse depth for a projected point position. Finally, i runs for all F frames, q runs for all $P_{i}$ points of the image, and j runs over all obs(q) frames, where the point is visible, so the full photometric error for DSO was:

(19) $$E_{photo}=\sum_{i\in F}\sum_{q\in P_{i}}\sum_{j\in obs\left(q\right)}E_{qj}\text{.}$$

**Fig. 22:**
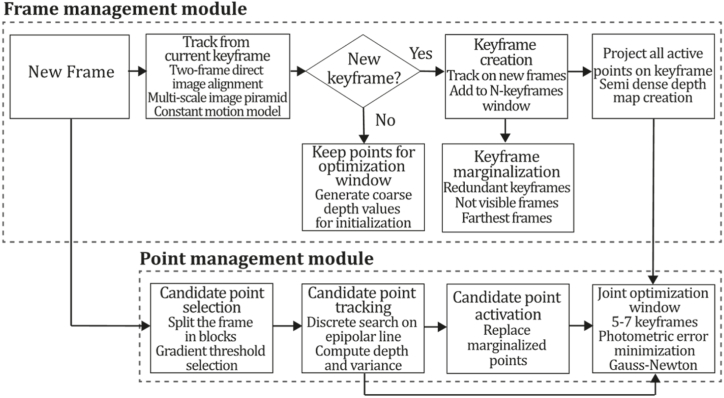
Diagram of DSO algorithm. Adapted from Ref. [24].

DSO algorithm performance comprises two modules in control of frame and point management. Frame management is intended to work over a set of active frames where each new frame is tracked from the current keyframe, two-frame direct alignment, a multiscale image pyramid, and a constant motion model track all its points. Then, a new keyframe is created when the field of view changes, on occlusions and disocclusions, and when camera exposure time changes. Consequently, old keyframes are marginalized when they are not visible enough, and the farthest keyframe is marginalized when the maximum number of active keyframes is exceeded. Finally, the point management module oversees the selection of candidate points within an optimization window, point tracking and candidate point activation as required for windowed optimization. The algorithm was evaluated adequately over three datasets, proving that using a large amount of data does not necessarily increase accuracy; however, using a sparse set of points improves accuracy and robustness. Fig. 22 introduces the DSO algorithm inspired by the article [24].

**LDSO (2018).** An extension of the DSO system was developed in the work of Gao et al. [64]. Since DSO allows using any pixel with a significant intensity gradient, it ensures repeatability points favouring corner features to detect loop closure candidates using the bag-of-words approach. Depth estimates of matched feature points were used to compute Sim (3) constraints; thus, in combination with pose-only bundle adjustment and point cloud alignment, they are fused with a co-visibility graph of relative poses extracted from the DSO sliding window optimization stage.

**Fig. 23:**
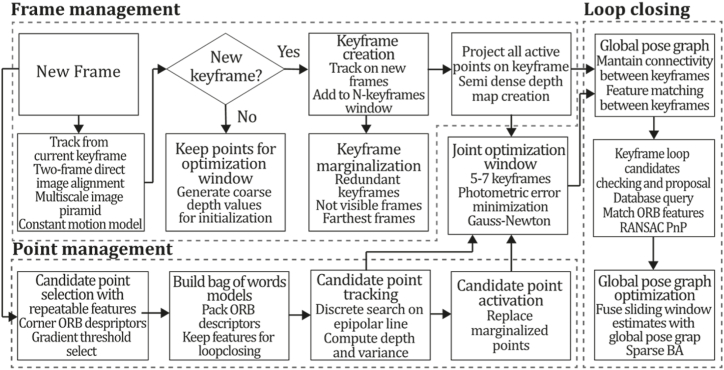
Diagram of LDSO algorithm. Adapted from [64].

Point selection is still needed in direct methods, yet a noticeable difference between direct and indirect methods is that point repeatability is not required in direct methods. In LDSO, corners and high gradient pixels are used where corners are used for generating BoW models, and the other pixels are used for tracking. LDSO proposes loop candidates for a keyframe by querying the database (using only those outside the window). Then, the method tries to match each corresponding feature, and the initial guess of SE (3) is computed by performing a RANSAC PnP. Next, a Sim (3) transformation is optimized using the Gauss-Newton method, minimizing the following cost function:

(20) $$E_{loop}=\sum_{m_{i}\in Q_{1}}\omega_{1}{\left\|S_{cr}\Pi^{-1}\left(q_{i},d_{q_{i}}^{*}\right)-\Pi^{-1}\left(m_{i},d_{q_{i}}^{*}\right)\right\|}_{2}+\sum_{m_{j}\in Q_{2}}\omega_{2}{\left\|\Pi \left(S_{cr}\Pi^{-1}\left(q_{j},d_{q_{j}}^{*}\right)\right)-m_{j}\right\|}_{2}\text{,}$$

where $S_{cr}$ is the loop candidate for the current keyframe, Π and $\Pi^{-1}$ are projection and back projection functions, respectively. $\omega_{1}$ and $\omega_{2}$ are weights to balance measurement units, $Q_{2}$ and $Q_{1}$ are ORB features with and without depth information, respectively. $q_{i}$ are the reconstructed features, $d_{q_{i}}^{*}$ are their inverse depths, and $m_{i}$ are the matched features for the current frame. By integrating loop closure and global map optimization, LDSO reduced rotation, translation, and scale drift while keeping tracking accuracy and robustness like DSO. One of the key contributions from LSDO was using ORB descriptors as corner trackers introduced to be packed into Bag of Words (BoW), especially contributing to performing feature matching between keyframes. In this way, the LDSO implementation is pretty like the original DSO; however, it adds a loop closing module based on a global pose graph optimization that works over 5 to 7 DSO keyframes sliding window, enabling the system to use even marginalized keyframes to close loops. This process is achieved by maintaining connectivity between keyframes and constantly matching ORB features between all keyframes and the current keyframe so loop candidates can be submitted by querying the keyframe database; then, LDSO performs global pose graph optimization fusing the sliding window estimates with the global pose graph. Fig. 23 presents the LDSO algorithm inspired by the article [64].

**Fig. 24:**
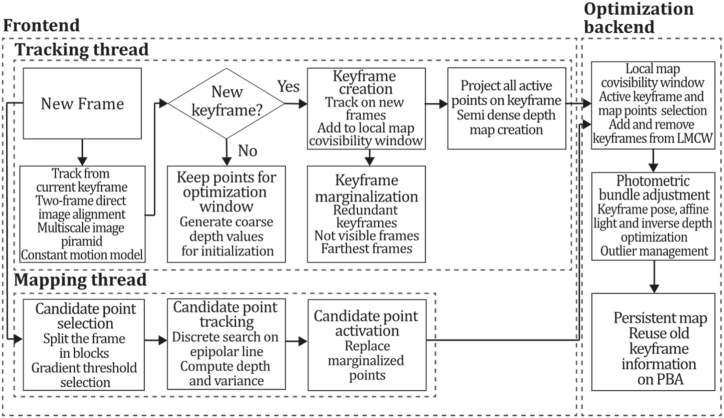
Diagram of DSM algorithm. Adapted from Ref. [30].

**DSM (2020).** Another notable recent work in this category is the Direct Sparse Mapping (DSM) proposed by Zubizarreta et al. [30], a system built over the DSO basis. However, as a VSLAM approach, the authors tried to use information from scene reobservations to improve accuracy, reduce motion drift and reduce structural inconsistencies. This method can be considered the first monocular visual SLAM system able to detect point reobservations from each entire image and extract the rich information that they provide. In contrast to LDSO [64], which uses only a sparse set of feature reobservations, DSM can build a persistent map, allowing the reuse of existing map information through a photometric formulation without incorporating a pose graph or relocalization. It uses a local map co-visibility window (LMCW) criteria to detect active keyframes that observe the same region, a coarse-to-fine strategy to process point reobservations with the photometric model, a robust influence function based on the t-distribution and a pixel-wise outlier management strategy to increase PBA consistency against outliers that could be generated as activation of distant keyframes occurs. Like many other monocular VSLAM methods, the system presents a tracking frontend and an optimization backend, where the tracking thread manages to obtain the camera pose, selecting frames that may turn into keyframes. Also, the optimization mapping thread processes every new frame to track points from active keyframes, which are the input for PBA optimizing motion and structure. In addition, this thread maintains global consistency by removing outliers, detecting occlusions, and avoiding point duplications. Robust nonlinear PBA was implemented using an outlier management strategy based on the photometric error distribution, where t-distribution proved to explain better dense photometric errors than other distributions through a weight function [96,97]. Experiments demonstrated that DSM outperformed similar sparse direct and indirect systems using a t-distribution robust influence function along with 4 or 3 co-visible keyframes for LMCW in most of the sequences of the EuRoC dataset. Fig. 24 illustrates the DSM algorithm inspired by the article [30].

### 6.3 Classic + hybrid methods

Only a few works can be properly catalogued as hybrid methods, considering direct vs. indirect classification. It is well known that the gold standard of this category is the Semi-Direct Monocular Visual Odometry (SVO) of [23], including its variants and implementations [98,99] which, despite being a few, have highly attracted the interest from researchers due to their ability to combine the advantages of direct and indirect formulations. For example, SVO only extracts features as a pre-processing stage when a keyframe is initialized; the system then performs direct motion estimation instead of feature matching. It thus benefits from both paradigms, achieving the high accuracy of direct methods and the speed of sparse indirect formulations.

**Fig. 25:**
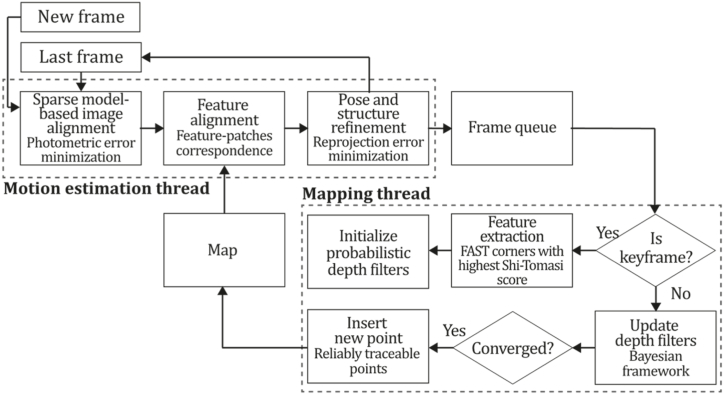
Diagram of SVO algorithm. Adapted from Ref. [23].

**SVO (2014).** The work "Fast Semi-Direct Monocular Visual Odometry" by Förster et al. [23] is an approach in which the authors aimed to develop a system that does not require feature extraction or robust matching to estimate ego-motion addressed by working directly on pixel intensities. SVO is known as a semi-direct method because it combines the advantages of both direct and indirect techniques as it is divided into two threads: motion and mapping using feature correspondences obtained by direct motion estimation instead of feature extraction or matching. This process reduces the feature extraction procedures, which are only required when a keyframe is chosen to insert new points on the map. The system also uses many small patches instead of a small set of large planar patches, which performed better, as reported in Refs. [100,101], increasing robustness and allowing the neglect of patch normals. The motion thread estimates camera motion using a sparse model-based image alignment algorithm, where sparse point-feature information is sufficient to estimate motion and feature correspondences, minimizing the photometric error between correspondences of the same 3D points. Next, the reprojected points are refined, and the pose and structure are refined by minimizing the reprojection error. The mapping thread uses a Bayesian filter to model outlier measurements to estimate the depth for each feature position, so 3D points are inserted when its depth filter uncertainty becomes small enough, resulting in a map of outliers and points to be efficiently tracked. One of the key strengths of SVO to be pointed out is its high framerate, able to run at 55 fps on embedded computers and at 300 fps on consumer laptops of the time, providing an outlier-resistant probabilistic mapping method and robustness to redundant and low-texture scenarios. Fig. 25 shows the SVO algorithm inspired by the article [23].

### 6.4 General comments for classic approaches

As mentioned above, monocular 3D reconstruction is an ill-posed problem that can be formulated, solved and optimized from different perspectives. This section has reviewed the most representative monocular SLAM, VO, and SFM approaches, which can be catalogued as classic because they do not use machine-learning techniques. These works have been treated according to the proposed taxonomy, as various similarities could be identified in the systems of each category.

For the direct and indirect classification, it is noticeable that several preprocessing and direct techniques were addressed, like the use of feature extraction techniques in sparse indirect formulations, optical flow in dense indirect formulations, direct motion formulations for direct proposals, and a combination of both techniques in hybrid processes. This allowed us to establish nine criteria that can be used to better select each system due to their influence on system performance, dimensionality, applicability and implementation. Table 2 summarizes the criteria for each classic monocular system.

**Fig. 26:**
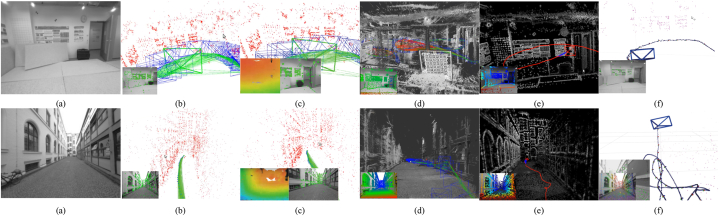
Examples of the results obtained by classic approach implementations. (a) represents the input image, (b) presents results obtained using the ORB-SLAM (indirect + sparse) algorithm [38], (c) presents results obtained using the DF-ORB-SLAM (indirect + dense) algorithm [82], (d) presents results obtained using the LSD-SLAM (direct + dense) algorithm [3], (e) presents results obtained using the DSO (direct + sparse) algorithm [24], and (f) presents results obtained using the SVO (hybrid) algorithm [23]. Top row results correspond to the indoor example sequence seq_01, and bottom row results correspond to the outdoor sequence seq_29 of the TUM-MONO dataset [127]. The examples were obtained through multiple executions of each algorithm in our previous comparative work [128]. For further information on the implementation and performance of each algorithm and category, we encourage the reader to address the paper [128] and the repository: https://github.com/erickherreraresearch/MonocularPureVisualSLAMComparison [129].

Additionally, we have run implementations available as open-source code. Fig. 26 introduces results obtained by implementing classic monocular SLAM, VO, and SFM systems in publicly available datasets where: Fig. 26.a. represents the input image, Fig. 26.b. presents results obtained using the ORB-SLAM (indirect + sparse) algorithm, Fig. 26.c. presents results obtained using the DF-ORB-SLAM (indirect + dense) algorithm, Fig. 26.d. presents results obtained using the LSD-SLAM (direct + dense) algorithm, Fig. 36.e presents results obtained using the DSO (direct + sparse) algorithm, and Fig. 26.f. presents results obtained using the SVO (hybrid) algorithm. While many machine learning techniques identified in this research are accessible as open-source code [6,10,66,[102], [103], [104], [105], [106], [107], [108], [109], [110], [111]], it was observed during implementation that, despite their adaptability to various input modalities like RBD-D or INS, they were not publicly available in their pure monocular RGB form. Additionally, some methods required external modules not included in the provided code, e.g., Refs. [104,107,109,112]. Thus, these methods could not be included as examples of monocular 3D reconstruction in Fig. 26.

## 7 Machine learning methods

In recent years, given the impressive developments in artificial intelligence, especially in deep learning, researchers have explored the possibility of extending the classic SLAM, SFM or VO techniques by implementing deep neural networks to perform different tasks to reduce depth estimation errors, improve 3D reconstruction quality, improve camera pose estimation, improve loop closure, estimate scale, and the like.

Another notable disadvantage of monocular techniques is scale ambiguity. Depending on the environment and camera calibration, objects or surfaces may be interpreted as having a different size than they actually do because monocular systems are not provided with an initial measurement of the scene's depth. Therefore, deep learning monocular methods commonly contribute to VO, SLAM, and SFM systems performing tasks like boosting VO from learning-based depth predictions, integrating depth representations into components (feature points, depth maps, and optimizers), performing additional tasks (like semantic segmentation) depth, optical flow and camera motion estimation and the like. Therefore, previous studies have researched replacing hand-crafted features with learned features [[130], [131], [132], [133]], implementing neural 3D representations [10,[134], [135], [136], [137], [138]], combining learned depth predictions with classical SLAM backends [2,66,139,140], and developing SLAM or VO systems trained end to end [138,[141], [142], [143], [144]].

Learning-based methods are generally less explicable, and they typically struggle when applied in unseen environments or with different camera calibrations. However, they still represent a promising solution for monocular visual SLAM, especially for pure rotation applications, slow motion, or motion without roll and pitch rotation, where SLAM or VO systems might face initialization or generalisation problems.

### 7.1 ML + indirect methods

Deep learning has helped 3D reconstruction systems perform many tasks that significantly improve the system's overall performance. Tasks range from simple parameter estimation for the initialization of SLAM, VO or SFM systems [145] to replacing an entire module on a SLAM pipeline (like depth or pose estimation) [139,144] or even providing new capabilities to the system (like semantic segmentation) [2,146]. As indirect methods, the studies in this category include preprocessing steps in their frameworks, in the form of feature extraction and optical flow extraction, performed as steps before the pose estimation or depth prediction processes. Hence, the quality of the final 3D map directly depends on the amount and quality of information extracted in this preprocessing step.

**Fig. 27:**
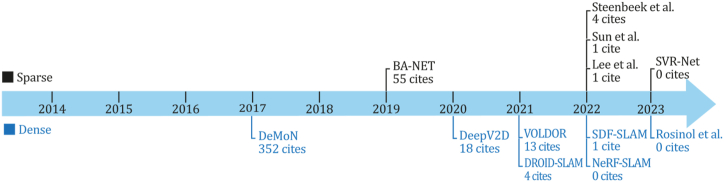
Most representative monocular SLAM, VO or SFM, ML indirect systems--a Timeline.

Like classic methods, ML + indirect methods can recover dense and sparse 3D environment maps, so both categories are discussed in sections 7.1, 7.1.1.2. Fig. 27 illustrates a timeline for the most representative SFM, VO, or SFM methods using machine learning indirectly working with image information. One of the most impressive ML methods belongs to this category, DeMoN [108], which has an outstanding ML citation score due to its impressive results.

#### 7.1.1 ML + sparse + indirect methods

Like classic methods, ML + Indirect methods can be internally classified as dense and sparse, depending on the density of the 3D reconstruction obtained. Sparse methods have the advantage of working with a small amount of information. Still, at the same time, this represents a constraint for the final reconstruction because some valuable information can also be excluded in pixel selection or feature extraction steps, which has also motivated the development of CNN architectures specialized in densifying the obtained 3D map [6,60], achieving promising results while preserving the performance advantages of working in a sparse paradigm.

**Fig. 28:**
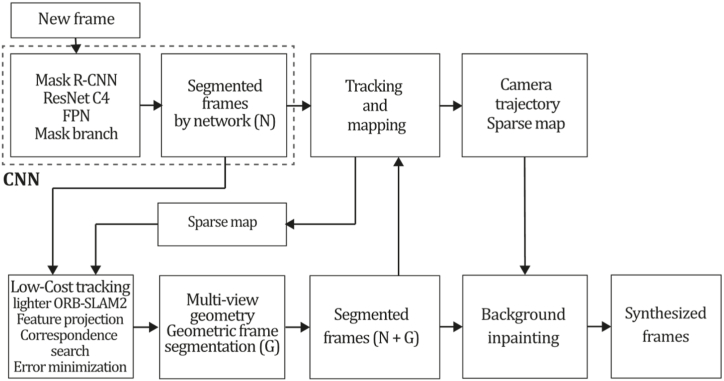
Diagram of DynaSLAM algorithm. Adapted from Ref. [102].

**DynaSLAM (2018).** In 2018, Bescos et al. [102] developed an algorithm for detecting, segmenting, and painting dynamic information available in the scene frame sequence information. This algorithm was built on top of a simpler and lighter version of the ORB-SLAM2 [38] algorithm, adding a CNN to segment a priori dynamic content on a pixel-by-pixel basis and a multi-view geometry approach to detect additional dynamic information that the CNN cannot detect. Dynamic information such as people and bicycles may be present in the scene image sequences used for 3D reconstruction. SLAM, SFM, and VO approaches can only discard a small fraction of this information as outliers because they are built over a static model assumption. In this way, given the impressive results that CNN has demonstrated for semantic segmentation tasks, authors proposed to integrate the Mask R-CNN of [147] to detect and segment regions of the image that potentially belong to the movable classes that the network was trained for e.q., person, bicycle, car, motorcycle, bus, train, boat, bird, cat, among others. The Mask R-CNN implementation extends the ResNet C4 and FPN backbones, adding a mask branch to predict a segmentation mask for each instance. The network was trained to detect the classes available on MS COCO [148], and if additional classes are required, it can be tuned with new training data. After potentially moving objects are segmented, the tracking thread, built over the ORB-SLAM2 algorithm, performs feature projection, correspondence search, reprojection error minimization, and camera pose optimization. In addition, some dynamic information, like a book carried by a person, might not be recognized by the CNN because they are not included in the CNN classes, so authors included a multi-view geometry stage, calculating the back-projections of each key point to compute the parallax angle which is used to differentiate movable from static objects. In this way, given the fact that geometric approaches present initialization problems due to their multi-view nature and learning approaches cannot recognize every possible moving object, DynaSLAM benefits from both approaches, overcoming initialization by including ML and combining the use of the CNN and multi-view geometry to track and segment moving objects. Experimental results proved that combining the geometric and CNN achieved better results than just one. To summarize, DynaSLAM segments the input images to remove the information of potentially dynamic objects from the scene images. It then performs low-cost tracking based on ORB-SLAM2. Next, a combination of CNN and multi-view geometry is applied to completely remove the information of moving objects, so that a background inpainting technique based on information from previous frames can be applied to complete the information removed from each frame; finally, accurate tracking and mapping can be applied over the static image segments of each frame. Fig. 28 describes the DynaSLAM algorithm inspired by Ref. [102].

**BA-Net (2019).** A prime relevant work in this category is BA-Net of Tang & Tan [59], which combines domain knowledge with deep learning. It enforces multi-view geometry constraints regarding feature-metric error by optimizing scene depths and camera motion by employing feature-metric bundle adjustment. Furthermore, the authors proposed a method to make the Levenberg-Marquard (LM) algorithm differentiable, enabling the network to learn suitable features by implementing a learned feed-forward multilayer perceptron (MLP) to predict the damping factor λ for the LM algorithm. This work also introduced a novel depth parameterization method to recover dense per-pixel depth, and depth maps are obtained by a convolutional neural network that generates a set of basis depth maps for each input image using an end-to-end learned generator. Then, the final depth is optimized as a linear combination of such basis performing a feature-metric BA.

This study formulated Bundle Adjustment as a differentiable BA layer, minimizing the distance between aligned network feature maps. In this regard, the feature-metric BA uses the network features of multiple images as inputs to optimize scene geometry and camera motion. Typically, BA with reprojection error is considered one of the best structure-from-motion approaches; however, with limitations: only information related to their feature types is utilized (typically corners, blobs, line segments); features must match each other, generating a large number of outliers, so the method depends on outlier rejection techniques. To overcome such issues, BA-Net proposed a feature-metric BA algorithm estimating scene depth and camera motion parameters, minimizing the feature-metric difference of aligned pixels:

(21) $$e_{i,j}^{f}\left(X\right)=F_{i}\left(\Pi \left(T_{i},d_{j}\cdot q_{j}\right)\right)-F1\left(q_{j}\right)\text{,}$$

where $F=\left\{F_{i}|i=1\ldots N_{i}\right\}$ are feature pyramids for the $I=\left\{I_{i}|i=1\ldots N_{i}\right\}$ images, $T=\left\{T_{i}|i=1\ldots N_{i}\right\}$ are camera poses, $X={\left[T_{1},T_{2}\ldots T_{N_{i}},d_{1},d_{2}\ldots d_{N_{j}}\right]}^{T}$ is the optimization parameter, Π is the function that projects scene points to image space, $d_{j}\in D=\left\{d_{j}|j=1\ldots N_{j}\right\}$ is the depth of $q_{j}$ pixel at the image, and $d_{j}\cdot q_{j}$ upgrades the $q_{j}$ pixel to its 3D coordinate. BA-Layer predicts T camera poses and D dense depth map during the forward process and back-propagates the loss of T and D to the F feature pyramids in the training process.

**Fig. 29:**
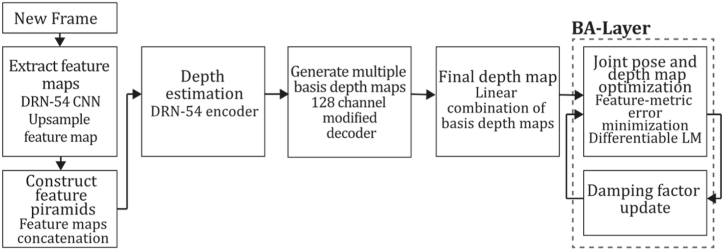
Diagram of BA-NET algorithm. Adapted from Ref. [59].

BA-Net network structure was built over the DRN-54 backbone containing a depth map generator, a feature pyramid constructor to build multiscale feature maps, and a BA-Layer optimizing depth map and camera poses using a novel differentiable LM algorithm. Making the LM algorithm differentiable is mandatory to solve the optimization problem. For this reason, the authors proposed a strategy to soften the if-else problems related to the LM algorithm by predicting the damping factor λ (proved sensitive to different data sets) using an MLP network. In brief, each new frame in BA-Net is taken as an input DRN-54 network, and convolutions and upsampling steps are performed to extract feature maps to construct feature pyramids by feature map concatenation. In addition, depth estimation is performed by passing information through the DRN-54 encoder with a modified 128-channel decoder generating multiple basis depth maps, linearly combined to create a final depth map that is jointly optimized with the pose by feature-error minimization using a differentiable form of LM algorithm. Fig. 29 describes the BA-Net algorithm inspired by the article [59].

**Fig. 30:**
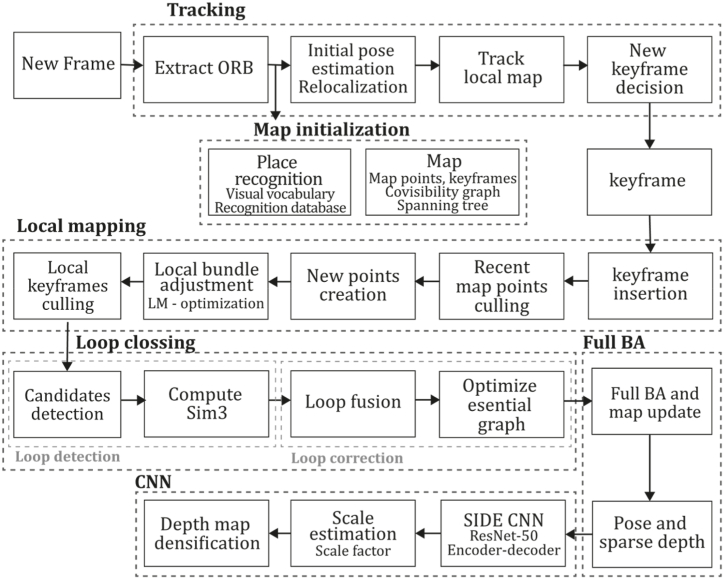
Diagram of Steenbeek et al., algorithm. Adapted from Ref. [6].

**Steenbeek et al. (2022).** Some implementations based on the ORB-SLAM popular classic approach integrate deep learning to boost the system, like [149,150]. However, a recent study in Ref. [6], "CNN-Based Dense Monocular Visual SLAM for Real-Time UAV Exploration in Emergency Conditions," developed based on the original ORB-SLAM2 [38] using its monocular input designed to work using a drone camera as image source combined with an implemented and trained CNN to densify the 3D reconstruction also obtaining the scale of the scene. In this work, authors pursued the goal of implementing this improved system to be applied in UAV exploration for emergency conditions because, in such conditions, the lightest and cheapest equipment is required to perform 3D reconstruction tasks; so inertial units, ultrasound, LIDAR, RGB-D or stereo cameras are unsuitable. Likewise, as previously described, the ORB-SLAM2 original algorithm is one of the best geometric sparse indirect approaches due to its excellent pose and trajectory estimation performance. In any case, ORB-SLAM2 3D reconstruction is not dense enough for many applications, and like the majority of SLAM systems, it faces scale ambiguity problems. To overcome these issues, the authors integrated the system with the CNN Single Image Depth Estimation (SIDE) algorithm [5], a monocular approach that worked efficiently with real-time monocular input. In this regard, the system was composed of a UAV RGB camera image sequence acquired with a commercial drone transmitted to a laptop executing the ORB-SLAM2 algorithm to estimate the pose and a sparse depth map that, in conjunction with RGB input images constituted the input of the neural network assigned to produce a scaled pose and a denser depth map. Then, they are stored and merged using the OctoMap solution [151]. The ORB-SLAM system was not remarkably modified, so just a couple of parameters were configured based on experimentation, like the selection of six pyramid levels for feature detection, 25 shared features were used to connect two frames, and the minimum interval between keyframes was set to 15 due to UAV fast movements. CNN SIDE network had an encoder-decoder architecture where the encoder processes input information, producing a feature map passed to the decoder, consisting of a ResNet-50 connected to pooling and linear transformation layers. The decoder part was inspired by the design of [152] using an up-sampling strategy conformed by up-projection blocks enabling high-level information to be passed through the network, progressively increasing and densifying map sizes. Then, the estimated depth map is compared with the triangulated SLAM features, obtaining a scale factor that keeps the reconstruction scaled to its real size. The CNN was trained on the NYU Depth v2 dataset [153] acquired using a Kinect sensor, so, as the drone and Kinect have different cameras, dataset images had to be cropped considering a different field of view and resampled using the intrinsic parameters of the new camera. The authors also included a median filter to strengthen the scale ratio, allowing the discard of outliers, which, based on experimentations, significantly improved the scale estimated by the CNN. Experimental results showed that even though the map was considerably densified by including the neural network, the result was not dense enough to be considered a dense approach, in addition to obtaining poor map quality compared to similar proposals. The authors reported that this may be because current SIDE algorithms cannot reconstruct 3D environments like stereo approaches. Such an effect can be attributed to using a low-level computer to run the whole pipeline, so the authors reported using more computational power for future work. Fig. 30 exhibits the Steenbeek et al. algorithm inspired by the article [6].

**Fig. 31:**
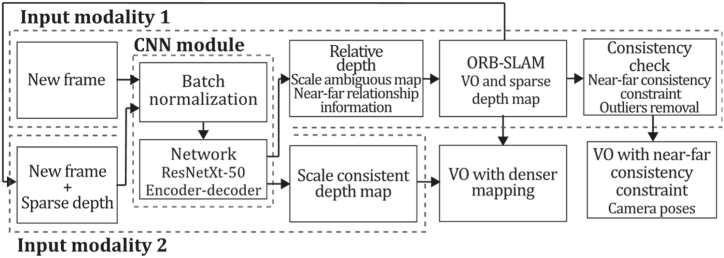
Diagram of Sun et al., algorithm. Adapted from Ref. [60].

**Sun et al. (2022).** Another recent work in this category is the system of Sun et al. [60], which is a framework that aims to improve VO by embedding a depth estimation module. The system was built on top of the popular classic ORB-SLAM [11] to extend its generalisation capabilities and densify the obtained 3D reconstruction. The authors were able to embed a neural network in ORB-SLAM, following the approach of DiverseDepth [154], embedding it as a depth module working in two modes contributing to odometry and mapping. In the first mode, the network is fed only with a monocular image predicting a relative depth map that does not provide scale information but contains a large amount of near-far relationship information useful to discard outliers, preserving an accurate set of points from which accurate camera poses can be calculated. In the second mode, the network is fed with an RGB image and a sparse depth map to predict scale-consistent depth that is then applied to perform a denser mapping. As previously mentioned, authors were especially interested in improving generalization capabilities, so ORB-SLAM was used as the SLAM base model due to its excellent capabilities for working in indoor and outdoor environments. The deep neural network used in this study was inspired by the proposal of [155], an encoder-decoder architecture designed for different working modes being randomly trained to alternate between them. Based on the ResNetXt-50 backbone, this network architecture was designed to predict a depth map from an affine invariant transformation with respect to the ground truth when fed with a single image predicting an accurate scale consistent depth map when a sparse depth map is conveniently added from the SLAM system. To extend network generalization capabilities, the authors used diverse training data coming from five different datasets: Taskonomy [156], DIML [157], ApolloScape [8], DiverseDepth [154], and RedWeb [158], which were recorded using high and medium accuracy annotation devices like LiDAR, Laser and Stereo setups having indoors and outdoors dense sequences. In the training stage, a FAST corner detector was used to sample sparse depth points for dense depth datasets to match the behaviour of the sparse depth coming from the SLAM system, and the two input modes were selected randomly, so two different loss functions were set for each mode. Experimental results showed that the ORB-SLAM system improved considerably in terms of generalization, strengthening indoor and outdoor scenes performance, improving depth estimation capabilities, and reducing the absolute trajectory error evaluated on the KITTI dataset. Despite such excellent results in visual odometry evaluation, it must be mentioned that the obtained depth maps were denser than ORB-SLAM, but in our opinion, compared to the reconstruction results of other dense proposals, they are not dense enough to be considered a dense indirect approach. Fig. 31 exhibits the Sun et al. algorithm inspired by the article [60].

**Lee et al. (2022).** One of the most recent approaches made on the sparse indirect category that incorporates deep learning in its framework is the work of [159], which aims to improve the accuracy and robustness of existing SLAM methods for autonomous driving. SLAM systems are essential for self-driving vehicles, as they allow the estimation of their position and the mapping of the environment using a single camera. The authors have formulated a deep neural network-based monocular SLAM system that integrates semantic segmentation and 3D geometry estimation to improve the system's accuracy. The proposed system was built upon ORB-SLAM, which was extended by implementing a deep neural network for estimating 3D geometry and adding a semantic segmentation module to improve the quality of the generated point cloud. The semantic segmentation module was integrated to help differentiate objects with similar geometries, like cars, pedestrians, and trees, using a labelling technique to improve the accuracy of the point cloud construction. It must be pointed out that the proposed monocular SLAM system's main innovation is its ability to leverage semantic segmentation for more accurate 3D environment reconstruction and mapping, where the system's deep neural network-based formulation allows it to learn and adapt to different environments and lighting conditions, making it robust to real-world scenarios. Moreover, the system uses a novel loss function that penalizes the translation and rotation errors differently to stabilize the estimated pose. In brief, the Lee et al. method was designed using three modules: localization mapping and segmentation. The localization module is responsible for selecting keyframes when mapping and segmentation modules complete each keyframe processing; this allows the extraction of corner features and the camera pose of each keyframe, estimated from the points of connected keyframes. The mapping module triangulates current and connected corner features to generate new 3D points. Then, it estimates a ground plane only using the points labelled as ground by the CNN, which also recovers the appropriate scale for camera poses. In this way, the mapping module estimates scale-corrected camera poses and 3D points. In contrast, the segmentation module performs deep-learning semantic segmentation over each down-sampled keyframe and refines the corner features existing on the keyframe by removing moving objects and areas with low-parallax, using the ERFNet CNN proposed in Ref. [160]. Thus, some additional improvements made in the Lee et al. approach are its scale correction in 3D mapping and its novel technique to remove factors that can lead to inappropriate mapping in each keyframe.

**Fig. 32:**
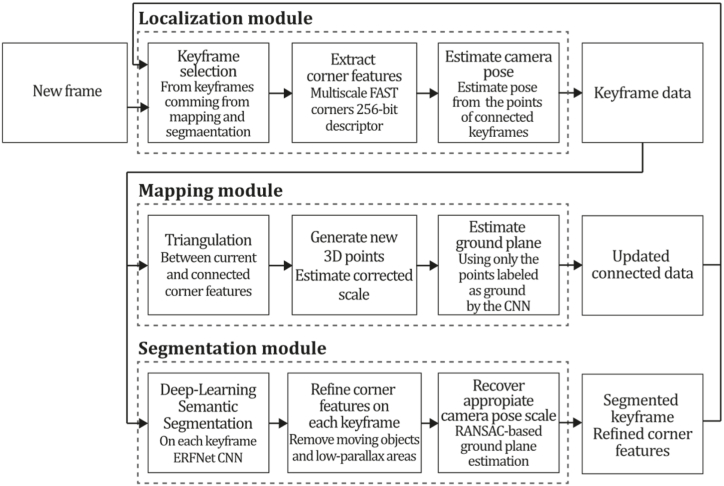
Diagram of Lee et al., algorithm. Adapted from Ref. [159].

To evaluate the proposed monocular SLAM system's performance, the authors conducted experiments on the KITTI benchmark dataset, which allowed them to demonstrate that their system outperforms the existing state-of-the-art monocular SLAM methods like ORB-SLAM, ORB-SLAM2, and Mask-SLAM. The proposed system achieved an average translational error of 0.19 %, significantly lower than the 0.40 % error achieved by the closest competitor. Additionally, it was observed that the system's semantic segmentation module improved the 3D reconstruction accuracy and enabled the generation of a detailed semantic map. Fig. 32 illustrates the Lee et al., algorithm inspired by the article [159].

**SVR-Net (2023).** The SVR-Net SLAM system [161] is a technique for simultaneous localization and mapping that was developed for both pure visual and visual + range sensors. Its purpose is to provide accurate 3D maps of unknown environments. A key contribution of this system is the incorporation of a Sparse Voxelized Recurrent (SVR) network, which is used to estimate the 3D location of key points in the scene. This improvement enables the system to achieve robust feature tracking, even in challenging lighting conditions or in the presence of occlusions. The SVR-Net SLAM system incorporates several other novel features, including an online learning algorithm that continually adapts to changing environmental conditions and graph optimization to refine the estimated camera poses and map structure. The system also includes loop-closure detection, which helps improve the map's overall accuracy and consistency. Compared to other SLAM systems that rely solely on visual or range sensors, the SVR-Net SLAM system offers several key advantages, such as its ability to produce highly detailed and accurate maps of complex environments, including structures with multiple levels and non-planar surfaces. Additionally, the system is computationally efficient, making it well-suited for real-time applications.

**Fig. 33:**
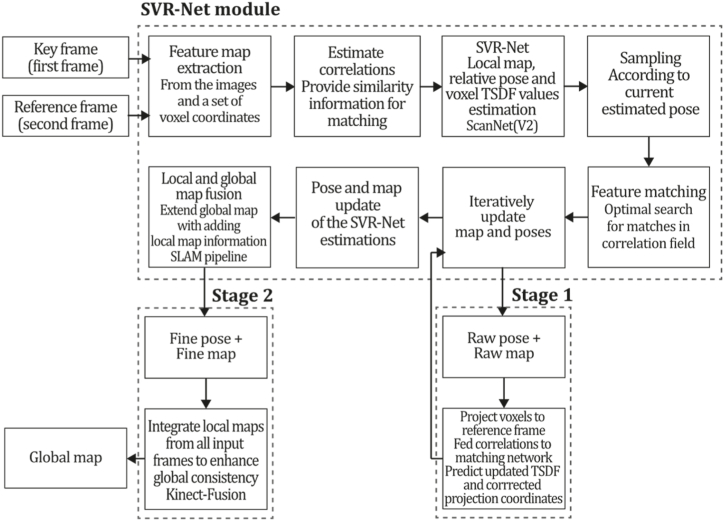
Diagram of SVR-Net SLAM algorithm. Adapted from Ref. [161].

In brief, SVR-Net SLAM utilises a coarse-to-fine approach to achieve efficient tracking and dense global mapping. It comprises two modules for processing raw data and refining the results. At the first stage, the system receives a pair of frames from where the raw pose and local map are estimated using the SVR network, so it starts representing the map as sparse voxels with TSDF (truncated signed distance function) values. Then, the map is extended using the first stage, the global map. In the second stage, SVR-Net SLAM performs voxel up-sampling, followed by pose and map refinement; then, the global map is extended by fusing it with the resulting fine local map. Specifically, the SVR-Net module was trained over the ScanNet(V2) proposal [162] as an end-to-end tracking and mapping network that takes a pair of RGB frames along with a set of voxel coordinates and outputs their local map, relative pose, and the TSDF values for the set of voxels. For this purpose, SVR-Net begins extracting feature maps for the images. Then, it transforms the feature map into feature voxels for each keyframe (first frame), and it estimates the correlations with the features of the second frame to provide similarity information for matching. Next, after sampling according to the currently estimated pose, a matching network matches the features (optimal match search in the correlation field), updates the pose, and maps iteratively. This way, voxels are projected to the second frame using the estimated pose from the previous iteration. The correlation values obtained from the sample are inputted into the matching network to predict the updated TSDF values and corrected projection coordinates. Then, the feature-matching step outputs are utilized to update the pose and map estimations of SVR-Net. Finally, the SLAM pipeline, built over the Kinect-Fusion method [42], uses the local map information to extend the global map and enhance its global consistency. Fig. 33 illustrates the SVR-Net algorithm inspired by the article [161].

#### 7.1.2 ML + dense + indirect methods

Indirect methods usually employ optical flow for depth prediction. It is important to note that a challenging task in VO frameworks is characterising feature location errors, which motion blur, occlusions, and viewpoint variations can corrupt. Specifically, the efficacy of direct methods typically relies on small motion and appearance-constancy assumptions, representing a limitation to providing robustness to scene variability, which reduces their applicability [163]. Recently, machine learning optical flow estimation, which can be described as a combination of rigid flow and an unconstrained flow describing general object motion, has reached state-of-the-art performance [164,165] demonstrating a superb level of accuracy, robustness, and generalization becoming an excellent solution, especially under challenging conditions like texture-less surfaces, motion blur, and large occlusions.

**Fig. 34:**
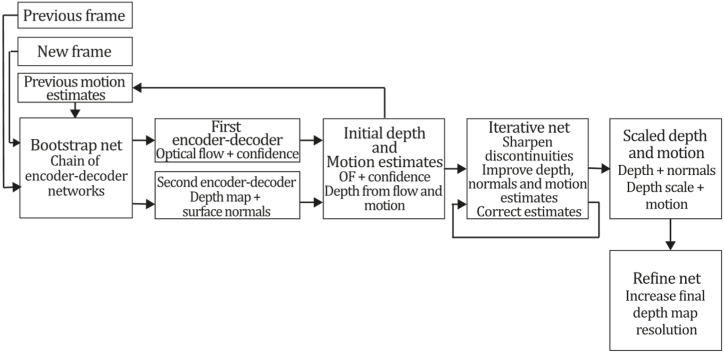
Diagram of DeMoN algorithm. Adapted from Ref. [141].

**DeMoN (2017).** Ummenhofer et al. [141] introduced a noteworthy example of this practice with their work on DeMoN, a motion system structure. DeMoN features the first CNN that jointly computes depth and camera motion using unconstrained image pairs, improving its predictions. The network also estimates surface normals, optical flow, and matching confidence. Furthermore, DeMoN can exploit motion parallax information, a powerful clue to generalizing new scenarios enabling egomotion estimation. For this purpose, the system alternates optical flow estimation with depth and camera motion estimation. The system includes an adapted version of FlowNet [166] to solve optical flow using a pair of images. The network outputs, depth maps, and motion vectors could be very different, so they must be balanced using loss functions; hence, L1 loss L for inverse depth values is:

(22) $$L_{depth}=\sum_{i,j}\left|sd^{*}\left(i,j\right)-\hat{d^{*}}\left(i,j\right)\right|\text{,}$$

where $d^{*}=\frac{1}{z}$ is the inverse depth, $\hat{d^{*}}$ is the ground truth, and s is the predicted scale. The normal and optical flow use L2 norm to penalize deviation from $\hat{n}$ and $\hat{w}$ ground truths:

(23) $$L_{normal}=\sum_{i,j}{\left\|n\left(i,j\right)-\hat{n}\left(i,j\right)\right\|}_{2}\text{,}$$

(24) $$L_{flow}=\sum_{i,j}{\left\|w\left(i,j\right)-\hat{w}\left(i,j\right)\right\|}_{2}\text{,}$$

then the loss for motion vectors is:

(25) $$L_{rotation}={\left\|r-\hat{r}\right\|}_{2}\text{,}$$

(26) $$L_{traslation}={\left\|t-\hat{t}\right\|}_{2}\text{,}$$

where r=θv is a minimal parameterization of a rotation with angle θ and v axis, and t is the translation vector. Finally, the scale-invariant loss for a discrete scale invariant gradient g is:

(27) $$g_{h}\left[f\right]\left(i,j\right)={\left(\frac{f\left(i+h,j\right)-f\left(i,j\right)}{\left|f\left(i+h,j\right)\right|+\left|f\left(i,j\right)\right|},\frac{f\left(i,j+h\right)-f\left(i,j\right)}{\left|f\left(i,j+h\right)\right|+\left|f\left(i,j\right)\right|}\right)}^{T}\text{,}$$

(28) $$L_{grad{\;d}^{*}}=\sum_{h\in \left\{1,2,4,8,16\right\}}\sum_{i,j}{\left\|g_{h}\left[d^{*}\right]\left(i,j\right)-g_{h}\left[\hat{d^{*}}\right]\left(i,j\right)\right\|}_{2}\text{,}$$

to cover gradients at different scales, five different h spacings are used so the network can compare within a neighbourhood for every pixel. Finally, the system applies scale invariant gradient loss to each optical flow component to enhance the smoothness of the estimated flow fields, enhancing sharpness for motion discontinuities. Summarizing, DeMoN takes both images as input to predict the depth map using the first image and the relative pose using the second image. The CNN comprises a chain of encoder-decoder networks that iterate over optical flow, the depth map, and egomotion estimation. Therefore, the system is constituted of three main components: the bootstrap net that takes an image pair as input and outputs the initial depth and motion estimates, using encoder-decoder networks that calculate optical flow and confidence maps of the flow; the iterative net casting to sharpen discontinuities, improve the scale of the depth values, correct wrong estimates of initial bootstrapping network and improve depth, normal and motion estimates; and a final refinement net that increases the resolution of the final depth map. Fig. 34 illustrates the DeMoN algorithm inspired by the article [141].

**Fig. 35:**
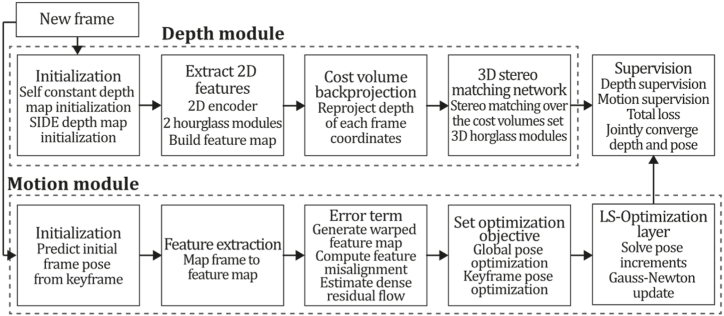
Diagram of DeepV2D algorithm. Adapted from Ref. [144].

**DeepV2D (2020).** Teed & Deng [144] proposed DeepV2D, an end-to-end learned pipeline that alternates depth and motion modules to predict depth and camera pose. The motion module uses depth estimates to predict camera pose, which becomes more accurate as depth estimates converge. Both modules were built over neural networks ruled by geometric principles but combined in an end-to-end differentiable architecture to perform structure from motion. The depth module takes camera motion as input and calculates depth prediction, whereas the motion module takes depth as input, estimating a motion correction term as output. The deepV2D depth module builds a cost volume using learned features where information is added to multiple viewpoints using a pooling layer. In this way, the depth module is composed of: a 2D feature extractor, which is a 2D encoder using two stacked hourglass networks that map each frame to a dense feature map; a cost volume back-projection module that reprojects coordinates in each frame for each possible depth; and a 3D stereo matching network performing stereo matching over a defined set of cost volumes. The motion module outputs a set of perturbations as error terms used to update the camera pose. Motion module requires of: initialization, where a frame is chosen as a keyframe, and relative motion is predicted; feature extraction, where learned features map every frame to a feature map; error term, taking two frames and an hourglass network predicting residual flow between their feature maps; and optimization layer, where pose increments are solved applying a Gauss-Newton update. Although DeepV2D was mainly developed and focused on depth estimation, it can be turned into an SLAM system by training the neural network to directly map optical flow to camera motion without requiring optical flow supervision. As a contribution, this work differs from DeMoN [141] because its motion module can be used on a variable number of frames. DeepV2D features a new motion estimation architecture called Flow-SE3, which sets it apart from other works like DeMoN and DeepTAM. This architecture allows the system to apply geometric constraints on camera motion, reducing reprojection error and benefiting from end-to-end training. Fig. 35 illustrates the DeepV2D algorithm inspired by the article [144].

**Fig. 36:**
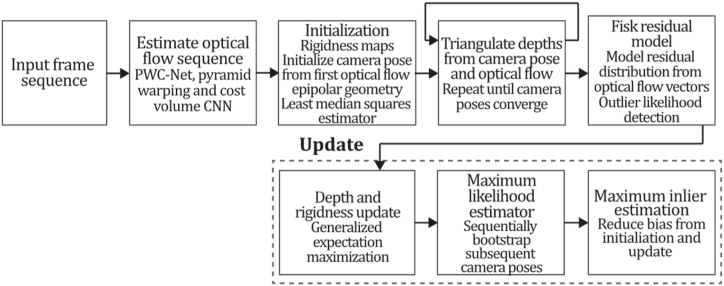
Diagram of VOLDOR algorithm. Adapted from Ref. [163].

**VOLDOR (2021).** Another recent work worth mentioning is the VOLDOR VO system developed by Min & Dunn [163]. This system utilises log-logistic depth residuals to perform visual odometry. It takes an externally estimated optical flow input, computed by machine learning estimators, and a probabilistic model to develop a VO pipeline that does not require feature extraction, RANSAC estimation, or local bundle adjustment processes to output camera pose and depth maps. It was observed that optical flow residuals tend to conform to a log-logistic distribution, which is the base of its probabilistic framework ruled by a Fisk-distributed residual model that jointly estimates camera motion, pixel depth, and motion-track confidence using a generalized expectation maximization (EM) formulation. Similarly, the VOLDOR inference framework uses a generalized expectation-maximization formulation to infer depth and rigidness, a maximum likelihood estimator (MLE) to bootstrap subsequent camera pose, a maximum inlier estimation to mitigate MLE criteria, and a forward-backwards algorithm to infer rigidness from the image reduced to hidden Markov chains. VOLDOR is agnostic to the optical flow input estimator, so PWC-net [165] was used to estimate the external dense optical flow input. PWC-net is a CNN that uses pyramidal processing, warped features, and cost volume to warp the CNN features of a second image using the optical flow estimate of the current image, being 17 times smaller and easier to train than FlowNet2 [164] and comprised of a feature extractor, an optical flow estimator and context networks.

In summary, VOLDOR uses a Fisk residual model to perform inference from a sequence of optical flows extracted by an external estimator (PWC-net system in this case), where the first camera pose is initialized from epipolar geometry from the first optical flow using the least median-square estimator. After that, depths are triangulated by two-view triangulation using optical flow and the first camera pose. Finally, the update is performed by inference over the Fisk residual model, where a generalized expectation maximization model updates depth and rigidness. Then, a maximum likelihood estimator is used for sequentially bootstrapping subsequent camera poses, while a maximum inlier estimation criterion is used to reduce bias generated at initialization and update processes. In conjunction with the Fisk residual model and maximum inlier estimation, this CNN system achieved optimum results in an ablation study and outperformed similar optical flow systems in the KITTY and TUM RGB-D datasets. Fig. 36 describes the VOLDOR algorithm inspired by the article [163].

**Fig. 37:**
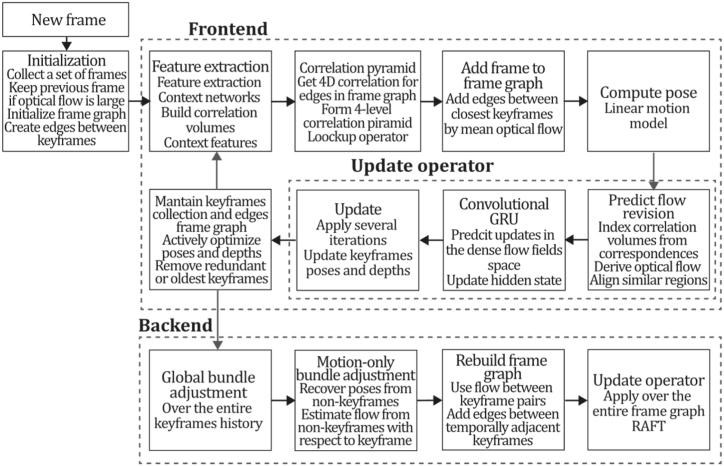
Diagram of DROID-SLAM algorithm. Adapted from Ref. [167].

**DROID-SLAM (2021).** One of the recent notable works is DROID-SLAM, which stands for 'Differentiable Recurrent Optimization-Inspired Design' [167]. This deep learning-based system performs iterative camera pose updates and estimates depth maps through a dense bundle adjustment layer. DROID-SLAM predicts updates in the dense flow fields domain using a Gated Recurrent Unit (GRU) to produce an error correction term in the dense correspondence field and a depth map. DROID-SLAM can perform real-time localization and mapping by using frontend and backend threads. The front-end inputs new frames, extracts features, selects keyframes and executes local bundle adjustment. At the same time, the backend thread simultaneously performs global bundle adjustment over the entire set of historic keyframes. DROID-SLAM is a differentiable architecture built over RAFT [168] (Recurrent all-pairs field transforms for optical flow), facilitating the system to work with optical flow, performing recurrent iterative updates so instead of updating the optical flow, the system updates depth maps and camera poses obtained through a differentiable bundle adjustment layer calculating the Gauss-Newton update to maximize compatibility with current optical flow estimate. DROID-SLAM can work with monocular, RGB-D, and stereo input, demonstrating an exceptional performance on the TartanAir, EuRoC, TUM-RGB-D, and ETH3D-SLAM benchmarks, outperforming acknowledged classic and learned-based monocular systems in most sequences. The authors noted that the monocular system has a major limitation in its high computational cost. During the experimental stage, 24 GB of GPU memory was required to run the EuRoC, TartanAir, and ETH3D sequences. Fig. 37 presents the DROID-SLAM algorithm inspired by the article [167].

**SDF-SLAM (2022).** Another important and relevant work in this category is SDF-SLAM [146]. It is a monocular system based on the ideas of ORB-SLAM but with a novel feature extraction method and a dense semantic network for estimating dense depth labels. This places SDF-SLAM in the dense category of the taxonomy. As previously stated, the original ORB-SLAM system was designed to extract texture information, such as edges or corners, by using a combination of algorithms, including SIFT, SURF, ORB, and others, to extract feature points from each pair of adjacent frames based on their similarity. Feature points of each frame are projected to the next frame using the change in camera pose, where PnP is used to minimize errors between the projected point and actual matching. Next, PnP converts those feature points into 3D coordinates using the camera pose, fusing all the features and creating a depth map.

**Fig. 38:**
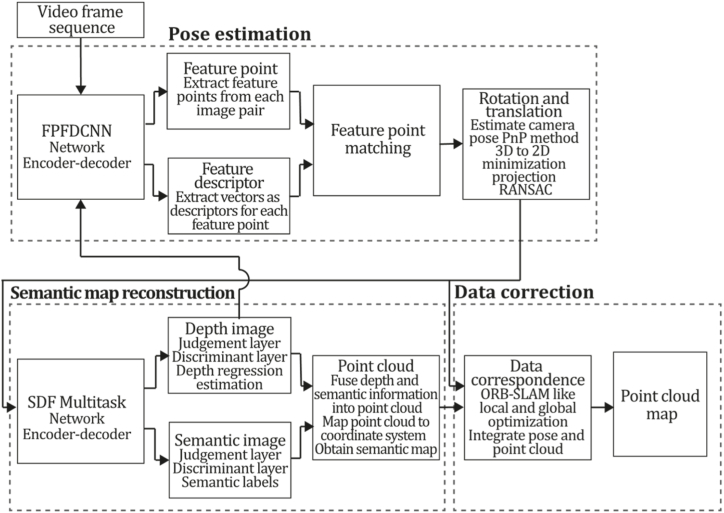
Diagram of SDF-SLAM algorithm. Adapted from Ref. [146].

Nevertheless, the map created by ORB-SLAM is not dense enough for many applications and lacks semantic information which a machine can recognize. This way, the proposal aims for accurate camera trajectory estimation and recovery of a three-dimensional scene semantic reconstruction map. Therefore, the semantic and depth fusion proposal fuses camera poses with depth and semantic information at the frame level to obtain a 3D semantic reconstruction achieved by implementing three key components: a feature point and CNN feature description (FPFDCNN) trained to perform feature extraction from a pair of images calculating vectors as descriptors for every feature point; a deep semantic fusion CNN (SDFCNN) trained to perform simultaneous semantic segmentation and depth prediction from RGB images, allowing the system to drastically reduce the number of estimated parameters; a monocular visual SLAM system embedding a deep learning system adding a data correction module to globally optimize the point cloud to obtain consistent point clouds. For such purposes, SDF-SLAM uses two neural networks where FPFDCNN is used to extract feature points from a pair of adjacent frames, matching them to obtain feature-matching pairs. Then, a minimization process is applied to obtain camera rotation and displacement matrixes, fed along with the image to SDFCNN to recover a dense map and a semantic segmentation. Finally, the data calibration module is fed with dense map, pose, and semantic segmentation information to perform global and local optimization tasks, creating a three-dimensional semantic map. The FPFDCNN was built over the encoder-decoder architecture constituted by an input layer, encoder layers (to extract the feature map), decoder layers (to restore the original image size and feature descriptors), output layers, and concatenate layers. For one thing, SDFCNN was implemented using a unified semantic down-sampling layer to process information in feature maps and an up-sampling layer for feature extraction and restoration that uses discriminative layers for feature classification, probability estimation, and depth regression estimation. Experiments carried out using SDF-SLAM on the TUM dataset demonstrated a clear improvement over the classic ORB-SLAM [11] and LSD-SLAM [37] methods, also demonstrating a significant improvement in semantic segmentation quality compared with CNN-SLAM [2], achieving 90 % accuracy for point cloud prediction and 67 % in semantic labelling. Another advantage of SDF-SLAM is that this technique can be fused with many classic SLAM approaches, which authors demonstrated with reported results running the same network architectures over the DSO framework. Fig. 38 illustrates the SDF-SLAM algorithm inspired by the article [146].

**NeRF-SLAM (2022).** Article [169] presents NeRF-SLAM, a monocular method for indoor scene reconstruction using normal priors, to enhance reconstruction accuracy and detail. The method combines the advantages of Neural Radiance Fields (NeRF) and the DROID-SLAM [167] approach as a tracking module to recover real-time accurate and detailed reconstructions of indoor scenes. NeRF-SLAM was developed to address limitations such as challenges in handling dynamic environments and occlusions and limited scalability in many monocular systems. NeRF-SLAM framework aims to overcome these limitations by using an implicit neural representation to model the scene's geometry and appearance, incorporating normal priors to improve reconstruction detail and accuracy. The framework consists of three components: a frontend that estimates the camera poses and generates sparse 3D point clouds, a backend that fuses the sparse point clouds, generating the final implicit representation of the scene, and a normal prediction module trained to predict surface normals from the implicit representation. The tracking front was built over DROID-SLAM, gathering dense depth maps and poses for each keyframe taken from an eight-keyframe sliding window. For this purpose, DROID-SLAM estimates the optical flow of each pair of frames using an approach inspired by Raft, which is a Convolutional GRU that computes the flow and weight using the correlation of each two-frames and a guess of the current optical flow. Then, DROID-SLAM solves the dense BA problem using the flows and weights representing the 3D geometry as a parameterized set of inverse depth maps, which helps solve the BA problem efficiently using a linear least squares formulation. Then, the method computes marginal covariances for the depth maps and poses, which are used along with the depth, poses, and input RGB images to optimize the radiance field's parameters and refine the camera poses. As a result, the mapping backend uses the information obtained by the tracking front end to supervise the radiance field, which leads the system to obtain biased reconstructions. In summary, in NeRF-SLAM, the tracking thread continuously minimizes the BA reprojection error over an active window of keyframes. At the same time, the mapping thread optimizes all the keyframes obtained from the tracking thread, where the tracking thread only generates a new keyframe each time the mean of the optical flow between the previous keyframe and the current frame is higher than a threshold.

**Fig. 39:**
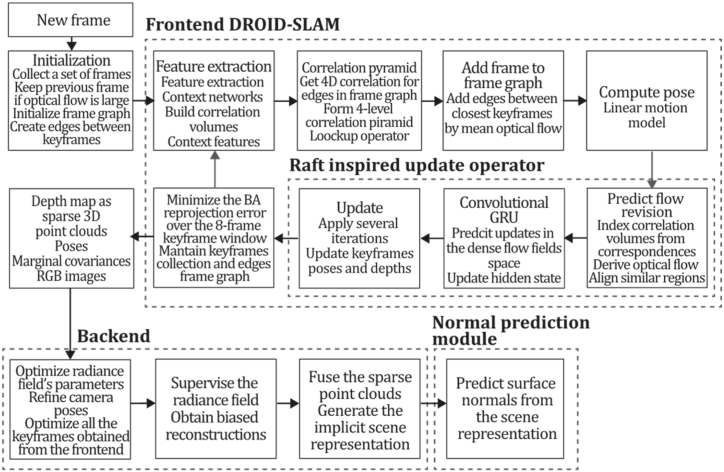
Diagram of NeRF-SLAM algorithm. Adapted from Ref. [169].

From extensive experiments on several datasets comparing NeRF-SLAM with state-of-the-art methods, it was observed that NeRF-SLAM outperformed existing methods in terms of reconstruction accuracy, detail, and robustness to dynamic scenes and occlusions, making NeRF-SLAM a promising approach for indoor scene reconstruction that combines the strengths of neural networks and SLAM. Fig. 39 illustrates the NeRF-SLAM algorithm inspired by the article [169].

**Rosinol et al. (2023).** The proposed probabilistic volumetric fusion method is a novel approach for dense mapping and reconstructing 3D environments from RGB-D (colour and depth) or monocular RGB data. It outperformed most existing state-of-the-art methods in accuracy, robustness, and speed. The proposed method extends the traditional volumetric fusion method by introducing a probabilistic framework based on Gaussian Process Regression (GPR). This probabilistic volumetric fusion method presents a novel approach to 3D environment reconstruction from RGB data that allows a more robust and accurate reconstruction by incorporating uncertainty in the depth measurements, which is crucial for robust decision-making in uncertain environments. Its probabilistic framework and real-time performance provide superior accuracy and robustness over existing state-of-the-art methods. The main improvement of this method over existing techniques is the ability to handle many frames in real-time. In brief, this method pursues the goal of fusing dense, noisy depth maps weighted by probabilistic uncertainty estimations into a volumetric map. For this purpose, the system uses the Droid-SLAM frontend to recover pose estimates and dense depth maps, which were also adapted to recover dense uncertainty maps. The Droid-SLAM framework is applied to obtain a set of inverse depths per keyframe to solve the BA problem. Then, the inverse depth uncertainties are computed from the information matrix of the underlying bundle adjustment problem, using the marginal covariances for the per-pixel depth variables, which allows recovering sparse depth maps that are up-sampled using the Raft upsampling operator, and the depth variances are computed considering a nonlinear uncertainty propagation. Next, a fusion strategy is applied to produce a volumetric map using an uncertainty-aware volumetric mapping technique which, in contrast to Droid-SLAM (which uses an ad-hoc depth filter), uses the estimated uncertainties of each depth map on a probabilistic volumetric fusion model to provide a robust and mathematically sound alternative to reconstruct the scene geometry. Finally, the 3D mesh is extracted from the volume based on a maximum uncertainty bound. Only the voxels that present an uncertainty below this bound are meshed to extract the surfaces.

**Fig. 40:**
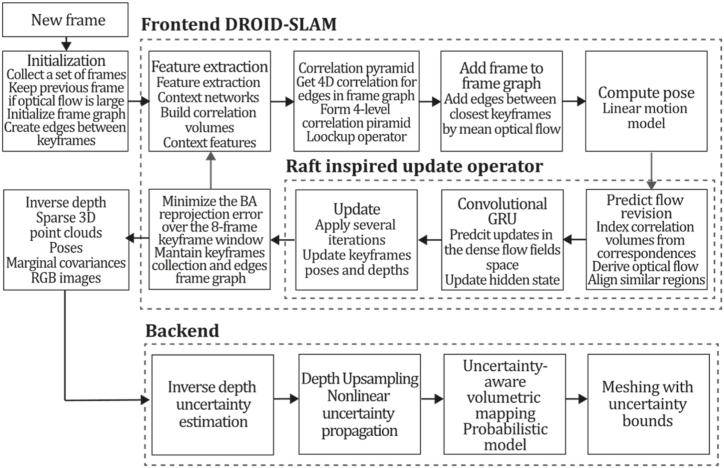
Diagram of Rosinol et al. algorithm. Adapted from Ref. [170].

The Rosinol et al. system was evaluated on different datasets, including synthetic and real-world data, and it was compared with several state-of-the-art methods, including fusion-based methods, such as KinectFusion and ElasticFusion, and deep-learning-based methods, such as MVSNet and NERF, demonstrating its robustness and generalization capability. The accuracy and quality of the proposed method make it suitable for use in augmented/virtual reality and computer graphics, including virtual game environments and movie production. This makes it a promising area for future research and development. In addition, its outstanding real-time performance makes it suitable for robotics applications, such as autonomous navigation in unknown environments. Fig. 40 illustrates the Rosinol et al. algorithm inspired by the article [170].

### 7.2 ML + direct methods

In recent years, machine learning has emerged as a promising avenue for research in monocular 3D reconstruction. This is due to its ability to address well-known limitations of monocular systems, such as scale ambiguity, motion blur, texture-less surfaces, and repetitive patterns. Classic direct systems face significant challenges, including reliance on a good initialization, the brightness constancy assumption, their performance in low-illumination environments, and their ability to generalize to unseen environments. Accordingly, in the past decade, researchers have made interesting contributions to overcome these challenges by embedding neural network architectures in classic SLAM, VO, or SFM proposals, enhancing performance and, in most cases, have demonstrated to outperform their classic versions.

**Fig. 41:**
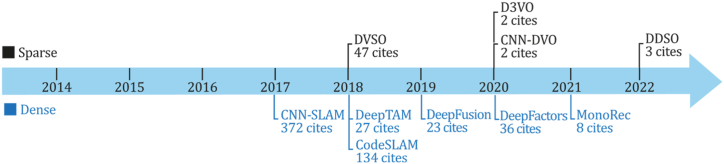
Timeline for the most representative monocular SLAM, VO or SFM, ML direct systems.

ML + Direct methods, like the rest of the taxonomy categories, may be divided into dense and sparse, depending on the density of recovered 3D reconstruction, so both of these alternatives are discussed in sections 7.2, 7.2.1.2. Fig. 41 describes a timeline for the appearance of the most representative ML + direct methods in the last decade. Two of the most representative ML approaches belong to this category: CNN-SLAM and CodeSLAM, significantly contributing to the state-of-the-art, yielding impressive citation scores providing two interesting paths for researchers: the integration of semantic segmentation and the use of encoder-decoder architectures, respectively.

#### 7.2.1 ML + dense + direct methods

Similar to classic SLAM methods, machine learning dense formulations can be classified as dense or sparse depending on the sparsity of the final reconstruction. Some authors have proposed improving pose and depth estimation and estimating parameters such as scale factors or initialization terms. In addition, as shown in the previous section, the possibility of training networks to densify the map output of classic SLAM systems exists; thus, classic sparse methods were added to this category with their densified machine learning versions. As shown in Fig. 41, most ML direct approaches belong to the dense category due to the excellent results machine learning has achieved in densifying their 3D reconstructions.

**CNN-SLAM (2017).** An exciting work that achieved the goal of fusing deep convolutional depth prediction with a dense direct system is the work of [2]. It uses CNN-predicted depth maps in monocular SLAM to overcome the issue of estimating the absolute reconstruction scale by learning from examples and getting rid of assumptions and geometric constraints. The obtained CNN depth map is used as an initial guess to perform a dense reconstruction, which is then refined by the LSD-SLAM direct approach. Most monocular SLAM systems have restraint scene absolute scale, so the final reconstruction scale is ambiguous.

In contrast, the CNN-predicted depth map provides absolute scale information, aiding the pose estimation process for more accurate pose trajectory and scene reconstruction. Also, most monocular systems fail under pure rotational camera movements due to the lack of a stereo baseline in contrast to a CNN-predicted depth map which does not face these problems because each frame is estimated individually. The system was built over the depth prediction method of [152] with the direct SLAM system by using the CNN-predicted depth map of every new keyframe as prior information for the SLAM system. The network architecture presents a first stage based on ResNet-50 initialized with pre-trained weights on ImageNet to estimate the environment scale. Then, a second part of the network replaces the last pooling and fully connected layers with a sequence of residual up-sampling blocks. Finally, drop-out is applied, and a convolutional layer outputs the predicted depth map.

The CNN depth prediction and direct SLAM are fused using the uncertainty map. Uncertainty map $U_{k_{i}}$ is the elementwise distance between the depth map of the current keyframe $k_{i}$ and the closest keyframe $k_{j}$:

(29) $$U_{k_{i}}\left(u\right)={\left(D_{k_{i}}\left(u\right)-D_{k_{j}}\left(\Pi \left(\zeta T_{k_{j}}^{k_{i}}V_{k_{i}}\left(u\right)\right)\right)\right)}^{2}\text{,}$$

where ζ is the camera intrinsics matrix, $V_{ki}\left(u\right)=\zeta^{-1}\dot{u}D_{ki}\left(u\right)$ represents a 3D element of the vertex map computed from the depth map of the current keyframe, u is a generic depth map element, with $\dot{u}$ being its homogeneous representation, $v=\Pi \left(\zeta T_{kj}^{ki}V_{ki}\left(u\right)\right)$, and ${\tilde{U}}_{k_{j}}$ is the uncertainty related to the CNN estimation. The depth and uncertainty map of a frame are fused with those of the closest keyframe to improve the accuracy of every initialized keyframe where the uncertainty of the nearest keyframe is:

(30) $${\tilde{U}}_{k_{j}}\left(v\right)=\frac{D_{k_{j}}\left(v\right)}{D_{k_{i}}\left(u\right)}U_{k_{j}}\left(v\right)+\sigma_{p}^{2}\text{,}$$

next, the two uncertainty and depth maps are fused using the following weighted expressions:

(31) $$D_{k_{i}}\left(u\right)=\frac{{\tilde{U}}_{k_{j}}\left(v\right)∙D_{k_{i}}\left(u\right)+U_{k_{i}}\left(u\right)∙D_{k_{j}}\left(v\right)}{U_{k_{i}}\left(u\right)+{\tilde{U}}_{k_{j}}\left(v\right)}\text{,}$$

(32) $$U_{k_{i}}\left(u\right)=\frac{{\tilde{U}}_{k_{j}}\left(v\right)∙U_{k_{i}}\left(u\right)}{U_{k_{i}}\left(u\right)+{\tilde{U}}_{k_{j}}\left(v\right)}\text{.}$$

**Fig. 42:**
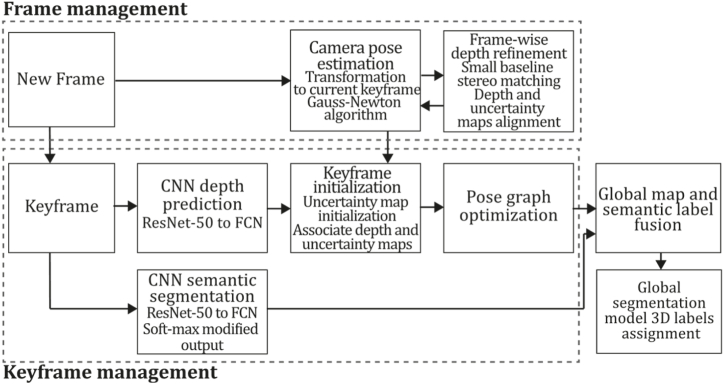
Diagram of CNN-SLAM algorithm. Adapted from Ref. [2].

In CNN-SLAM, the authors performed semantic segmentation by relying on the idea that the same network can be used to perform high-dimensional regression tasks. Therefore, CNN-SLAM could be considered the first example that jointly performed semantic segmentation and 3D reconstruction, opening up a new research avenue where multiple 3D regression tasks can be performed along with depth prediction. To summarize, the CNN-SLAM system collects keyframes as a subset of visually distinct frames whose pose is refined by pose graph optimization. Simultaneously, camera pose estimation is performed by estimating the transformation of the current frame to the closest keyframe. The CNN-predicted depth map is obtained only for keyframes with high frame rates. An uncertainty map is created by measuring pixel-wise confidence for every prediction. At the same time, a second convolutional network conducts semantic label fusion to predict a semantic segmentation for each frame. Finally, the relative pose is optimized through a pose graph on keyframes. Fig. 42 illustrates the CNN-SLAM algorithm inspired by the article [2].

**DeepTAM (2018).** Zhou et al.'s DeepTAM [171] is an improved version of DTAM [27], formulated as a deep learning problem using two CNN architectures for tracking and mapping tasks. The proposal's main contributions include a tracking network designed for incremental frame-to-keyframe tracking, a multiple hypothesis proposal for camera pose estimation, a mapping network that combines depth estimation with image priors, and a depth refinement strategy that combines CNN architectures with a narrow band technique. The tracking network of DeepTAM aligns the current image with a keyframe containing depth and the colour image to infer the camera pose, essentially computing 2D to 3D correspondence between the image and the keyframe. For this purpose, the authors used an encoder-decoder-based architecture to learn 6 DOF estimation poses related to a keyframe and use the optical flow, ensuring the network learns to exploit the relation between a pair of images. Hence, the decoder of the tracking network performs two tasks: optical flow prediction and pose hypothesis generation. They also used a coarse-to-fine strategy to learn to track large and small camera motion, so the authors trained three tracking networks to generate the pose hypothesis at different resolutions. Consequently, the tracking network performs an incremental pose estimation where each network is specialized for a distinct resolution level. Hence, each network computes a pose estimate where the latest pose guess is taken as a virtual keyframe at each resolution, thereby tracking the camera pose, and the final pose estimate is the product of all the incremental pose updates.

**Fig. 43:**
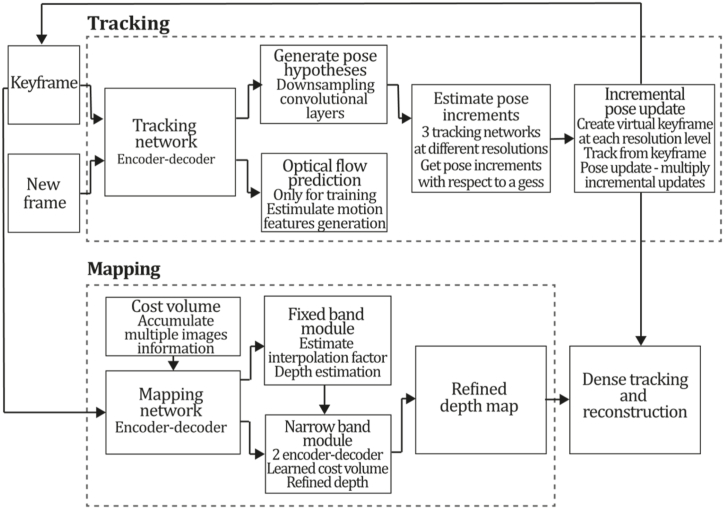
Diagram of DeepTAM algorithm. Adapted from Ref. [171].

The DeepTAM mapping network was developed based on the plane sweep stereo concept. It accumulates multiple-image information in a cost volume and extracts the depth map through a CNN using image-based priors and the accumulated depth information. To improve depth prediction, the authors used a network that iteratively refines the depth prediction using the cost volume within a narrow band around the geometry estimate of previous frames. In DeepTAM, the mapping architecture consists of fixed- and narrow-band modules. The fixed band module takes the input image and the cost volume to output an interpolation factor and depth estimation. The narrow band module runs iteratively to build a learned cost volume; finally, the depth map is obtained by applying a differentiable soft argmin operator, and a second encoder-decoder takes the depth estimation and the keyframe image producing a refined depth map. It is no secret that machine learning techniques tend to suffer from overfitting, so authors took special care in designing DeepTAM architecture and its configuration for the learning problem so that the network cannot learn simple shortcuts that may result in generalization problems. In addition, the authors used data augmentation techniques applied in the training process and used train datasets like SUN3D [172] and SUNCG [173] to learn to track 6 DOF motion. Thus, some of the key advantages of DeepTAM are its strong tracking capabilities, which, on experimental evaluations, have proven to achieve better results than its predecessor CNN-SLAM and its ability to process more than two images to refine the depth map, avoiding drift. Fig. 43 presents the DeepTAM algorithm inspired by the article [171].

**Fig. 44:**
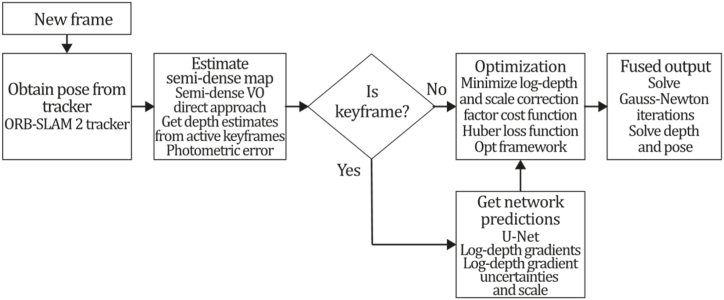
Diagram of DeepFusion algorithm. Adapted from Ref. [174].

**DeepFusion (2019).** In Ref. [174], the authors proposed a 3D reconstruction system named DeepFusion. Using a monocular SLAM system, the system aims to obtain dense scaled depth maps and poses in real time. Predicted depth gradients are used as a constraint on the neighbourhood pixels to ensure global reconstruction consistency and learned uncertainties are used to fuse the different modalities. This proposal was formulated by fusing the sparse monocular system ORB-SLAM2 [38] output with a CNN's depth and gradient predictions in a probabilistic framework using uncertainties obtained by predicting the per-pixel mean and variance fused with the geometric constraints. In this system, the network needs to be executed once by a keyframe, so the depth map is optimized for each frame constantly using new geometric constraints. The shape and absolute scale are obtained using a cost function that includes per-pixel losses based on the CNN depth predictions and depth estimates using pairwise constraints from the network depth gradient predictions. Network architecture was based on U-Net [175] trained to predict log-depth, modified by adding three decoders to predict log-depth uncertainties, log-depth gradients, and log-depth gradient uncertainties as log-depths were selected over depth or inverse depths due to their scale-invariance properties. An associated uncertainty was integrated into the formulation to fuse CNN output with the monocular SLAM system estimates for every pixel in each of the log-depth and gradient images, which was performed by making the network learn to predict the mean and variance by training the network on the SceneNet RGB-D dataset, using the maximum likelihood cost function:

(33) $$L_{NN}\left(\omega \right)=\sum_{i}\frac{\left(y_{i}-\mu_{\omega ,i}\left(x\right)\right)}{\sigma_{\omega ,i}{\left(x\right)}^{2}}+\log\left(\sigma_{\omega ,i}{\left(x\right)}^{2}\right)\text{,}$$

where ω is the set of weights, x is the set of input pixels, $y_{i}$ is the ground truth for each pixel, and $\mu_{\omega ,i}\left(x\right)$, and $\sigma_{\omega ,i}{\left(x\right)}^{2}$ are mean and variance predictions. As mentioned earlier, the monocular depth estimation was performed using the [65] method, as depth values were searched over the epipolar line for every pixel in the keyframe with sufficient texture by minimizing the sum of squared differences of five equally spaced points. As DeepFusion is a direct dense system, depth is estimated by minimizing the photometric error:

(34) $$E_{i}=I_{i}\left(\Pi \left(\zeta T_{WC_{1}}^{-1}T_{WC_{0}}\Pi^{-1}\left(x_{i},d_{semi,i}\right)\right)\right)-I_{0}\left(x_{i}\right)\text{,}$$

where ζ is a matrix containing the camera intrinsics, $T_{WC_{0}}$ and $T_{WC_{1}}^{-1}$ correspond to keyframe, and reference frame poses, respectively. $I_{*}\left(\cdot \right)$ is the function that returns intensity values for each pixel, Π is the projection and homogenization function, $\Pi^{-1}\left(x_{i},d_{semi,i}\right)$ is the back-projection function, which returns a 3D point for each pixel having $d_{semi,i}$. depth. Once the minimum is found, an interpolation between two steps is done to find the optimal depth. Next, uncertainty for each semi-dense measurement is approximated by $\sigma_{i}^{2}={\left(J^{T}J\right)}^{-1}$ (where J is the Jacobian of error function). After that, depth estimates and uncertainties are converted to log space matching CNN outputs. To sum up, for every new RGB image, the system obtains the pose using ORB-SLAM2 [38]. Next, the semi-dense depth estimates for each keyframe are updated using the [65] algorithm. Then, if a new keyframe is selected, CNN predicts log-depth gradients associated uncertainties, and if the current frame is not a keyframe, it is used for optimization where a set of log-depth values and scale correction factors are minimized in a cost function using Opt optimization framework [176]. Finally, the output is fused using an associated uncertainty formulation, solving depth and pose jointly. Fig. 44 describes the DeepFusion algorithm inspired by the article [174].

**CodeSLAM (2018).** Bloesch et al.'s CodeSLAM [134] is a system designed to generate a dense and concise representation of scene geometry using a code derived from a single image and an intensity image. This code consists of a small set of unknown parameters, enabling a compact representation of scene geometry without compromising reconstruction details. The encoding strategy was designed based on the principle that encoding the full depth of an image is unnecessary, as most of the information is already available in the image intensities. Hence, the code retains only that part of the information that cannot be retrieved from image intensities. Each depth map becomes a function of the image I and an unknown code c that will be estimated using a neural network D=D(I,c). The CNN used in this system consists of a combination of the monocular depth estimation architecture of [177] and the encoder network of [178] configured to obtain a small code size, where the accuracy is maximized by training to minimize reconstruction error. This architecture takes the intensity image as input for a U-Net to obtain a high-dimensional image representation. Then, the extracted intensity features are encoded and decoded, obtaining the image depth map. Encoding and decoding procedures are carried out by a typical down-sampling architecture achieved by stride convolutions, followed by a variational component in the bottleneck of an autoencoder, composed of two fully connected layers that sample the code using a Gaussian distribution subject to regularization cost, then decoded by an upsampling architecture using bilinear interpolation and concatenating with processed image intensities of previous layers.

**Fig. 45:**
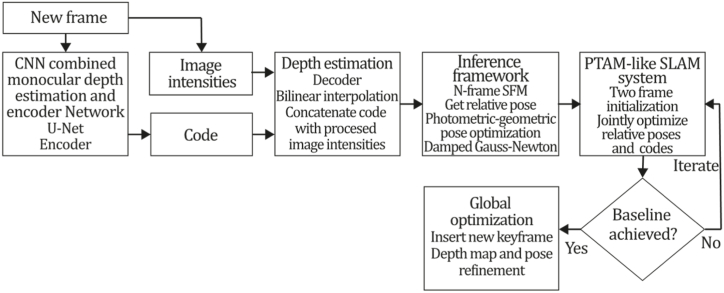
Diagram of CodeSLAM algorithm. Adapted from Ref. [134].

The network also outputs the mean and depth uncertainty using four pyramid levels. CodeSLAM includes an inference framework based on an N-frame SFM paradigm where codes and poses are optimized using loss functions based on photometric and geometric errors. Thus, residuals and Jacobians are computed to apply a damped Gauss-Newton algorithm to find the optimal code and pose for each frame. Then, tracking is performed by estimating the pose in the current keyframe with respect to the existing keyframe map. The SLAM system for this approach was developed following the concept of PTAM [71,114] by alternating tracking and mapping, where the system initializes by taking two frames and jointly optimizing their relative poses and codes. Then, after some iterations, a baseline is achieved, and a keyframe is inserted to perform global optimization. It must be mentioned that the CodeSLAM geometry encoding proposal allows joint optimization of geometry and motion, which showed improved performance on the EuRoC dataset over its predecessors, giving the system robustness to deal well with fast and pure-rotational motions. Fig. 45 illustrates the CodeSLAM algorithm inspired by the article [134].

**DeepFactors (2020).** Czarnowski et al. [10] proposed DeepFactors, a probabilistic dense SLAM system that employs an efficiently learned depth map representation. The system combines classical SLAM principles with priors learned from data over geometry in a probabilistic factor-graph framework. The system includes three types of errors/factors that are used to estimate camera trajectory and scene geometry: photometric factor being the direct difference between image intensities and target image; reprojection being the difference between the observed and hypothesized matched landmark locations; and the sparse geometric factor, computing the difference in scene geometry comparing depth maps of a frame and its target image. Ablation studies performed in this work took photometric error as a baseline system, as it is a direct method concluding that reprojection error improves local minimum avoidance and increases the convergence rate.

**Fig. 46:**
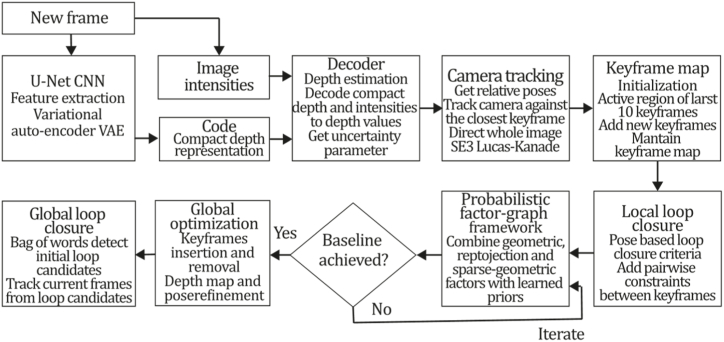
Diagram of DeepFactors algorithm. Adapted from Ref. [10].

In contrast, geometric error introduces a prior about the world and helps pin depth maps to form a single reconstruction in texture-less areas lacking photometric information. This way, the study introduced the idea of using the three-consistency metrics to improve the performance of monocular reconstruction systems. DeepFactors was built based on CodeSLAM [134] but with a different mapping backend formulating the problem as a multiview bundle adjustment, introducing the use of a three-factor error formulation, and improvements on improvements on keyframing, map maintenance, and tracking. In contrast to DeepTAM [171], this approach relies less on network generalization, performing a more robust optimization correcting wrong predictions from the network where neural networks are priorly focused on obtaining an image-conditioned manifold from which the optimization process will be executed. To achieve real-time performance, the authors used a Generalized Purpose GPU (GPGPU), modifying the CodeSLAM network using a U-Net to extract features from the input images, reducing image size by multiple convolution steps. Next, a Variational Auto-Encoder (VAE) learns an optimizable compact depth representation; then, the decoder interprets this information into depth values. The encoder and decoder architectures are conditioned by the features using concatenations. An additional output of the feature network is an uncertainty parameter used in a negative log of a Laplacian likelihood loss as a metric for supervising the reconstructed depth, whereas the predicted depth map is supervised with an L1 loss. Fig. 46 exemplifies the DeepFactors algorithm inspired by the article [10].

#### 7.2.2 ML + sparse + direct methods

Direct methods, such as classic approaches, can be divided into two subsections depending on the final density 3D map obtained from the reconstruction. These subsections are dense and sparse. By adding machine learning contributions to this formula, we get the ML + Dense + Direct and ML + Sparse + Direct method classifications. Therefore, the ML + Sparse + Direct category is commonly implemented due to the impressive results that sparse direct classic methods, like DSO [24], have achieved. In this section, we introduce the most successful machine learning integrations made over the DSO system and improved versions, where the integration of neural networks was performed to enhance map quality, tracking, generalization capabilities and overcoming well-known failure modes like brightness assumption violations, motion blur, repetitive textures among others. Some of the proposals described in this section have obtained denser reconstructions [141,180] and produced dense or semi-dense output maps due to the incorporation of machine learning. However, those methods were incorporated into this category because such systems use a sparse set of selected points as a working principle.

**DVSO (2018).** An early approach in this category is the “Deep Virtual Stereo Odometry” (DVSO) proposed by Yang et al. [140]. DVSO is a system that improves the performance of DSO [24] by incorporating deep learning and the stereo extension of DSO (Stereo DSO) [12]. It creates a virtual stereo odometry system using monocular input as the only source of information. The study's objective was accomplished by integrating a semi-supervised neural network to predict depth maps from image input. These depth maps were then used to initialize sparse depths for the DSO algorithm at a consistent depth scale.

**Fig. 47:**
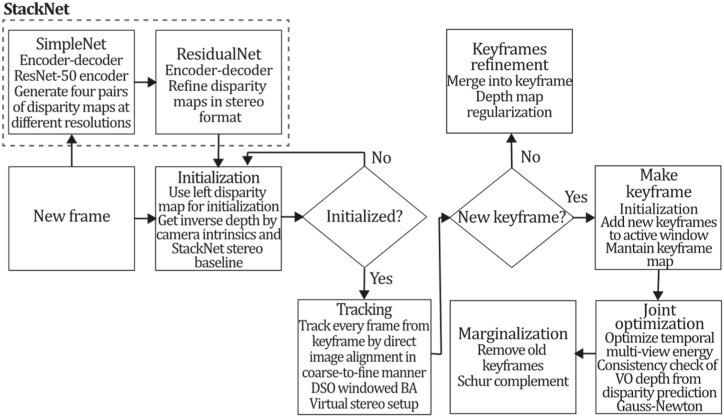
Diagram of DVSO algorithm. Adapted from Ref. [140].

Additionally, odometry was improved using a novel virtual stereo term that coupled the depth estimated in the DSO windowed bundle adjustment with depth predictions. This monocular semi-supervised neural network was designed to predict refined disparity estimates incorporating three key ingredients to enhance the system: a self-supervised learning based on a photo-consistency approach relying on image reconstruction loss, a supervised learning approach based on Stereo DSO depth predictions used as ground truth, and a two-stage refinement strategy for network predictions using an architecture of stacked encoder-decoder networks. The network proposed in this work was called StackNet, stacking two networks, SimpleNet and ResidualNet, inspired by DispNet [179] using an encoder-decoder scheme. SimpleNet is an encoder-decoder architecture, using ResNet-50 as an encoder-decoder with skip connections that unprojects feature maps to their original size, generating four pairs of disparity maps at different resolutions. On the other hand, ResidualNet was inspired in FlowNet 2.0 [164] implemented to refine disparity maps predicted by SimpleNet, managing this information in a stereo format as reconstructed right image (obtained by warping the input image into the view of a rectified stereo image), generated left image and the reconstruction error. Again, learning was designed to be guided by a loss function that takes into account five terms in a linear combination: self-supervised loss, to measure the quality of the reconstructed image; supervised loss, to take into account the deviation of the predicted disparity map from the disparity sparse set of pixels estimated by DSO; left-right disparity consistency loss, to ensure consistency between the left and right disparity images; disparity smoothness regularization, to promote that the predicted disparity map be locally smooth; and the occlusion regularization that penalizes the total sum of absolute disparities to improve system behaviour for background depths and complex transitions at occlusions. With the carefully tuned scheme, the authors could outperform classic monocular VO systems and even stereo VO systems, although using only the monocular input. Fig. 47 illustrates the DVSO algorithm inspired by the article [140].

**CNN-DVO (2020).** One of the most successful implementations in this category is the work of Cheng et al. [103], called CNN-DVO. They integrated deep CNN depths as priors for initialization, local bundle adjustment, and loop closure in a sparse direct visual odometry system using deep learning. CNN-DVO is the first direct framework that fully integrates depth prediction with initialization, tracking, and marginalization components to jointly optimize all model parameters, including inverse depth, camera pose, and affine model. The work also proved the benefit of using extra constraints given by depth prior, including fixing the arbitrary initialization scale to a constant value, significantly suppressing scale drift during tracking, and recovering the 3D measure correspondence in loop closure while maintaining a consistent scale. In addition, the work proposes an improvement on the point selection strategy using near low and high gradient regions, which helps to avoid clustering, making the tracking thread more adaptable to large stereo baselines and promoting robustness of pose estimation in extreme motion situations.

Additionally, joint optimization is broken into two steps: solving the pose from a coarse prior and correcting the depth from the pose values given in the first stage. Inspired by Ref. [64], this proposal divides the SLAM problem into coarse tracking and map refinement. In addition, authors used a keyframe selection strategy based on marginalization results and from a pose graph with a BoW database of ORB features extracted from each keyframe uniformly selected from the image space to guarantee that mapped points can be reproduced in the next frame, contributing to a proper loop closure. The method incorporates dynamic up-sampling and down-sampling of points in multiscale image rectangular subsections. After generating the tracking points set and applying DSO point selection, the system slides kernels of size $2^{x}$ over the whole image and computes a histogram of gradients within the region defined by the kernel. Points in low-gradient pixel locations are randomly discarded when the region is sparse. The final tracking set is the union of the points selected from each kernel. The SE(3) keyframe pose and inverse depth are parameterized and composed as a minimization problem. The system uses the Monodepth2 model [180] as the CNN depth predictor, giving the information to estimate the next frame camera pose and refine pose estimation. Then, Gauss-Newton optimization is performed for every selected point to refine optimal reprojection locations and recover inverse depth in the epipolar line. In this case, the variance of each observation is defined as:

(35) $$\sigma_{d}^{2}=\lambda {\left(\frac{d^{\prime }}{6}\right)}^{2}+\left(1-\lambda \right)\sigma_{d,obs}^{2}\text{,}$$

where $d^{*}$ is the inverse depth, $d^{\prime }$ is the predicted inverse depth, λ is the weight (empirically set to 0.57), $\sigma_{d}$ is the standard deviation that, in this case, corresponds to a linear combination of the inverse depth and the observation variance $\sigma_{d,obs}$. As a result, this empirical proposal provides room where noisy depth predictions converge. Then, depth prior is incorporated as a residual $r_{id}$ to penalize inverse depth deviations between keyframes to properly scale estimated transformations between them:

(36) $$r_{id}=\left(I_{j}\left[p^{\prime }\left(T_{i},T_{j},d^{\prime },\zeta \;\right)\right]-b_{j}\right)-\frac{t_{j}e^{a_{j}}}{t_{i}e^{a_{i}}}\left(I_{i}\left(p\right)-b_{i}\right)\text{,}$$

where $T_{i},T_{j}$ are the camera poses, ζ is the camera intrinsics matrix, $a_{i}$, $a_{j}$, $b_{i}$, $b_{j}$ correspond to affine bright coefficients, $I_{i}$ and $I_{j}$ are a pair of keyframes, $p^{\prime }$ in $I_{j}$ are projected points from p in $I_{i}$. First, a windowed optimization like DSO is performed, then BRIEF descriptors are extracted from each keyframe, maintaining the same BoW database used for loop detection query. Next, RANSAC PnP is applied to initialize transformations, and Sim(3) transformation is optimized by 3D-2D geometric constraints. CNN-DVO uses a depth filter to initialize inverse depth estimation for each selected point, so error variance is formulated as:

(37) $$\sigma_{d^{*}}^{2}=\alpha^{2}\left({\zeta \;}_{d}+{\zeta \;}_{d}\frac{J_{d}\Sigma J_{d}+J_{d}^{\prime }\Sigma J_{d}^{\prime }}{J_{d}\Sigma J_{d}}+\sigma_{d}^{2}\right)\text{,}$$

where the inverse depth $d^{*}=d\left(I_{0},I_{1},d^{\prime },\xi ,\Pi \right)$ is a function of depth prior and geometrical projection input, $d^{\prime }$ is the inverse depth prior, ξ is the relative transformation matrix, Π is the projection function, $c_{d}$ is the normalization constant (empirically set to 0.2), $J_{d}$ is the Jacobian of d, $J_{d}^{\prime }={\left[dx,-dy\right]}^{T}$ is the conjugated Jacobian of d, $\Sigma ={\left[I_{x},I_{y}\right]}^{T}\left[I_{x}.I_{y}\right]$ is the input error covariance, and α is the proportionality constant defined in Ref. [65]. Then, the inverse depth prior is initialized as a function $N\left(d^{\prime },\sigma_{d}^{2}\right)$, then the system performs a Gauss-Newton optimization to shrink the inverse depth upper bound for each map point candidate through inverse depth residual $r_{id}$:

(38) $$r_{id}=\sum_{i\in P_{i}}{\left\|I_{1}\left[\Pi \left(q_{i},d,\xi \right)-e^{a}I_{0}\left(q_{i}\right)+b\right]\right\|}_{\gamma }\text{,}$$

where γ is the Huber norm, and $P_{i}$ is the residual pattern. Depth prior narrows the search region, turning the method tenable to large baseline stereo problems. Then, the depth propagation for tracking is performed by updating inverse depth defined by a formulation like the Kalman filter step where a noisy observation $N\left(d^{\prime },\sigma_{d^{\prime }}^{2}\right)$ is fused with the geometrical propagation prior $N\left(d_{1},\sigma_{d_{1}}^{2}\right)$:

(39) $$d_{1}^{\prime }\left(d_{1},d^{\prime }\right)=N\left(\frac{\sigma_{d_{1}}^{2}d^{\prime }+\sigma_{d^{\prime }}^{2}d_{1}}{\sigma_{d_{1}}^{2}+\sigma_{d^{\prime }}^{2}},\frac{\sigma_{d_{1}}^{2}\sigma_{d^{\prime }}^{2}}{\sigma_{d_{1}}^{2}+\sigma_{d^{\prime }}^{2}}\right)\text{.}$$

**Fig. 48:**
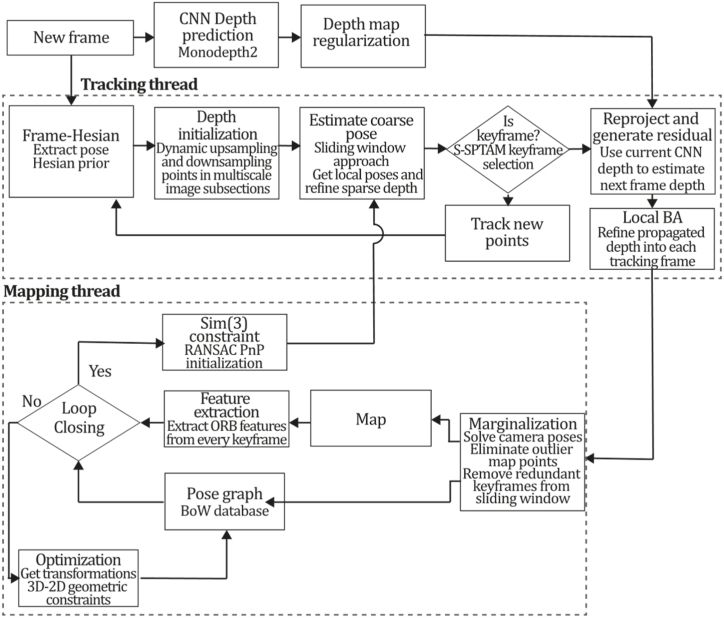
Diagram of CNN-DVO algorithm. Adapted from Ref. [103].

To sum up, CNN-DVO is based on the principles of direct SLAM. The tracking thread extracts a pose hessian prior to every new frame, and depth is initialized by dynamically up-sampling and down-sampling points in multiscale rectangular image subsections. A coarse pose is estimated using the sliding window approach. Simultaneously, a depth prediction from CNN is extracted and regularized. This prediction is then used to estimate the depth of the next frame, which is continuously propagated into each tracking frame and refined by local bundle adjustment. Next, the tracking thread performs marginalization to solve camera pose and eliminate outlier map points, removing redundant keyframes resulting in a refined depth map and a pose graph using a BoW database and ORB features extracted for loop closing. Finally, Sim (3) transformations are optimized through 3D-2D geometric constraints. Fig. 48 describes the CNN-DVO algorithm inspired by the article [103].

**D3VO (2020).** D3VO is a system developed by Yang et al. [139] that combines the DSO VO system [24] with deep learning to improve the original system in monocular depth prediction, camera pose, and photometric uncertainty estimation tasks. Unlike DVSO, which relies on the depth measurement extracted by StereoDSO [12] and uses a semi-supervised approach, D3VO can be trained from only stereo videos without any external depth supervision signal. The process is carried out by incorporating DepthNet and PoseNet, convolutional neural networks following a U-Net-like architecture built upon MonoDepth2 [180], extending its capabilities to predict brightness transformation parameters and photometric uncertainty. This self-supervised neural network was incorporated to address typical direct sparse issues like illumination changes, non-Lambertian surfaces, high-frequency areas, motion blur, and moving objects that breach the brightness consistency assumption.

**Fig. 49:**
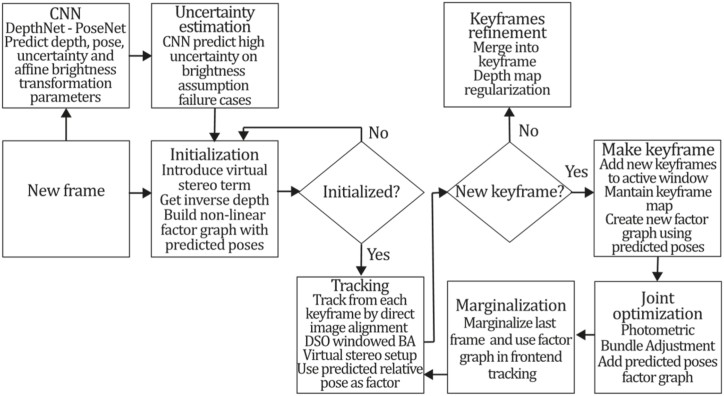
Diagram of D3VO algorithm. Adapted from Ref. [139].

Consequently, pixels that are likely to breach this assumption are down-weighted. Specifically, the self-supervised training network and the VO system share similar photometric objectives, so the authors proposed using learned weights to replace the weighting function based on photometric residuals, which in DSO was empirically set. In D3VO, the self-supervised neural network not only estimates depth pose uncertainty but also estimates affine brightness transformation parameters to align the illumination of each set of training images in a self-supervised manner. So, photometric uncertainty for each pixel can be predicted from the distribution over the possible brightness values, tightly incorporating predicted information in the tracking front-end and the photometric bundle adjustment performed in the backend. More specifically, failure cases of brightness constancy assumption introduce noise in the system, so the authors applied the heteroscedastic aleatoric uncertainty for neural networks in Ref. [181] to predict a subsequent probability distribution for each parameterized pixel with its mean and variance. Thus, predictive uncertainty enables CNN to update the weighting of the residual dependent on the input data to improve the model to noisy data. Instead of initializing depth values randomly like most classic SLAM systems, D3VO uses depth values predicted by DepthNet having scale prior information. In addition, instead of a constant velocity model, this approach uses poses predicted by PoseNet to build a nonlinear factor graph, where every new frame is tracked from the current keyframe by direct image alignment. As a result, D3VO uses predicted poses as initializations in the tracking front-end and the optimization backend while adding depth information as a regulator for the energy function to perform photometric bundle adjustment. Experimental results demonstrated that the combination of D3VO techniques allowed the system to outperform its predecessor's performance, achieving excellent trajectory estimation results comparable to those obtained by state-of-the-art visual-inertial odometry approaches, although relying only on monocular input. Fig. 49 illustrates the D3VO algorithm inspired by the article [139].

**MonoRec (2021).** An impressive and successful proposal made over the ideas of DSO [24] is the MonoRec system of Wimbauer et al. [182], which, more than a VO or SLAM system, is conceived as a 3D reconstruction system built by integrating two neural networks as a MaskModule and a DepthModule to extend the applicability of monocular SFM to static and dynamic environments. Depth prediction methods that fuse deep learning have emerged in two modalities, as multiview stereo (MVS) [[183], [184], [185]] and monocular depth prediction [139,186,187] systems. However, not all focus on obtaining an incremental geometric representation of the scene. MVS methods consider information from multiple views to recover depth information working over a stationary assumption in the environment, so they are affected by the presence of moving objects.

In contrast, monocular depth prediction methods perform well on moving objects as they make depth predictions from a single image. However, these methods heavily rely on the object's appearance from the camera's perspective, which is influenced by the camera's intrinsic and extrinsic characteristics. MonoRec combines both approaches for benefit using two modules, MaskModule and DepthModule. MaskModule uses encoded information obtained from multiple views as cost volume tensors built using Structural Similarity Index Measure (SSIM) to identify moving pixels, which are removed by downweighting their voxels in the cost volume trained to determine each pixel's probability for belonging to a moving object by detecting inconsistent geometric information in a set of cost volumes. MaskNet uses pre-trained ResNet-18 features to encode prior information, and then a U-Net-adapted architecture with skip connections predicts a mask of probabilities. The DepthModule was built based on a U-Net architecture predicting an inverse depth map using the complete cost volume and an image as input. After that, the wrong predictions from moving objects are removed by multiplying the predicted depth map with the mask. MonoRec was trained using a multistage training approach using a semi-supervised loss formulation not requiring LiDAR ground truth measures where stages are bootstrapping, MaskModule refinement, and DepthModule refinement. In bootstrap, each module is trained separately, where the DepthModule uses a semi-supervised loss combining a self-supervised photometric term and an edge-aware smoothness term based on [180] using sparse depth maps from the DVSO system.

**Fig. 50:**
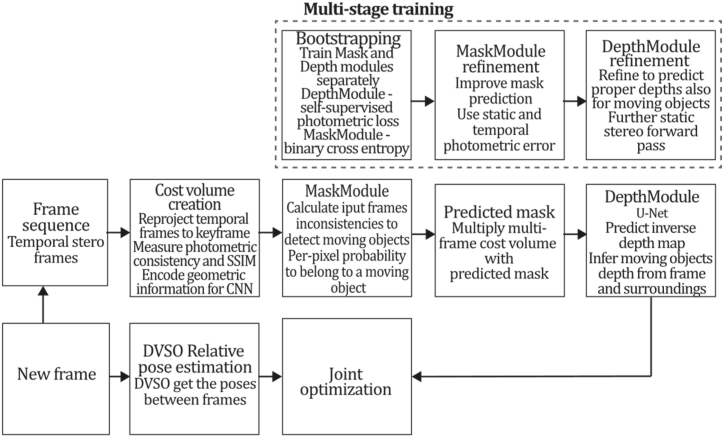
Diagram of MonoRec algorithm. Adapted from Ref. [182].

In contrast, MaskModule is trained using a mask loss corresponding to the binary cross entropy between the predicted mask and ground truth. In the MaskModule refinement stage, a supervised mask loss is added as a regularizer to stabilize training. New gradients are added to their corresponding structure in the cost volume to improve mask prediction capabilities and prevent overfitting. Finally, the DepthModule refinement stage was incorporated to make the DepthModule able to predict depths for moving objects by performing further stereo forward passes using the resulting depth map as prior for moving objects. Experimental results performed over the KITTI, TUM-Mono, and Oxford Robot-Car datasets proved the impressive capabilities of MonoRec regarding obtaining scene semi-dense point clouds. Ablation studies proved the importance of using MaskModule and DepthModule refinements to improve moving object detection and mapping. Fig. 50 describes the MonoRec algorithm inspired by the article [182].

**DDSO (2022).** Zhao et al.'s [145] DDSO is a monocular SLAM proposal that builds upon the DSO visual odometry framework [24]. The DDSO system incorporates deep neural networks trained in an unsupervised manner to enhance the accuracy and robustness of the DSO system. DSO estimates the pose from photometric information, eliminating the need to calculate feature descriptors. However, it has been demonstrated that direct methods are sensitive to photometric changes between frames and highly dependent on good initialization, which can be challenging in complex environments. In DSO, pose accuracy depends highly on the image alignment algorithm, which obtains inter-frame poses, optimizing the initial pose from a constant motion model. However, this constant motion model assumes that the current inter-frame pose is the same as the last, unsuitable for strong movements, motion blur, or highly repetitive textures. The constant motion model does not have prior camera pose information in DSO initialization, so a unit matrix is used empirically. In this way, DDSO improves the performance of DSO by enhancing this constant motion model using deep-learning-based pose estimation for initialization, replacing the unit matrix, and, for tracking, adding inter-frame pose information. This novel initialization is achieved using a CNN called TrajNet, which is trained unsupervised using four geometric constraints. One of the main contributions of this study was the novel pose-to-trajectory constraint. TrajNet is formulated and trained along with a depth estimation network called DepthNet, where the geometry constraints between the outputs of each pair of deep models serve as a training monitor. So the key supervisory signal for TrajNet is integrated of: the view reconstruction constraint to take into account the view reconstruction errors coming from pairs of consecutive frames based on the same depth map; the smoothness constraint, which is a loss term that promotes the representation of geometric details; the depth alignment constraint, to promote scale consistency in the outputs of DepthNet by aligning the scale of adjacent depth maps; and the novel pose-to-trajectory constraint that aims to improve the trajectory generation ability of the network promoting the scale consistency for three consecutive poses taking into account inter-frame poses.

**Fig. 51:**
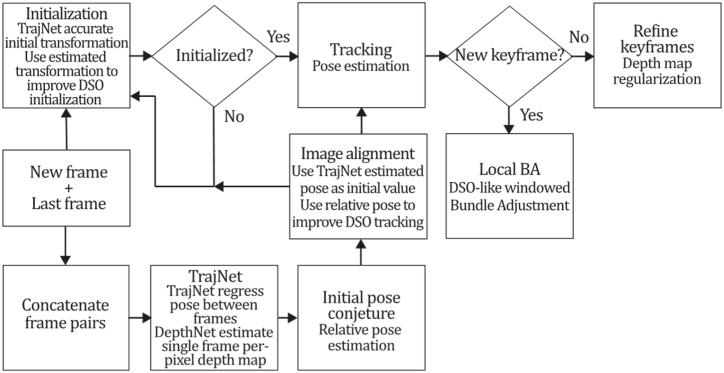
Diagram of MonoRec algorithm. Adapted from Ref. [182].

In DSO, the Gauss-Newton algorithm optimizes the total photometric error of a sliding window of 5–7 keyframes. Then, the whole process can be considered a nonlinear optimization problem, so an initial transformation should be provided, and it will be iteratively optimized; hence, DSO's original proposal uses a unit matrix as the initial transformation. Furthermore, when tracking is lost, DSO initializes three motion models and 27 small rotations when image alignment fails, which is a complex process. DDSO TrajNet provides initial transformations and models for failure modes to overcome these issues. Experimental results proved that TrajNet integration allowed DDSO to outperform the original DSO system, allowing it to obtain robust and accurate trajectories and depth maps without complex calibration. Fig. 51 presents the DDSO algorithm inspired by the article [145].

### 7.3 ML + hybrid methods

Like traditional methods, there is a hybrid category for techniques that effectively combine direct and indirect principles. In this case, we considered the SVO [23] system, which combines direct pixel information extraction with feature extraction to perform both camera tracking and sparse depth map creation. In recent years, this system has been updated, integrating a CNN to enhance its generalization and initialization capabilities discussed in the next section. Despite the reduced number of works in this category, the scientific community has received this category well, being widely used for robotic implementations, thus reaching high citation scores.

**CNN-SVO (2019).** The implementation of depth filters in the SVO mapping thread and the proposed use of direct pixel matching in the semi-direct framework of SVO have enabled the system to achieve efficient camera motion estimation at high frame rates. However, this proposal still has shortcomings, especially regarding the high-depth uncertainty in the map point initialization process. This problem is addressed in the study of [66], where authors incorporated a CNN to overcome this depth uncertainty limitation. The system was built entirely over the SVO framework [23], adding prior depth knowledge obtained by the single-image CNN MonoDepth of [187] for reducing the uncertainty in identifying feature correspondences, which was built over the Resnet50 backbone using a variant of its encoder-decoder architecture. SVO's original proposal divided the system into mapping and tracking threads. Depth values for each feature are obtained by finding feature correspondence over the epipolar line, recovering depth by triangulation. The mapping thread runs the initialization of new map points with high depth uncertainty, updating this depth uncertainty using depth filters created to approximate the mean and variance of current depth values to separate inliers from outliers; hence, a depth filter converges when a point depth uncertainty is small. However, in the original SVO, depth uncertainty tends to be large, leading to two problems: erroneous feature correspondence on the epipolar line and many depth estimations far from converging to their true depth. Consequently, the CNN depth prediction is used to estimate better the mean and variance used in each depth filter, allowing for faster and more accurate convergence.

**Fig. 52:**
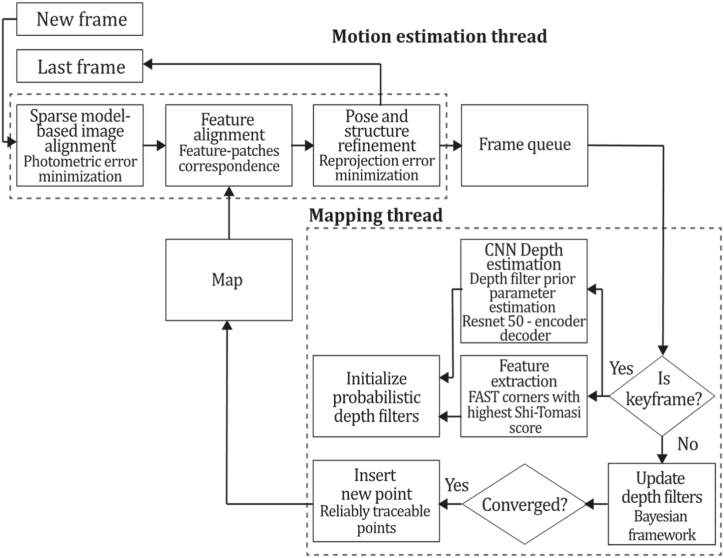
Diagram of CNN-SVO algorithm. Adapted from Ref. [66].

As shown in Fig. 52, MonoDepth is added as a depth estimation module in the mapping thread, providing strong depth priors in the map points initialization process to initialize the depth filters, whereas the original SVO proposal initialized depth filters as average depth measurements of current image $\mu_{n}=1/d_{avg}$ and variance were set as a function of the minimum depth of the image $\sigma_{n}^{2}=1/{\left(6d_{\min}\;\right)}^{2}$; CNN-SVO replaced these simple values with more precise information of the depth estimation coming from the CNN for each filter position as $\mu_{n}=1/d_{CNN}$, $\sigma_{n}^{2}=1/{\left(6d_{CNN}\;\right)}^{2}$. Experimental results demonstrated that adding prior CNN depth information improved the system's performance for overexposed and underexposed images thanks to its illumination invariance properties, facilitating feature correspondence between views and overcoming key illumination issues of original SVO. Fig. 52 introduces the CNN-SVO algorithm inspired by the article [66].

### 7.4 General comments for ML approaches

As previously mentioned, machine learning techniques, particularly convolutional neural networks, have been extensively incorporated into various traditional proposals to address the issues and failure modes identified in each taxonomy category. It is worth noting that, as cited in works [104,163], some researchers believe that classic geometric approaches still outperform ML methods regarding reconstruction quality or tracking.

However, as analyzed in this article, machine learning researchers have attempted to address this issue by developing and implementing new convolutional neural network architectures and creating more robust datasets to improve the training process. On the other hand, as demonstrated in many of the reviewed systems above, CNN can be used not only to recover scene depth or camera pose but also to densify depth maps, estimate initialization parameters, perform preprocessing steps such as feature extraction or optical flow estimation, and perform additional tasks such as semantic segmentation. Thus, from diverse perspectives, CNN can positively contribute to SLAM, VO, and SFM. Another broadly cited problem of machine learning proposals is overfitting [171], as the authors mentioned above keep contributing to the strengthening of different datasets suggesting new datasets like [8,79,154,[156], [157], [158],^188^] constantly facilitating researchers to perform more robust training processes and to incorporate more sophisticated training to their systems in the form of semi-supervised [182] and self-supervised approaches [5,132,180,189]. Similarly, some authors have reported problems related to the generalization of ML methods [60], which has been addressed by many researchers combining the use of datasets recorded in different situations like indoors [79], outdoors [127], autonomous driving [7] and even MAV [78] flying sequences. Thus, we can conclude that ML methods have explored various alternatives to solve the 3D reconstruction problem, making outstanding contributions to this research field. Furthermore, depending on the application that a SLAM, VO, or SFM system will have, we have gathered the same selection criteria described in classic approaches, adding specific criteria only applicable to ML systems, which are the CNN architectures that were integrated and the main estimation tasks that motivated the use of CNN for each system. Table 3 illustrates information gathered for 11 criteria reviewed in each ML system.

**Fig. 53:**
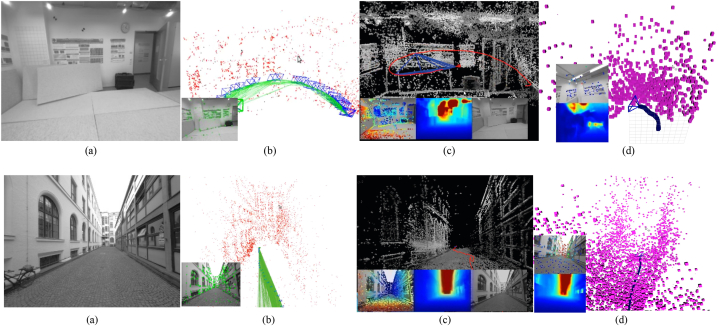
Examples of results obtained by ML approach implementations. (a) represents the input image, (b) presents results obtained using the DynaSLAM (indirect + sparse) algorithm [192], (c) presents results obtained using the CNN-DSO (direct + sparse) algorithm [197], (d) presents results obtained using the CNN-SVO (hybrid) algorithm [196]. Top row results correspond to the indoor example sequence seq_01, and bottom row results correspond to the outdoor sequence seq_29 of the TUM-MONO dataset [127]. The examples were obtained through multiple executions of each algorithm in our previous comparative work [107]. For further information on the implementation and performance of each algorithm and category, we encourage the reader to address the paper [128] and the repository: https://github.com/erickherreraresearch/MonocularPureVisualSLAMComparison [129].

Additionally, we have run some implementations of the ML methods available as open-source code. Some examples of the implemented algorithm executions are shown in Fig. 53, where: Fig. 53.a. represents the input image, Fig. 53.b. presents results obtained using the DynaSLAM (indirect + sparse) algorithm, Fig. 53.c. presents results obtained using the CNN-DSO (direct + sparse) algorithm, and Fig. 53.d. presents results obtained using the CNN-SVO (hybrid) algorithm. The implemented examples were selected due to their availability and the capacity to run depending on the monocular RGB input modality as the unique source of information. It must be mentioned that methods like [2,10,59,134,139,141,145,163,171,182,190,191] were made publicly available by their open-source codes, but their open-source published version did not include their monocular RGB pipeline and they depend on additional information, like externally estimated optical flow or depth prior information; thus they were not included in Fig. 53 examples. Fig. 53 illustrates results obtained by implementing ML monocular SLAM, VO, and SFM systems over publicly available datasets.

## 8 Discussion

SLAM, VO, and SFM are problems that have been extensively studied over the last three decades, particularly in the last two decades, due to the limited computational power available to perform their constituent tasks. However, it should be noted that there are still unresolved issues that have motivated the development of the previously described systems. These include reinforcing existing systems, optimizing computational resources, adding scene comprehension capabilities, tracking and reconstructing moving objects, increasing map density, improving generalization capabilities for unseen environments, achieving per-pixel dense scaled reconstruction, and others. For instance, reinforcement of currently available methods has been extensively studied by authors like [27,30,38,64,81] working on original systems still implementing classic techniques and implementing neural networks over classic implementations [2,6,66,110,138,139,146,171]. Optimization of computational resources has been studied mainly for those implementations made over embedded devices with little computation capacity, like UAVs or drones, where authors had to implement unique feature, point, and frame selection strategies to work on a reduced amount of information [6,23]. The addition of scene comprehension capabilities is addressed in classic methods like [38,81] adding scene comprehension modules, or using machine learning to label point sets belonging to that the network was previously trained to recognize [2,146]. Moving object detection and depth prediction have also been studied by authors like [182,198], who have used artificial intelligence to create masks to determine the probability of each pixel belonging to moving objects, favoring object detection, removal, and prediction. The increase of map density has also been addressed by using classic dense techniques based on the optical flow [85,91] or pixel intensities [4,27,37], even using neural networks trained for this purpose [6,60]. Authors like [58] proposed using multiple datasets in the training stage to increment generalization capabilities. In contrast, others, like [182], used multistage training techniques, and others applied ML regularization and data augmentation techniques. Moreover, the goal of recovering scene scale from monocular images had been studied using neural networks especially trained for such [60,174,193] or by including this parameter on the CNN inference model.

Despite the numerous proposals made by authors to overcome the failure of monocular 3D reconstruction modalities and open problems, many possibilities and combinations still require further study. Additionally, there are many more possibilities beyond this overview's scope. Therefore, we have gathered cite score information of the methods reviewed in the previous sections, providing the reader with a sense of evolution in research fields.

The information presented and discussed in the following sections was gathered using the Scopus database, per-year citation metrics, and the taxonomy proposed in this paper. The data was structured in a data frame considering the categoric variables: method name, whether it was a Classic or ML method, its dense or sparse classification, its direct or indirect classification, and its taxonomy final category. The data frame also considered numeric variables for the year, and the number of cites the article has received each year since its publication. The citation score variable was normalized using the preProcess function of the caret R package, which uses the Min-Max Scaling method to allow the data transform to the scale from 0 to 1, which is compatible with the selected trend identifying technique. To identify the trend of each time series, we employed the prophet function of Facebook's Prophet package in R which took the normalized citing score and the year as inputs, giving the trend and yearly components as outputs, from where we captured the trend, which was finally rescaled and plotted using Splines with Loess method for ease of visualization. Considering the limited amount of existing data (from 2005 to 2023) and the discrete nature of the dependent variable, trends and patterns were easy to visualize since there were no observed considerable fluctuations or randomness in the data; thus, the additive decomposition assumption was accepted. Nevertheless, due to the small sample size available, these observed patterns and trends cannot be considered for inferential purposes; thus, their use is limited to an interval from 2005 to 2023. In addition, it must be mentioned that forecasting and an in-depth time series analysis of the presented data are out of the scope of this study.

### 8.1 Classic vs. machine learning

**Fig. 54:**
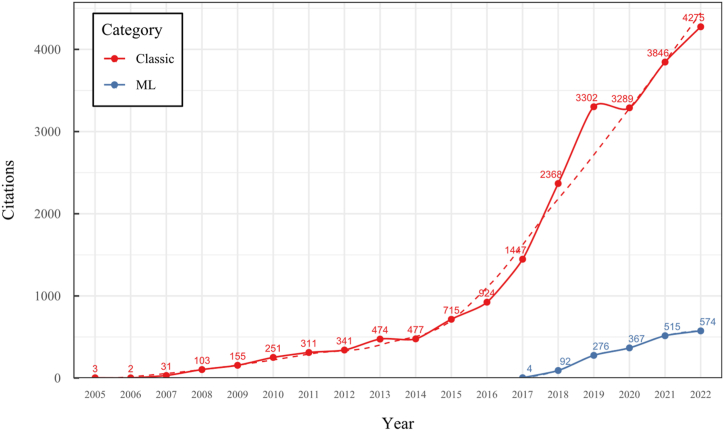
Citation score evolution for classic and ML methods since 2005. Dashed lines represent the trend line for each classification obtained, setting a 99 % confidence interval and the Loess method.

**Fig. 55:**
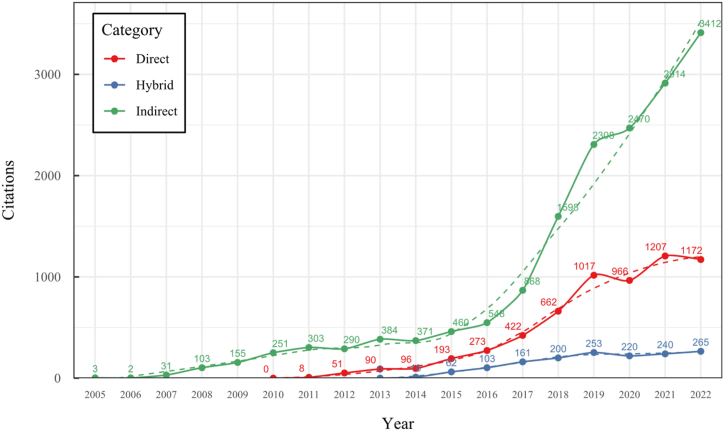
Citation score evolution for direct, indirect, and hybrid methods since 2005. Dashed lines represent the trend line for each classification obtained, setting a 99 % confidence interval and the Loess method.

**Fig. 56:**
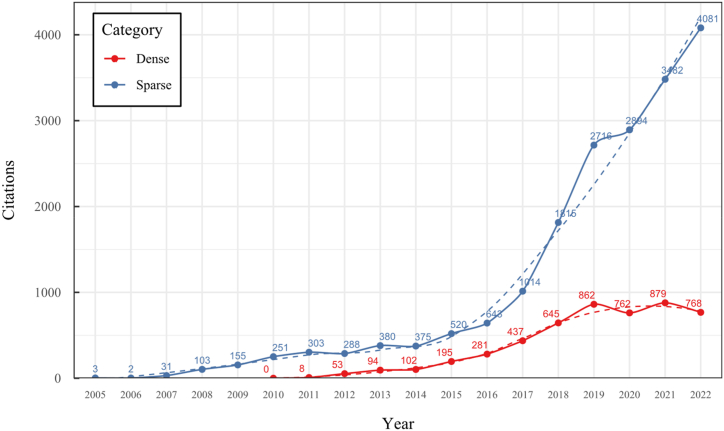
Citation score evolution for dense and sparse methods since 2005. Dashed lines represent the trend line for each classification obtained, setting a 99 % confidence interval and the Loess method.

**Fig. 57:**
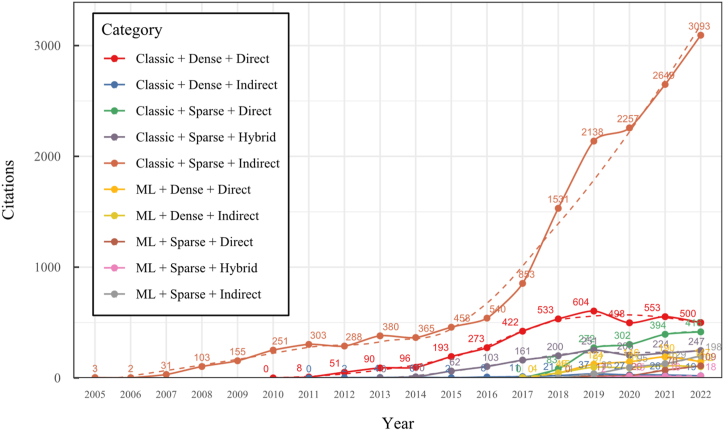
Citation score evolution for taxonomy proposed categories over time. Dashed lines represent the trend line for each classification obtained, setting a 99 % confidence interval and the Loess method.

Beginning with the primary classification of classic and machine learning (ML) methods, Fig. 54 displays citation scores for each category from 2005 to 2022. It is important to note that metrics from 2000 to 2005 (the year of the first cited system release [56]) were not provided due to their low citation score. Additionally, we did not include data for incomplete years, so metrics for 2023 were not considered for this analysis, as the year had not yet ended at the time of article submission. Ther citation scores presented in Fig. 54, Fig. 55, Fig. 56, Fig. 57 correspond to the number of citations each study has gathered since its year of publication.

As evidenced in Fig. 54, over the last 18 years, classic methods have achieved higher citation scores attributed to the extensive time interval that these methods have been available, most of them as open-source implementations. It can be noticed that ML methods started to attract the research community's attention in 2017 as pertaining citation scores kept increasing over time. We believe this trend will continue, so we expect ML citation scores to be close to the impressive values achieved by classic methods shortly.

### 8.2 Direct vs. indirect

Regarding the direct vs. indirect classification, common issues can be highlighted in both categories. Indirect methods mostly rely on geometric Bundle Adjustment based on reprojection error. However, some authors like [24,59] have pointed out that this procedure has drawbacks, such as relying only on the information corresponding to their feature types (such as corners, blobs, or line segments). Feature matching between frames can also introduce a significant number of outliers. As mentioned in Refs. [11,24,59], direct formulations are sensitive to initialization due to the photometric effect of increasing non-convexity. Direct formulations are also sensitive to camera exposure and changes in white balance, being more sensitive to outliers (like motion blur or moving objects). In this way, many formulations have emerged to overcome the issues of each modality getting benefits from both. For example, some indirect implementations, like [59], introduced direct pixel information to densify the recovered depth maps while preserving an indirect backend. Indeed, there are direct formulations like [30] that implemented additional feature extraction techniques enhancing tracking capabilities. Again, this was formally addressed by hybrid self-denominated semi-direct approaches [30,138], where the main difference with proposals like [30,59] resides in the ability to fuse both modalities in their optimization backend completely. The categories were processed with data from the Scopus database, so a citation progress analysis was performed over the years. Fig. 55 represents the direct vs. indirect evolution classification from 2005 to 2022.

Fig. 55 shows that indirect methods are the most cited of the three taxonomy categories, closely followed by direct and hybrid approaches in the third position. This behaviour may be attributed to the ample time interval that indirect methods have been studied, being the first type of proposals that appeared in this research field. In contrast, the first direct processes were released in 2010, and hybrid techniques were addressed in 2014. It must be pointed out that, in this study, we have addressed 16 indirect, 17 direct, and two hybrid methods, so it is noticeable that hybrid approaches have reached an impressive number of citations despite the low number of works available in this category.

### 8.3 Dense vs. sparse

Next are dense and sparse categories for the third component of the proposed taxonomy, which relies on the number of points that conform to the final 3D reconstruction. As mentioned in works like [4,10,23,27,65,66,85,88,99,193,199], the selection process for each of them strongly depends on the final application for which some 3D reconstruction systems may be used or created. For instance, if the application requires maps as dense as possible for navigation [7,182], exploration [5,6,16,18] or augmented reality applications [41,114], then a dense approach is appropriate. However, if the application does not require high 3D reconstruction definition or is required for fast movement [11,23] or little computer capabilities processors applications [6,23,60], then a sparse system is suitable. Following the same process used for the remaining classifications, the database was processed using dense vs. sparse classification as a categorical variable. The results are presented in Fig. 56.

### 8.4 Complete taxonomy

Finally, for the sake of completeness, we analyzed the dataset using the complete taxonomy as a categorical variable. As established in section 5, this taxonomy comprises ten levels produced by combining the three classifications in every possible configuration. Classic + Dense + Direct, Classic + Sparse + Direct, Classic + Dense + Indirect, Classic + Sparse + Indirect, Classic + Hybrid, ML + Dense + Direct, ML + Sparse + Direct, ML + Dense + Indirect, ML + Classic + Sparse + Indirect and ML + Hybrid. Fig. 57 describes the evolution of the taxonomy in each category of citations over time.

**Fig. 58:**
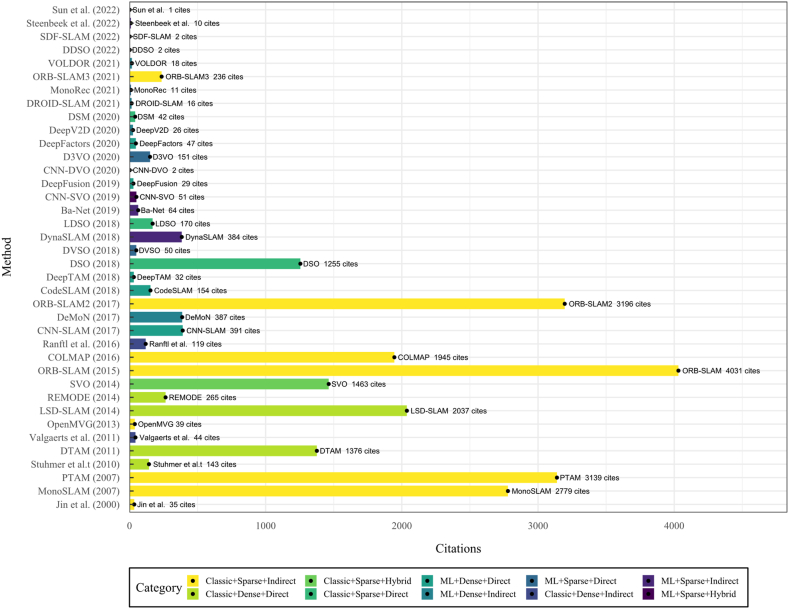
Reviewed methods citation score. The right legend depicts a colour for each taxonomy classification ordered from the highest cited category to the lowest. The methods are ordered on the vertical axis according to their year of appearance.

As evidenced in Fig. 57, the classic sparse indirect category achieved the highest citation scores, which remains the category that has most attracted the attention of researchers and inspired the most representative formulations. This behaviour may be attributed to the early availability of impressive methods like MonoSlam, PTAM, and ORB-SLAM [11,28,41] and the availability of their open-source codes, enabling their use in many implementations or for comparison with new proposals. In addition, the second most cited category is classic dense direct again, the high citation scores achieved may be caused by its capability to overcome one of the most critical issues of sparse indirect systems, which is the ability to recover well-defined depth maps that can be used for a large variety of applications. Finally, the third place is occupied by a relatively recent classic sparse direct approach (2018) that has attracted considerable attention from the scientific community. This behaviour could be motivated by the impressive results obtained by the systems derived from DSO [24], which significantly overcame the issue of noise introduced by direct pixel information extraction while keeping the set of points small enough to be processed with low computational resources. Thus, the effect observed in the citation scores of the different categories in taxonomy can be explained because of outstanding works that have significantly contributed to the state-of-the-art like MonoSlam, PTAM, DTAM, LSD-SLAM, REMODE, SVO, ORB-SLAM, DSO, among others. Fig. 58 lists systems reviewed as yearly released in this article with their corresponding citation scores.

### 8.5 Comparative analysis

In our previous study [128], we compared the most important publicly available monocular SLAM, VO, and SFM methods suitable for 3D reconstruction tasks. For that comparison, we selected the most representative works for each category: indirect + sparse [38], indirect + dense [82], direct + dense [3], direct + sparse [24,30,64], hybrid, and three machine learning extensions of the classic approaches [66,102,197]. We extensively tested each algorithm in the most complete monocular pure visual dataset, the TUM-mono dataset [127], which includes 50 sequences for outdoor and indoor environments, covering a wide range of motion patterns. Following the recommendations of [25,127,200], we performed ten executions of each sequence forwards and backwards, giving 1000 executions for each algorithm and a total of 10000 observations that allowed us to build a large database which was used to perform a statistical analysis of the metrics proposed in the benchmark. The metrics considered the translation error $e_{t}$, rotation error $e_{r}$, scale error $e_{s}$, alignment error $e_{align}$ for the start and end segments and the translational RMSE $e_{rmse}$. The gathered data was evaluated using the accumulated error and motion bias plots proposed by Ref. [127], and then each variable average behaviour was tested using the Kruskal-Wallis and the pairwise Wilcoxon signed-rank tests. Table 4 presents an extract of the results obtained in the study [128].

**Table 4:** Medians and Kruskal-Walli's comparisons for the error metrics of the TUM-mono benchmark. Adapted from Ref. [128].

Method	Translation error	Rotation error	Scale error	Start-segment alignment error	End-segment alignment error	RMSE
Kruskal-Wallis general test	$\chi^{2}=3582.9$$p_{val.}=2.2e-16$	$\chi^{2}=2278.4$$p_{val.}=2.2e-16$	$\chi^{2}=2419.1$$p_{val.}=2.2e-16$	$\chi^{2}=4575.7$$p_{val.}=2.2e-16$	$\chi^{2}=3718$$p_{val.}=2.2e-16$	$\chi^{2}=530.78$$p_{val.}=2.2e-16$
DSO (Dir. + Spa.)	0.8064585^a^	**0.8800369**^**b**^	1.064086^ab^	**0.003974759**^**a**^	**0.004184367**^**a**^	0.1950799^ab^
LDSO (Dir. + Spa.)	**0.7892125**^**a**^	0.9135608^ab^	1.061302^ab^	0.007925665^b^	0.008009198^b^	**0.1944492**^**a**^
CNN-DSO (Dir. + Spa. + ML)	0.7980411^a^	0.9618528^a^	**1.058849**^**a**^	0.008987173^b^	0.006199582^c^	0.2083872^ab^
DSM (Dir. + Spa.)	0.8519143^b^	1.1117710^c^	1.064615^b^	0.015794222^c^	0.015537213^d^	0.2167750^b^
DynaSLAM (Ind. + Spa. + ML)	1.7473504^c^	1.5730542^d^	1.126499^c^	0.004286919^a^	0.005516179^e^	0.2389837^cd^
ORB-SLAM2 (Ind. + Spa.)	2.8738313^d^	2.3585843^e^	1.260155^d^	0.004311949^a^	0.005102672^e^	0.3165024^e^
CNN-SVO (Hybrid + ML)	1.6248001^c^	1.4159545^d^	1.086399^e^	0.067201999^d^	0.062036008^f^	0.2373532^c^
DF-ORB-SLAM (Ind. + Den.)	3.6423921^e^	3.4940400^f^	1.238232^f^	0.053360456^e^	0.084420570^g^	0.3643844^e^
SVO (Hybrid)	5.4819407^f^	3.3772024^f^	1.343603^g^	0.108150349^f^	0.117753996^h^	0.3642558^e^
LSD-SLAM (Dir. + Den.)	9.1403348^g^	14.9621188^g^	2.044298^h^	0.158469383^g^	0.190127787^i^	0.3507099^d^

Means with different letters in the same column differ significantly according to the Kruskal-Walli's test, and pairwise Wilcoxon signed rank test for $p_{value}\leq 0.05$.

As shown in Table 4, the sparse direct methods present clear advantages over the other evaluated methods, registering a significant error reduction compared to the rest of the classic taxonomy. Thus, it is evident that the introduction of the DSO method represents one of the most important contributions in this research field, allowing for impressive SLAM development and VO systems and a considerable error reduction, which directly contributes to enhancing the precision of these systems. Moreover, it can be noticed that the addition of ML modules in classic pipelines significantly improved the performance of the classic methods, which is evidenced by a critical error reduction in the CNN-SVO and DynaSLAM methods when compared with their classic versions. For further details on the experimental protocol and the comparative results, we encourage the reader to address the paper [107] and the repository [108].

From all the information detailed in this work, we can provide the reader with a summary of the main advantages and disadvantages that have been identified for each category of the proposed taxonomy, according to what the authors detailed in their publications and with the experience that we gathered by implementing more than ten publicly available methods. The main benefits and shortcomings of each category are summarized in Table 5.

**Table 5:** Pros and shortcomings for each category of the proposed taxonomy.

Category	Pros	Cons
Classic + Indirect + Sparse	Computationally efficient by using only a subset of features. Feature extraction enhances matching across frames. Preprocessing refinement produces high accuracy.	Sparse reconstructions can suffer from a lack of detail critical for some applications. Dependent on repeatability and invariance of extracted features. Fail in low texture environments lacking features. Some of these methods may produce localization errors when visiting low-textured areas.
Classic + Indirect + Dense	Commonly utilises optical flow to maximize pixel data for dense reconstructions. Optical flow provides motion cues to aid tracking and mapping. Enhanced refinement through optimization techniques using optical flow information.	Optical flow estimation is a computationally expensive preprocessing step. Performance relies heavily on optical flow accuracy. Sensitivity to scenarios with independently moving objects.
Classic + Direct + Dense	Able to utilize most pixel data, leading to more detailed reconstructions. Bypass the need for feature extraction or optical flow estimation steps. Can operate directly on photometric data for estimating geometry.	Rely on brightness constancy assumption, prone to failure with illumination changes. Require precise initialization due to non-convex optimization. Computationally intensive due to large data volumes. A large amount of information implies large outlier volumes, so robust and efficient filtering techniques are required.
Classic + Sparse + Direct	Avoid relying on geometric priors that may introduce bias. Able to sample pixels with various intensity gradients, capturing edges, corners, textures, and many other types of information. Can operate directly on photometric data for estimating geometry. More efficient computation compared to dense methods.	Sparse reconstructions can suffer from a lack of detail critical for some applications. Susceptible to noise without dense pixel information. Struggle with texture-less regions lacking gradients.
Classic + Hybrid	Combines the benefits of both direct pixel data and feature extraction. Proficient tracking from direct alignment and feature matching. Computationally efficient compared to purely dense methods.	Complexity in integrating both modalities into a unified backend. Potentially suboptimal performance compared to dedicated direct/indirect methods. Very few exemplar methods exist currently. Algorithms may present relocalization and loop closure errors in texture-less scenarios.
ML + Indirect + Sparse	Augments feature extraction, description and matching. Provides strong priors to aid SLAM initialization and scale recovery. Maintains efficiency of sparse optimization backend.	Sparse reconstructions can suffer from a lack of detail critical for some applications. Risk of overfitting with limited training data complexity. Generalization remains a challenge. A sparse set of points based on feature matching can be insufficient to recover accurate scales and reconstructions.
ML + Indirect + Dense	Integration of learning enhances optical flow accuracy. CNNs boost feature extraction, matching, and pose estimation. More robust to challenging lighting conditions and motion patterns compared to classical flow. Enhanced refinement through optimization techniques using optical flow information.	Computationally expensive due to dense flow estimation. Complexity in integrating CNN outputs into optimization backend. Generalization can be poor when exploring environments or motion patterns considerably different from the training data.
ML + Direct + Dense	CNNs can enhance initialization and handle photometric variations. End-to-end learning is feasible for pose and depth estimation. They can recover denser reconstructions. CNNs can be used to overcome scale ambiguity and densify the reconstructions based on pixel information.	Still rely on brightness constancy assumption. Susceptible to overfitting due to limited training data complexity. Generalization can be poor in novel environments. High computational cost due to large data volumes. Still susceptible to large amounts of outliers.
ML + Direct + Sparse	CNN integration improves initialization and tracking robustness. Depth priors from CNNs mitigate scale ambiguity. Maintain computational efficiency of sparse methods. A reduced set of filtered pixel information reduces computational complexity. Less susceptible to outliers compared to dense methods. Reconstruction quality and CNN performance benefit considerably from a good point selection strategy.	Restricted by sparsity, lacking detail in reconstructions. Generalization remains a challenge. Access to training data with ground truth depth is limited. Susceptible to noisy information coming from the CNNs. CNN integrations can only contribute in scenarios similar to training data.
ML + Hybrid	Combines the benefits of learning and direct alignment techniques. Improves initialization while retaining tracking precision. Efficient compared to dense learned methods. More stable performance compared to classic hybrid techniques.	Very few exemplar methods exist currently. Complexity in integrating modalities into a unified backend. Generalization can be poorer than dedicated learned methods. Algorithms may present relocalization and loop closure errors in textureless scenarios. Considerably limited to producing sparse reconstructions due to their indirect modules.

Table 5 shows that each category has its own set of advantages and issues inherent to the framework's nature. This indicates a trade-off between sparsity and preprocessing. Sparser methods result in faster and lighter performance due to the limited information, but the final reconstruction may lack crucial details which are significant for certain applications. Likewise, preprocessing entails additional steps, varying in computational workload. If preprocessing is focused on reducing the amount of information, like feature extraction, then the method will become lighter and faster, but it may present issues in texture-less areas due to the lack of corners and edges commonly tracked as features.

However, optical flow can provide more information at the cost of high computational requirements. Therefore, many authors have contributed to ML extensions, employed CNNs to densify final reconstructions, or used lighter ML alternatives to recover optical flow. Finally, it must be mentioned that a light advantage still exists in using classic techniques due to the large number of publicly available systems for implementation and testing. This also represents a scalability advantage for the classic methods, considering their availability and multiple researchers' reports of their ease of integration with existing ML modules to contribute to multiple tasks.

## 9 Conclusions

Scene 3D reconstruction is a complex problem that can be addressed using various techniques and technologies. This work provides an overview of one of the most attractive alternatives to 3D reconstruction: the visual reconstruction of an environment using a monocular RGB camera as the sole source of information. We discussed several input modes and their advantages and disadvantages, considering three main techniques applicable to this task (SLAM, VO, and SFM) and establishing a taxonomy containing the most likely system configurations in the literature. Three classifications were defined, providing ten possible combinations comprising an extended taxonomy. Also, 42 of the most representative monocular systems were reviewed comprehensively. In order to guide the selection and application of such systems, we have gathered nine criteria for each classic system being decision components for the implementation of a 3D reconstruction system, which are: type of algorithm, tracking method, map density, pixels used, estimation method, global optimization, relocalization, loop closure, and availability. We have gathered eleven criteria for ML methods, the same as classic procedures, although introducing two additional criteria for the network: CNN architecture and CNN main estimation tasks.

Furthermore, researchers can use the comprehensive information gathered to select the most suitable algorithm or taxonomy category for their projects. Additionally, we have discussed the main strengths and shortcomings of each of the classifications that comprise the taxonomy, and we have analyzed the progress of each classification and category of the taxonomy in the last 18 years according to their citation scores that give an intuition of the impact and acceptance that each of them has produced in this research field. Finally, for future work, we will use the proposed taxonomy to compare each category's most representative open-source algorithms to determine their advantages and disadvantages to select the more suitable methodology for monocular 3D reconstruction of indoor scenes.

## Data availability statement

Comparative data and 20 video examples of multiple algorithm executions are publicly available in the repository: https://github.com/erickherreraresearch/MonocularPureVisualSLAMComparison.

## Disclosure statement

Part of this article is part of the work presented in Erick Herrera-Granda's Ph.D. thesis entitled "Real-time Monocular 3D Reconstruction of scenarios using artificial intelligence techniques", submitted to University of Granada in 2024.

## CRediT authorship contribution statement

**Erick P. Herrera-Granda:** Writing – review & editing, Writing – original draft, Investigation, Formal analysis. **Juan C. Torres-Cantero:** Supervision, Project administration, Formal analysis. **Diego H. Peluffo-Ordóñez:** Visualization, Validation, Resources, Project administration, Funding acquisition.

## Declaration of competing interest

The authors declare that they have no known competing financial interests or personal relationships that could have appeared to influence the work reported in this paper.
